# Maternal diet during pregnancy is related with the infant stool microbiome in a delivery mode-dependent manner

**DOI:** 10.1186/s40168-018-0490-8

**Published:** 2018-07-05

**Authors:** Sara N. Lundgren, Juliette C. Madan, Jennifer A. Emond, Hilary G. Morrison, Brock C. Christensen, Margaret R. Karagas, Anne G. Hoen

**Affiliations:** 1 Department of Epidemiology, Geisel School of Medicine at Dartmouth, Hanover, NH USA; 2 Division of Neonatology, Department of Pediatrics, Children’s Hospital at Dartmouth, Hanover, NH USA; 3 Children’s Environmental Health & Disease Prevention Research Center at Dartmouth, Lebanon, NH USA; 4 Department of Biomedical Data Science, Geisel School of Medicine at Dartmouth, Hanover, NH USA; 5 Josephine Bay Paul Center, Marine Biological Laboratory, Woods Hole, MA USA; 6 Department of Molecular and Systems Biology, Geisel School of Medicine at Dartmouth, Hanover, NH USA; 7 Center for Molecular Epidemiology, Geisel School of Medicine at Dartmouth, Hanover, NH USA

**Keywords:** Infant gut microbiome, Infant gut microbiome clusters, Maternal prenatal diet, Delivery mode, 16S rRNA gene, Fruit

## Abstract

**Background:**

The gut microbiome has an important role in infant health and immune development and may be affected by early-life exposures. Maternal diet may influence the infant gut microbiome through vertical transfer of maternal microbes to infants during vaginal delivery and breastfeeding. We aimed to examine the association of maternal diet during pregnancy with the infant gut microbiome 6 weeks post-delivery in mother-infant dyads enrolled in the New Hampshire Birth Cohort Study. Infant stool samples were collected from 145 infants, and maternal prenatal diet was assessed using a food frequency questionnaire. We used targeted sequencing of the 16S rRNA V4-V5 hypervariable region to characterize infant gut microbiota. To account for differences in baseline and trajectories of infant gut microbial profiles, we stratified analyses by delivery mode.

**Results:**

We identified three infant gut microbiome clusters, characterized by increased abundance of *Bifidobacterium*, *Streptococcus* and *Clostridium*, and *Bacteroides*, respectively, overall and in the vaginally delivered infant stratum. In the analyses stratified to infants born vaginally and adjusted for other potential confounders, maternal fruit intake was associated with infant gut microbial community structure (PERMANOVA, *p* < 0.05). In multinomial logistic regression analyses, increased fruit intake was associated with an increased odds of belonging to the high *Streptococcus*/*Clostridium* group among infants born vaginally (OR (95% CI) = 2.73 (1.36, 5.46)). In infants delivered by Cesarean section, we identified three clusters that differed slightly from vaginally delivered infants, which were characterized by a high abundance of *Bifidobacterium*, high *Clostridium* and low *Streptococcus* and *Ruminococcus* genera, and high abundance of the family *Enterobacteriaceae*. Maternal dairy intake was associated with an increased odds of infants belonging to the high *Clostridium* cluster in infants born by Cesarean section (OR (95% CI) = 2.36 (1.05, 5.30)). Linear models suggested additional associations between maternal diet and infant intestinal microbes in both delivery mode strata.

**Conclusions:**

Our data indicate that maternal diet influences the infant gut microbiome and that these effects differ by delivery mode.

**Electronic supplementary material:**

The online version of this article (10.1186/s40168-018-0490-8) contains supplementary material, which is available to authorized users.

## Background

Studies have examined the maternal contribution to infant health, including the effect of maternal diet during pregnancy and lactation. Prenatal diet influences the risk of infant and child allergy. For instance, the Mediterranean diet during pregnancy has been associated with a reduced risk of both persistent and atopic wheeze and atopy in children at 6.5 years old [1, 2]. High meat consumption during pregnancy is associated with an increased risk of wheeze in the first year of life, while maternal dairy intake is associated with a reduced risk of infantile wheeze [2]. Little is known about the actual mechanisms by which maternal diet affects children’s health; we hypothesize that maternal diet impacts the development of the gut microbiome in infancy and subsequently influences child health outcomes. It is established that diet is an important driver of the gut microbiome. Short-term diets comprised solely of either plant or animal foods have been shown to alter the human gut microbiome [3]. In addition, studies in humans and humanized gnotobiotic mice show that diets with reduced carbohydrates [4], or high in polysaccharides, alter gut microbiome composition [5].

To date, there has been limited investigation of the relation of maternal diet with the developing infant gut microbiome. One study observed that maternal high-fat diet during pregnancy was associated with meconium microbial composition but had limited maternal diet data [6]. One mechanism the infant gut microbiome may be affected by maternal diet is via vertical transfer of maternal microbes to infants at delivery. There is a well-established relation of the infant gut microbiome with delivery mode (Cesarean section versus vaginally born infants) [7, 8] which may persist through adulthood [9]. Alternatively, maternal diet may affect fetal development and subsequent host response to microbial populations at and after birth. Additionally, differences in breast milk composition due to maternal diet may contribute to the infant gut microbiome in breastfed infants. We examined the relationship of maternal diet during pregnancy with the infant stool microbiome in 6-week-old infants and identified associations within delivery mode groups.

## Results

### Study population

Characteristics of the study population are summarized in Table 1 (*n* = 145). Maternal age ranged from 22 to 44 years, with an average of 31.9 years. More than 70% of mothers in the population had at least a college degree, more than 90% were married, and most were first-time mothers. Smoking during pregnancy was rare at 4.8%, and the population was slightly overweight with an average pre-pregnancy BMI of 25.6. Our population had more male infants than female infants (57.2% males), and an average birth weight and gestational age of 3427 g and 39.3 weeks, respectively. Most infants were born vaginally (66.9%) and were exclusively breastfed (70.3%) at 6 weeks of age. Daycare attendance and infant antibiotic exposure at or before 4 months of age were rare. Measures and distributions of maternal prenatal diet are shown in Table 1. Most subject characteristics and maternal dietary factors were not different between delivery mode groups (Table 1).

**Table 1:** Subject characteristics by delivery mode (*n* = 145)

	*n* (%) or mean [range]			*p* value^1^
	All (*n* = 145)	Vaginal (*n* = 97)	Cesarean (*n* = 48)	
Maternal characteristics				
Maternal age	31.9 [22–44]	31.8 [22–44]	32.2 [23–42]	0.65
Education level				0.14
Less than 11th grade	1 (0.7)	1 (1.0)	0 (0.0)	
High school graduate or equivalent	15 (10.3)	7 (7.2)	8 (16.7)	
Junior college graduate or some college	25 (17.2)	16 (16.5)	9 (18.8)	
College graduate	54 (37.2)	34 (35.1)	20 (41.7)	
Any post-graduate schooling	50 (34.5)	39 (40.2)	11 (22.9)	
Relationship status				0.29
Married	133 (91.7)	91 (93.8)	42 (87.5)	
Separated or divorced	3 (2.1)	1 (1.0)	2 (4.2)	
Single and never married	9 (6.2)	5 (5.2)	4 (8.3)	
Smoking				0.43
No	138 (95.2)	91 (93.8)	47 (97.9)	
Yes	7 (4.8)	6 (6.2)	1 (2.1)	
Parity	0.8 [0–4]	0.9 [0–4]	0.7 [0–2]	0.07
Maternal BMI	25.6 [17.4–47.8]	26.0 [17.4–47.8]	24.7 [18.4–42.1]	0.22
Gestational diabetes^2^				0.58
No	121 (83.4)	84 (86.6)	37 (77.1)	
Yes	16 (11.0)	10 (10.3)	6 (12.5)	
Dietary factors (servings/day)^3^				
aMED score	3.67 [0–7]	3.8 [0–7]	3.5 [0–7]	0.32
Dairy	3.6 [0.0–8.5]	3.7 [0.0–8.5]	3.5 [0.8–6.8]	0.40
Fruit	2.3 [0.0–5.9]	2.3 [0.2–5.9]	2.2 [0.0–5.6]	0.52
Vegetables	3.3 [0.0–9.6]	3.3 [0.5–9.6]	3.2 [0.0–7.6]	0.78
Whole grains	1.0 [0.0–4.1]	1.2 [0.1–4.1]	0.9 [0.0–3.1]	0.03
Fish and seafood	0.2 [0.0–1.0]	0.2 [0.0–1.0]	0.2 [0.0–0.6]	0.56
Nuts, legumes, and soy	0.9 [0.0–4.5]	0.9 [0.0–3.1]	0.9 [0.0–4.5]	1.00
Red and processed meat	0.8 [0.0–2.3]	0.7 [0.0–2.0]	0.8 [0.0–2.3]	0.21
Polyunsaturated fat (g/day)	13.9 [8.67–23.7]	13.6 [8.7–21.0]	14.5 [9.2–23.7]	0.12
EPA (g/day)	0.1 [0.0–0.5]	0.1 [0.0–0.5]	0.1 [0.0–0.5]	0.28
DHA (g/day)	0.1 [0.0–0.6]	0.1 [0.0–0.6]	0.1 [0.0–0.5]	0.79
Monounsaturated:saturated fatty acid ratio	1.1 [0.7–2.3]	1.1 [0.7–2.1]	1.2 [0.7–2.3]	0.35
Infant characteristics				
Sex				0.38
Female	62 (42.8)	44 (45.4)	18 (37.5)	
Male	83 (57.2)	53 (54.6)	30 (62.5)	
Birth weight (g)^4^	3427.4 [1960–4710]	3466.3 [2030–4565]	3348.9 [1960–4710]	0.18
Gestational age (weeks)	39.3 [30.4–43.4]	39.5 [30.4–42.1]	39.1 [33.4–43.4]	0.21
Feeding method				0.04
Exclusively breastfed	102 (70.3)	74 (76.3)	28 (58.3)	
Combination	37 (25.5)	21 (21.7)	16 (33.3)	
Exclusively formula fed	6 (4.1)	2 (2.1)	4 (8.3)	
Antibiotics by 4 months old^5^				0.66
No	135 (93.1)	91 (93.8)	44 (91.7)	
Yes	5 (3.5)	3 (3.1)	2 (4.2)	

^1^*p* value determined by two-sided Welch’s *t* test or Fisher’s exact test

^2^Missing data on eight subjects

^3^All dietary factors are standardized to a 2000 cal/day diet, excluding aMED score and monounsaturated:saturated fatty acid ratio

^4^Missing data on three subjects

^5^Missing data on five subjects

### Microbial community composition and maternal dietary factors

**Fig. 1:**
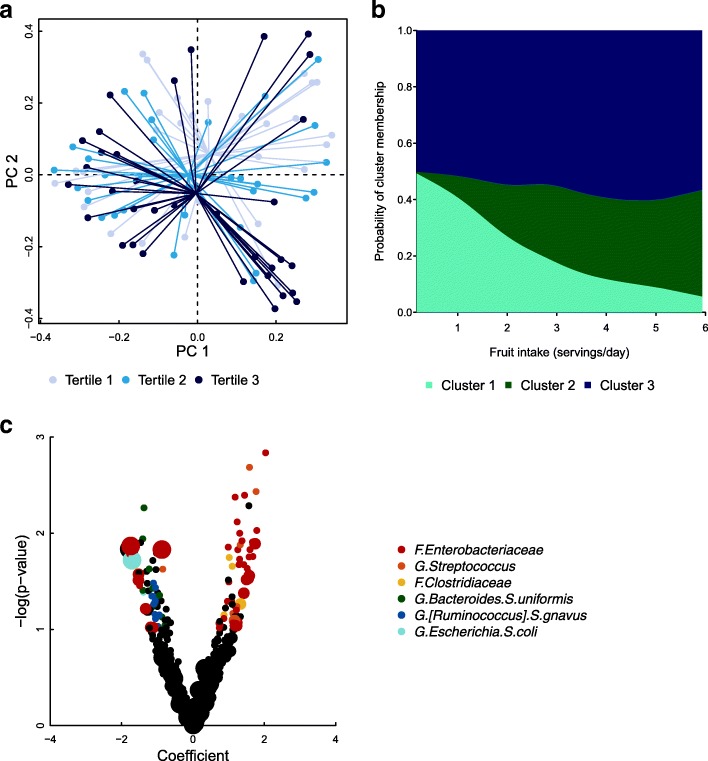
Associations between maternal diet and infant gut microbial communities in infants delivered vaginally. Plots include only infants delivered vaginally (*n* = 97). **a** Principal coordinate plot of generalized UniFrac distances (PERMANOVA *p* = 0.028 for maternal fruit intake as a continuous variable), colored by maternal fruit intake tertiles. Each point represents an individual, and lines indicate the distance from tertile centroid. **b** Predicted probability plot of infant stool cluster membership by maternal fruit intake during pregnancy from multinomial logistic regression models adjusted for infant feeding method, maternal BMI, parity, and batch. Cluster 1 is the reference group. **c** Linear model associations between maternal aMED score and relative abundance of infant stool OTUs. The size of each point indicates the log-ratio transformed relative abundance (LRTA) of each OTU (LRTA ≥ 30, LRTA ≥ 20, or LRTA < 20). Points are colored by taxonomy represented heavily in top results, for *p* < 0.10. *F*., *G*., and *S*. in taxonomy labels indicate that the level of taxonomy is family, genus, or species, respectively

We identified 11,029,093 bacterial sequences in 145 6-week infant stool samples passing quality control. The most abundant taxonomic group was *Enterobacteriaceae* comprising 20.0% of the 6-week infant stool microbiome, followed by *Bifidobacterium* (18.4%), *Bacteroides* (10.4%), and *Streptococcus* (8.10%) (Additional file 1: Table S1). Open-reference OTU picking identified a total of 4260 OTUs in our population of 6-week infant stool samples, with 2850 OTUs corresponding to Greengenes IDs and 1410 de novo OTUs. Our main analyses focused on vaginally delivered infants (*n* = 97). Adjusting for infant feeding method, maternal BMI, parity, and batch, maternal fruit consumption was associated with infant stool microbiome composition (generalized UniFrac distance PERMANOVA, *p* = 0.028; Table 2; Fig. 1a). In a sensitivity analysis, the effect of maternal fruit consumption on the infant gut microbiome in babies born vaginally persisted when restricted to infants who were exclusively breastfed (generalized UniFrac distance PERMANOVA, *p* = 0.022; Table 2*)*. Excluding infants who may have received antibiotics, or those delivered prematurely (Additional file 1: Table S10, Figure S7a), did not qualitatively change the results.

**Table 2:** Relation of microbial community composition in vaginally delivered 6-week old infants with maternal diet

	*p* value^1^	
Dietary factor	All (*n* = 97)^2^	Exclusively breastfed (*n* = 74)^3^
aMED score	0.19	0.26
Dairy	0.60	0.91
Fruit	0.028	0.022
Vegetables	0.56	0.92
Whole grains	0.47	0.25
Fish and seafood	0.33	0.21
Nuts, legumes, and soy	0.52	0.71
Red and processed meat	0.86	0.69
Polyunsaturated fat	0.76	0.84
EPA	0.29	0.45
DHA	0.40	0.50
MUFA:SFA ratio	0.66	0.43

^1^All *p* values are determined by PERMANOVA

^2^*p* values are adjusted for infant feeding method, maternal BMI, parity, and batch

^3^*p* values are adjusted for maternal BMI, parity, and batch

### Infant gut microbiome clusters

We identified three distinct clusters of the infant gut microbiome in vaginally delivered infants (*n* = 97), with cluster 1 characterized by a high abundance of the genus *Bifidobacterium*, cluster 2 by the genera *Streptococcus* and *Clostridium*, and cluster 3 by the genus *Bacteroides* (Additional file 1: Figure S1, Table S1). To assess the relationship between maternal dietary factors and infant gut cluster membership, we used multinomial logistic regression with cluster 1 as the reference group. The odds of belonging to cluster 2 are 2.73 times greater for each additional maternal serving of fruit per day (95% CI 1.36, 5.46; Table 3, Fig. 1b). These results were consistent when infants delivered prematurely were not included in the analyses (Additional file 1: Table S9, Figure S6a).

**Table 3:** Infant gut microbiome cluster is influenced by maternal diet

	OR (95% confidence interval)^1, ^^2^	
Dietary factor	Cluster 2	Cluster 3
aMED score	1.31 (0.90, 1.89)	0.96 (0.73, 1.27)
Dairy	0.87 (0.56, 1.37)	0.84 (0.59, 1.18)
Fruit	2.73 (1.36, 5.46)^3^	1.61 (0.97, 2.68)^3^
Vegetables	0.88 (0.60, 1.28)	0.82 (0.60, 1.11)
Whole grains	0.94 (0.36, 2.41)	1.10 (0.57, 2.13)
Fish and seafood	0.67 (0.01, 32.26)	0.50 (0.02, 12.57)
Nuts, legumes, and soy	0.49 (0.20, 1.20)	0.48 (0.24, 0.96)
Red and processed meat	4.39 (0.82, 23.47)	2.35 (0.62, 8.95)
Polyunsaturated fat	0.84 (0.65, 1.09)	0.87 (0.72, 1.06)
EPA	0.24 (0.00, 72.57)	4.13 (0.08, 217.72)
DHA	0.15 (0.00, 62.82)	2.66 (0.04, 158.91)
MUFA:SFA ratio	1.64 (0.15, 17.73)	1.78 (0.26, 12.28)

^1^Models include vaginally delivered infants (*n* = 97)

^2^Cluster 1 is the reference group

^3^Corresponds to Fig. 1b

### Operational taxonomic units in the infant gut and maternal dietary factors

To investigate the microbial taxa that may drive differences observed above, we measured associations between each maternal dietary factor and the relative abundance of individual infant stool operational taxonomic units (OTUs) with linear models adjusted for feeding method, maternal BMI, parity, and batch. While the microbiome-wide significance of maternal dietary factors after adjusting *p* values using the false discovery rate (FDR) method was not observed, OTUs with the same taxonomic assignment tended to appear together in the most significant results, supporting a true association of maternal dietary factors and infant gut microbiota. Maternal aMED score was positively associated with OTUs classified to the family *Enterobacteriaceae*, the genus *Streptococcus*, and the family *Clostridiaceae*, and negatively with OTUs classified to the species *Bacteroides uniformis*, the *Enterobacteriaceae* family, and the species *Escherichia coli* and *[Ruminococcus] gnavus* (Fig. 1c; Additional file 1: Table S2a). Maternal dairy intake was positively associated with OTUs classified to the species *Clostridium neonatale* and *C. butyricum* and the genus *Staphylococcus*, and negatively related to OTUs classified to the *Lachnospiraceae* family (Additional file 1: Table S2b). OTUs classified to the genus *Streptococcus* including the species *Streptococcus agalactiae* were positively associated with maternal fish and seafood intake, while OTUs classified to the species *Bacteroides uniformis* were negatively associated (Additional file 1: Table S2c). We observed a negative association between fruit intake and OTUs of the genus *Bifidobacterium* and a positive association with OTUs in the *Clostridiaceae* family in the infant gut (Additional file 1: Table S2d). We observed additional associations between these and other maternal dietary factors and infant stool OTUs, which are presented in the supplementary tables.

### Associations in infants delivered by Cesarean section

**Fig. 2:**
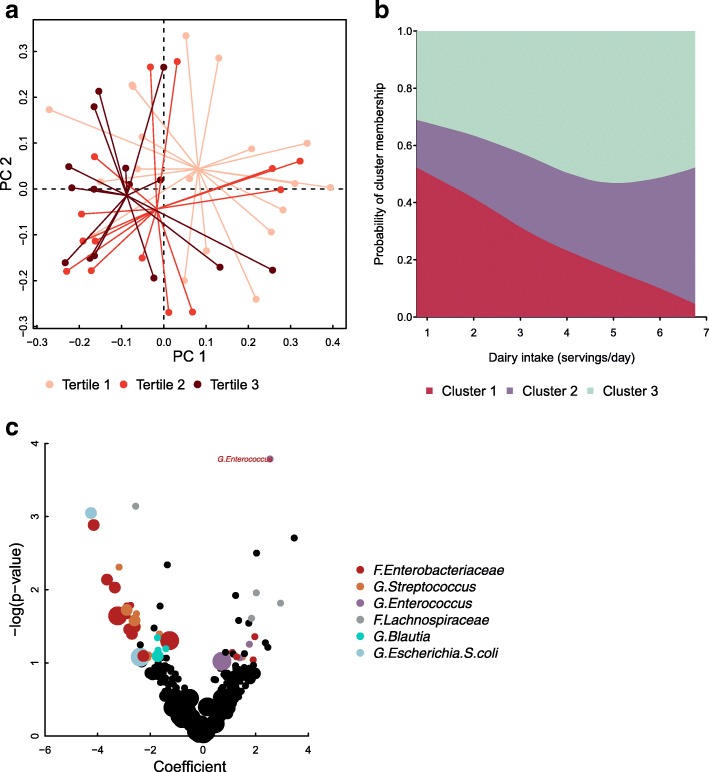
Associations between maternal diet and infant gut microbial communities in infants delivered vaginally. Plots include only infants delivered by Cesarean section (*n* = 48). **a** Principal coordinate plot of generalized UniFrac distances (PERMANOVA *p* = 0.034 for maternal dairy as a continuous variable), colored by maternal dairy intake tertiles. Each point represents an individual, and lines indicate the distance from tertile centroid. **b** Predicted probability plot of infant stool cluster membership by maternal dairy intake during pregnancy from multinomial logistic regression models adjusted for infant feeding method, maternal BMI, parity, and batch. Cluster 1 is the reference group. **c** Linear model associations between maternal aMED score and relative abundance of infant stool OTUs. The size of each point indicates the log-ratio transformed relative abundance (LRTA) of each OTU (LRTA ≥ 30, LRTA ≥ 20, or LRTA < 20). Red text indicates *q* < 0.10. Points are colored by taxonomy represented heavily in top results, for *p* < 0.10. *F*., *G*., and *S*. in taxonomy labels indicate that the level of taxonomy is family, genus, or species

We separately assessed the relation of maternal diet with the infant gut microbiome in infants delivered by Cesarean section (*n* = 48). In an adjusted analysis, maternal dairy intake was associated with infant stool microbiome composition (generalized UniFrac distance PERMANOVA, *p* = 0.034; Fig. 2a; Additional file 1: Table S3). Additionally, we identified clusters of infant gut microbiota in infants delivered by Cesarean section that differed slightly from those identified in vaginally delivered infants; cluster 1 was characterized by a high abundance of the genus *Bifidobacterium*, cluster 2 by high *Clostridium* and low *Streptococcus* and *Ruminococcus* genera, and cluster 3 by high abundances of the family *Enterobacteriaceae*, the genus *Ruminococcus*, and the family *Lachnospiraceae* (Additional file 1: Table S1, Figure S1). The odds of belonging to the cluster 2 were 2.36 times greater for each additional maternal serving of dairy per day (95% CI 1.05, 5.30; Fig. 2b; Additional file 1: Table S4). Linear models indicated a positive association of maternal dairy intake with OTUs classified to the family *Enterobacteriaceae* and the species *Escherichia coli* and negative association with the OTUs that belong to the genera *Bifidobacterium*, *Pseudomonas*, and *Bacteroides* (Additional file 1: Table S5b). One OTU classified to the genus *Corynebacterium* was negatively associated with maternal dairy intake (*p* = 0.0044), and one each OTU classified to the species *Acinetobacter rhizosphaerae* (*p* = 0.0040) and to the family *Ruminococcaceae* (*p* = 0.0061), respectively, were also positively associated with maternal dairy intake, but we did not observe multiple related OTUs with similar associations. OTUs classified to the genus *Enterococcus* (one *q* value < 0.10) and the *Lachnospiraceae* family were positively related with and OTUs classified to the family *Enterobacteriaceae*, the species *Escherichia coli* and the genera *Streptococcus* and *Blautia*, were negatively related with the maternal aMED score (Additional file 1: Table S5a). As in vaginally delivered infants, maternal fish and seafood intake was positively associated with OTUs in the genus *Streptococcus* in infants delivered by Cesarean section (Additional file 1: Table S5c). In contrast to infants born vaginally, maternal dietary fish and seafood were positively associated with OTUs in the genus *Bacteroides* including the species *B. uniformis* and negatively associated with OTUs in the species *Clostridium neonatale* and other OTUs of the *Streptococcus* genus (Additional file 1: Table S5c). Additionally, maternal red and processed meat was positively associated with OTUs in the genus *Bifidobacterium* among others including the species *Escherichia coli* and the genus *Enterococcus* (Additional file 1: Table S5e). Results were qualitatively the same when infants born before 37 weeks gestation were excluded (see Additional file 1: Supplementary Information).

## Discussion

We observed differences in both overall infant gut microbial community structure and specific microbes in relation to maternal dietary factors, often in a delivery mode-dependent pattern. Some effects of maternal diet were more apparent in exclusively breastfed infants; however, we were unable to compare to exclusively formula-fed infants since most infants received some breast milk.

We found maternal fish and seafood consumption to be positively related to OTUs in the genus *Streptococcus* in the infant gut, which included the species *Streptococcus agalactiae*, or Group B *Streptococcus*. *Streptococcus agalactiae* is known to infect farmed fish populations [10], and fishmongers have presented with infections of *Streptococcus iniae* related to fish handling [11]. Fish consumption is generally recommended as healthful due to its DHA and EPA content despite the exposure to heavy metals and other contaminants that may be detrimental to health [12]. For instance, greater fish consumption is associated with child development outcomes including decreased risk of asthma and improved cognition [13, 14]. The decrease in OTUs classified to the species *Clostridium neonatale* with increased maternal fish and seafood intake in infants born by Cesarean section is a possibly beneficial change to infant intestinal microbial communities. We observed consistent associations between maternal DHA and EPA intake and infant gut microbiota. The associations of other maternal dietary factors with specific infant gut microbes were also somewhat unexpected, namely for red and processed meat and for fruit consumption. While fruit is considered healthful and red and processed meat consumption is recommended to be limited, OTUs in the genus *Bifidobacterium*, generally recognized as a beneficial microbe, were decreased with increasing maternal fruit consumption in vaginally born infants yet increased with higher maternal red and processed meat consumption in those born by Cesarean section. The results from infant gut microbiome cluster analyses are consistent with this observation. For example, it is surprising that higher maternal fruit intake is associated with an increased probability of an infant delivered vaginally belonging to cluster 2, in which the genera *Bifidobacterium* and *Bacteroides* are underrepresented and the family *Clostridiaceae* is overrepresented compared to the other two clusters.

The effect of maternal dairy intake on infant gut microbiota was primarily observed in infants delivered by Cesarean section. Infants delivered by Cesarean section are at increased risk of dairy allergies compared to those born vaginally [15, 16] and show decreased colonization of milk-digesting bacteria [7, 17] including the genus *Lactobacillus*. One recent study suggests that both the microbial and fatty acid composition of breast milk may differ by delivery mode [18], so the differential effect of maternal dairy intake by delivery mode on the infant gut microbiome may be attributable to the differences in breast milk microbiota or lipids in relation to maternal diet by delivery mode. Our observation of increased colonization with the species *Acinetobacter rhizosphaerae*, a microbe that colonizes and promotes growth in plants [19], with maternal dairy in the Cesarean group is interesting in conjunction with the positive associations found between maternal dairy and the species *Clostridium neonatale* and *Staphylococcus* genus in vaginally delivered infants. Species of the genus *Acinetobacter* are increasingly involved in nosocomial infections [20], while the species *Clostridium neonatale* was initially isolated from infants affected by necrotizing enterocolitis [21], and the pathogenic potential of species in the genus *Staphylococcus* is well-established [22, 23]. Thus, it will be important to investigate whether maternal dairy consumption fosters the growth of potentially pathogenic microbes in the infant gut, particularly for infants delivered operatively.

In several cases, a maternal dietary factor was associated with a microbe in both delivery groups but in opposite directions. Since Cesarean delivery results in differential microbial community structure of the infant gut [24], and the gut microbiome is a complex system, such differences are plausible; experimental studies will be required to confirm this observation. However, the associations between maternal diet and infant gut microbial communities did not always differ by delivery mode. For example, the maternal aMED score was consistently positively and negatively associated with the *Enterobacteriaceae* family, and additionally negatively associated with the species *Escherichia coli*, in both delivery mode strata.

Our study sample was drawn from Northern New England, which has a relatively homogenous population; this could limit the generalizability of the study. However, by limiting unmeasured confounding due to differences in human microbiomes between populations that may result in erroneous associations with dietary differences, our study also may have greater internal validity. Maternal diet was assessed by self-report between 24 and 28 weeks of gestation, and it is possible that dietary patterns change near the end of pregnancy or during lactation. However, diets are often stable, and a mid-pregnancy food frequency questionnaire (FFQ)-assessment is likely to be sufficiently representative of late pregnancy and lactation diet in a high proportion of individuals [25, 26]. Nonetheless, the effects we observe may be due in part to maternal diet during lactation. Further, we cannot conclude if the effects of maternal diet occur only in breastfed babies, or also in those mostly fed formula, or a combination of formula and breast milk. It is also possible that associations between maternal exposures and infant microbiota at 6 weeks of life are influenced by factors not investigated in this study.

Future studies examining the relationship between maternal diet and components of breast milk including microbial and nutritional profiles, as well as the potential influence of maternal diet on the fetal microbiome, may offer insight into the mechanisms by which maternal diet influences the infant gut microbiome. Determining the impact of changes in the gut microbiome of infants due to maternal diet on infant health and development is an opportunity to refine dietary recommendations for pregnant and lactating women to support infant health.

## Conclusions

In conclusion, we identified three clusters of infant gut microbial communities and observed effects of multiple maternal prenatal dietary factors on the infant gut microbiome that often varied by delivery mode.

## Methods

### Study population

Subjects for the present study were from the New Hampshire Birth Cohort Study (NHBCS) who provided infant stool samples at 6-week postpartum. As described previously [27, 28], eligible participants for the NHBCS are pregnant women obtaining prenatal care in New Hampshire, US clinics, who reported relying on a private, unregulated well as their home water source. Participants between 18 and 45 years old were recruited between 24 and 28 weeks of gestation. The Center for the Protection of Human Subjects at Dartmouth provided institutional review board approval. All methods were carried out in accordance with the guidelines. Written informed consent was obtained for participation from all subjects for themselves and their children. Between 31 March 2011 when the first potential subject with the opportunity to collect a 6-week infant stool sample from was screened for eligibility and 30 June 2015 when the data were frozen for this analysis, 6569 women were screened for eligibility and 1334 were eligible. Nine hundred seventy-six subjects were enrolled and 361 provided 6-week infant stool samples, 187 of which had 16S rRNA gene sequencing data available (Additional file 1: Figure S2).

Telephone interviews conducted every 4 months ascertained the infant’s feeding method, including the timing of breastfeeding cessation and the start date of each new formula, as applicable, as well as medication use such as antibiotics. Infants reported to have never been introduced to formula and to be breastfed were considered to be exclusively breastfed, while those who were ever breastfed and ever formula-fed were considered to be fed with a combination of breast milk and formula regardless of whether or not the infant was still breastfed at the time of stool collection. Those who were never breastfed and only received formula were considered exclusively formula-fed. The delivery mode was drawn from maternal delivery records, and maternal pre-pregnancy height, weight, and parity were determined via a self-administered questionnaire upon study entry and used to compute pre-pregnancy BMI.

### Maternal dietary data and Mediterranean diet score

Maternal diet during pregnancy was assessed during gestational weeks 24–28 with a validated food frequency questionnaire (FFQ) [25]. We computed the alternative Mediterranean diet (aMED) score based on FFQ-derived measures of daily food intake, following the method of Tobias et al. 2012 [29], excluding alcohol consumption to adapt the score to a pregnant cohort. Daily consumption of vegetables, fruits, legumes, soy, and nuts, whole grains, fish and seafood, and monounsaturated to saturated fat ratio (MUFA:SFA) above the median is considered healthful and confers 1 point to the aMED score. Red and processed meat intake is considered less healthful, and consumption below the median confers 1 point to the aMED score. This results in possible aMED score values from 0 to 7, with a higher score indicating greater adherence to a Mediterranean dietary pattern. We additionally considered each of those dietary components and maternal dairy, polyunsaturated fatty acids (PUFA), and omega-3 fatty acids eicosapentaenoic acid (EPA) and docosahexaenoic acid (DHA) intakes separately.

### Sample collection and DNA extraction

Infant stool samples were collected at the 6-week maternal postpartum follow-up appointment and were aliquoted and frozen at − 80 °C within 24 h. Following established methods reviewed by Wu et al. [30], we used the Zymo DNA extraction kit (Zymo Research) to extract microbial DNA from thawed samples and quantified the DNA using OD260/280 nanodrop.

### Targeted 16S rRNA gene sequencing

The V4-V5 hypervariable region of the bacterial 16S rRNA gene was sequenced at the Marine Biological Laboratory (MBL) in Woods Hole, MA, using established methods [31, 32]. As described previously [24], 16S rDNA V4-V5 amplicons were generated from purified genomic DNA samples using fusion primers. The use of forward primers containing one of eight five-nucleotide barcodes between the Illumina-specific bridge and sequencing primer regions and the 16S-specific region and a single reverse primer containing 1 of 12 Illumina indices enables 96 samples per lane multiplexing. Amplifications were done in triplicate with one negative control for internal quality control at the MBL. We used qPCR (Kapa Biosystems) to quantify the amplicon pool, and one Illumina MiSeq 500 cycle paired-end run to sequence each pool of 96 libraries. We demultiplexed and divided datasets using Illumina MiSeq Reporter and a custom Python script.

### Microbiome profiling

To identify microbial population structure profiles, we used full-length amplicon sequences for the rRNA gene V4-V5 hypervariable regions. We merged the forward and reverse reads based on sequence overlap [33], removed the primer sequences, and discarded the sequences containing any ambiguous nucleotides. We used vsearch [34] to remove chimeras both de novo and in comparison to the RDP classifier training reference.

### Data processing and statistical analysis

We identified open reference operational taxonomic units (OTUs) using QIIME version 1.9.1 [35] with the UCLUST algorithm and 97% similarity [36]. We used PyNAST alignment [37] with Greengenes core reference [38, 39] to build OTU tables and assign taxonomy [39, 40]. Phylogenetic trees were constructed using the FastTree method [41]. Subsequent analyses were performed using R version 3.2.2 [42]. We computed generalized UniFrac distances between pairs of samples using OTU tables and the midpoint rooted phylogenetic tree. Generalized UniFrac unifies weighted and unweighted UniFrac to detect differences in moderately abundant lineages instead of those exclusively in highly abundant or rare lineages [43, 44]. Of 187 infant stool samples with available sequencing data, we dropped 18 for missing maternal FFQ data, 14 for missing infant feeding method, and 7 for missing maternal BMI. We dropped three samples of low quality, with read counts < 5000, yielding a final sample size of 145 (Additional file 1: Figure S2), with 97 vaginally delivered infants and 48 delivered by Cesarean section.

Primary analyses were of vaginally delivered infants. We tested the contribution of maternal dietary measures (as continuous variables) to the infant microbiome at 6 weeks using the *adonis* function in the R package *vegan* [45] with 10,000 permutations. This is a nonparametric permutational multivariate analysis of variance (PERMANOVA) test that partitions a distance matrix by sources of variation and can accept both categorical and continuous variables. In preliminary analyses of all infants, delivery mode, feeding method, as previously shown in our cohort [24], and parity were significantly related to infant stool microbial community composition at 6 weeks of age. We performed crude and adjusted analyses for each dietary food group variable individually, normalized by maternal calorie consumption, and maternal aMED score and MUFA:SFA ratio in vaginally delivered infants. All dietary variables were modeled as continuous variables. Adjusted analyses included both variables known to be related to the infant gut microbiome by previous studies and variables significantly related to infant gut microbiota in our population to control confounding. Accordingly, we adjusted for feeding method (exclusively breastfed, combination fed, exclusively formula fed), maternal BMI (continuous), parity (continuous), and batch and performed a sensitivity analysis in exclusively breastfed infants. We considered *p* < 0.05 to be significant. To visualize the results, we performed principal coordinates analysis (PCoA) on generalized UniFrac distances using the *cmdscale* function in R [42] and plotted the samples by the first two coordinates, colored by each dietary variable tertile to aid in visualization.

Inspired by the work identifying adult enterotypes by Arumugam et al. [46] and Wu et al. [47], we identified clusters of the infant gut microbiome at 6 weeks of age using the partitioning around medoids (PAM) method of clustering on generalized UniFrac distances. We identified clusters in all infants (*n* = 145) and within infants delivered vaginally (*n* = 97) or by Cesarean section (*n* = 48) for within delivery group analyses. We used the elbow method to determine the optimal number of clusters, which is based on the within-cluster sum of squares. To assess the relationship between each maternal dietary factor and infant gut microbiome cluster membership, we used multinomial logistic regression in the R package *nnet* adjusting all models for the same covariates as in previous analyses. The outcome in each model was the infant gut microbiome cluster membership; cluster 1 was used as the reference in all models.

To more precisely identify which microbial taxa contribute to the observed differences in infant stool microbial community composition by maternal dietary factors, we used a series of linear models to test the relationships between each maternal dietary factor and each OTU. We computed the relative abundance for each OTU by dividing the read count in each cell by the total number of reads in the sample and filtered out OTUs with zero or near-zero variance using the *caret* package in R. This results in 624 OTUs. We used a zero replacement procedure and log-ratio transformation to account for the zero-inflated and compositional nature of the data [48] before running linear models. We adjusted *p* values for multiple comparisons using the Benjamini and Hochberg method to control the false discovery rate [49]. In reporting the results, we use the lowest level of assigned taxonomy available, which is most commonly the genus, but ranges from the family to the species level.

All analyses were analogously performed in the Cesarean section group to assess if an effect of maternal diet on the infant gut microbiome exists in the case of surgical delivery. We were unable to adjust for antibiotic usage due to a paucity of exposure and imprecise data on timing of exposure. For sensitivity, we repeated analyses (1) excluding infants with possible antibiotic exposure (*n*_vaginal_ = 3, *n*_Cesarean_ = 2) and those with missing antibiotic exposure information (*n*_vaginal_ = 3, *n*_Cesarean_ = 2) and (2) excluding all infants delivered before 37 weeks gestation (*n* = 8), reported in the supplemental information. We also report unadjusted estimates for analyses and analyses further adjusted for infant age at sample collection in the supplemental information.

## Additional file

Additional file 1: Contains supplemental tables (Tables S1–S16) and figures (Figures S1–S7). (PDF 3429 kb)

## Abbreviations

aMED: Alternative Mediterranean diet
DHA: Docosahexaenoic acid
EPA: Eicosapentaenoic acid
FDR: False discovery rate
FFQ: Food frequency questionnaire
MBL: Marine Biological Laboratory
MUFA:SFA: Monounsaturated to saturated fat ratio
NHBCS: New Hampshire Birth Cohort Study
OTU: Operational taxonomic unit
PAM: Partitioning around medoids
PCoA: Principal coordinates analysis
PERMANOVA: Permutational multivariate analysis of variance
PUFA: Polyunsaturated fatty acids
