# Supplementary material for: Maternal diet during pregnancy is related with the infant stool microbiome in a delivery mode-dependent manner
Source: Microbiome. 2018 Jul 5;6:109. doi: 10.1186/s40168-018-0490-8 (PMC6033232; doi:10.1186/s40168-018-0490-8)
Supplement: Supplementary file 1 — Contains supplemental tables (Tables S1–S16) and figures (Figures S1–S7). (PDF 3429 kb) [file 40168_2018_490_MOESM1_ESM.pdf]

Table S1. Top 20 Most abundant microbial taxa in 6-week infant stool by cluster

	Relative abundance (%)											
	All infants (n = 145)				Vaginal (n = 97)				Cesarean (n = 48)			
Taxa[1]	Overall	Cluster 1	Cluster 2	Cluster 3	Overall	Cluster 1	Cluster 2	Cluster 3	Overall	Cluster 1	Cluster 2	Cluster 3
F.Enterobacteriaceae	20.0	23.8	26.9	12.1	18.8	21.6	34.9	11.7	22.3	11.3	13.5	35.2
G.Bifidobacterium	18.4	41.5	1.64	14.4	20.2	45.5	1.65	14.9	14.6	38.3	6.13	4.11
G.Bacteroides	10.4	0.869	0.0656	25.0	14.9	1.00	0.164	26.7	1.46	2.34	2.79	0.0497
G.Streptococcus	8.10	7.82	14.0	3.93	6.81	6.48	15.3	3.92	10.7	13.6	3.31	13.4
G.Bacteroides.S.fragilis	5.53	0.695	0.739	12.6	7.68	1.16	1.76	12.9	1.18	0.826	0.0107	2.15
G.Enterococcus	3.52	3.38	6.09	1.70	2.76	3.40	5.91	1.32	5.05	5.30	2.48	6.48
F.Erysipelotrichaceae	3.45	1.43	5.09	3.67	3.46	1.49	5.27	3.73	3.44	0.801	6.29	3.43
G.Clostridium.S.neonatale	3.27	2.46	6.78	1.22	2.25	2.28	4.68	1.36	5.33	2.60	13.3	2.24
F.Clostridiaceae	2.50	0.422	7.44	0.286	0.408	0.194	1.04	0.282	6.72	0.644	20.4	2.31
G..Ruminococcus..S.gnavus	2.42	2.27	1.05	3.55	2.99	3.11	2.56	3.10	1.26	1.25	0.00611	2.05
G.Clostridium	2.27	0.127	7.30	0.0468	1.88	0.0189	9.44	0.0519	3.06	0.262	5.82	3.21
F.Lachnospiraceae	1.83	0.769	2.97	1.73	0.893	0.212	0.0324	1.52	3.73	2.41	1.08	6.24
G.Veillonella.S.dispar	1.76	0.955	3.74	0.868	1.58	0.982	4.66	0.760	2.13	1.39	1.80	2.83
G.Lactobacillus	1.67	2.63	1.30	1.26	1.50	1.75	1.87	1.25	2.00	4.23	1.20	1.01
G.Blautia	1.55	1.25	2.06	1.40	1.17	0.482	2.02	1.19	2.33	3.16	2.39	1.75
G.Bacteroides.S.uniformis	1.39	0.618	0.109	2.89	2.04	0.868	0.455	3.16	0.0719	0.107	0.120	0.0191
G.Staphylococcus	1.32	2.45	1.08	0.701	1.26	2.70	0.745	0.768	1.45	0.824	0.586	2.41
G.Bacteroides.S.ovatus	1.20	0.449	0.00928	2.63	1.60	0.0123	0.00477	2.93	0.394	1.32	0.00246	0.0180
G.Bacteroides.S.caccae	0.809	0.0761	0.118	1.85	0.512	0.123	0.275	0.781	1.41	2.05	3.00	0.00274
G..Ruminococcus.	0.779	0.0198	2.43	0.0923	0.519	0.00672	2.36	0.100	1.31	0.0460	0.0161	2.94

[1]OTUs with the same taxonomic assignment were combined to get relative abundances of taxa

[2]F., G., and S. indicate the level of taxonomy to be family, genus, or species respectively

[3]Clusters were identified for all infants, those delivered vaginally, and those delivered by cesarean section for only the infants in those groups

Figure S1. Infant stool microbial taxa relative abundances grouped by cluster.

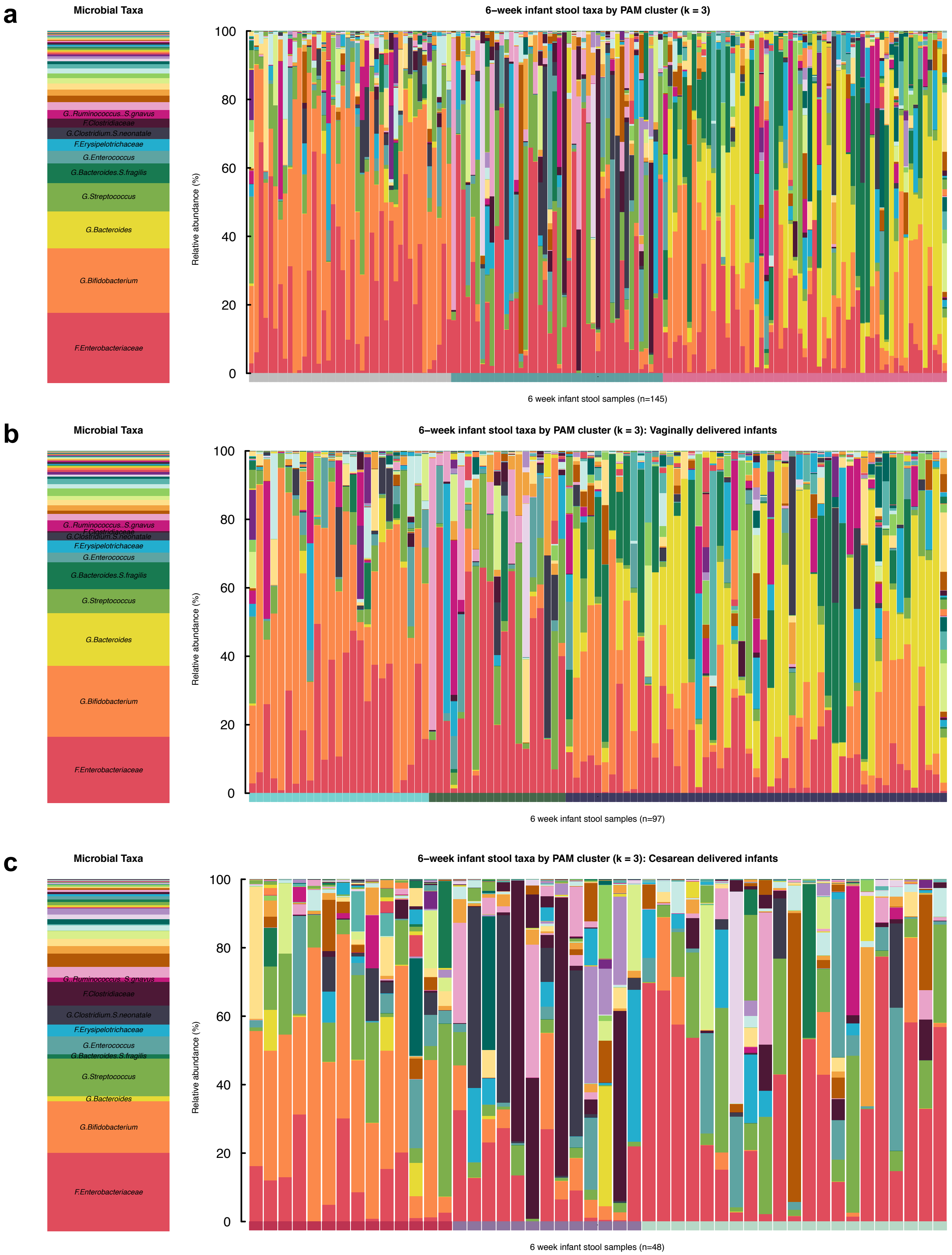

Columns represent subjects and the height of stacked bars indicates the relative abundance of each taxon. OTUs with the same taxonomic assignment were combined. F., G., and S. indicate the level of taxonomy to be family, genus, or species respectively. Subjects are grouped by within-group cluster membership for (a) all subjects (n = 145), (b) infants delivered vaginally (n = 97), and (c) infants delivered by Cesarean section (n = 48).

Table S2a. Relation of maternal aMED score with infant stool microbial OTUs, in infants delivered vaginally (n = 97)

Positive Associations				Negative Associations			
**OTU**	**Taxonomy*** **	**Coefficient**	**p-value**	**OTU**	**Taxonomy*** **	**Coefficient**	**p-value**
228556	*F.Enterobacteriaceae*	2.04	1.5E-03	4447072	*G.Bacteroides.S.uniformis*	-1.37	5.4E-03
920226	*G.Streptococcus*	1.58	2.1E-03	197072	*G.Bacteroides.S.uniformis*	-1.41	0.011
92535	*G.Streptococcus*	1.77	3.7E-03	562376	*G.Dorea*	-1.47	0.013
4318990	*F.Enterobacteriaceae*	1.45	4.0E-03	1109247	*F.Enterobacteriaceae*	-1.75	0.014
NCROTU2526	*F.Enterobacteriaceae*	1.18	4.2E-03	114510	*F.Enterobacteriaceae*	-1.71	0.015
1063759	*G.Corynebacterium*	1.57	5.2E-03	523589	*G.Clostridium.S.neonatale*	-1.79	0.015
4328189	*F.Enterobacteriaceae*	1.24	7.6E-03	797229	*F.Enterobacteriaceae*	-0.87	0.015
3908638	*F.Enterobacteriaceae*	1.79	9.4E-03	231787	*F.Enterobacteriaceae*	-1.71	0.016
737912	*F.Enterobacteriaceae*	1.31	0.010	NROTU7	*G.Coprococcus*	-1.02	0.016
681779	*F.Enterobacteriaceae*	1.37	0.012	289709	*G.Escherichia.S.coli*	-1.65	0.018
688934	*F.Enterobacteriaceae*	1.67	0.012	1111294	*G.Escherichia.S.coli*	-1.71	0.019
821080	*F.Enterobacteriaceae*	1.74	0.013	4310208	*G.Veillonella*	-1.12	0.019
888300	*G.Streptococcus*	1.33	0.013	344154	*G.Bacteroides.S.uniformis*	-1.23	0.024
NROTU27	*F.Enterobacteriaceae*	0.99	0.014	NCROTU2904	*G.Streptococcus*	-0.85	0.024
801438	*F.Enterobacteriaceae*	1.60	0.015	531722	*G.Bacteroides.S.ovatus*	-1.04	0.024
152859	*F.Enterobacteriaceae*	1.26	0.015	975306	*G.Roseburia.S.faecis*	-1.30	0.025
233220	*F.Enterobacteriaceae*	1.68	0.017	356760	*F.Erysipelotrichaceae*	-1.52	0.026
746679	*F.Enterobacteriaceae*	1.57	0.018	588216	*F.Enterobacteriaceae*	-1.53	0.027
315982	*F.Clostridiaceae*	1.02	0.018	3171486	*F.Enterobacteriaceae*	-1.47	0.027
345540	*F.Enterobacteriaceae*	1.31	0.019	141145	*F.Enterobacteriaceae*	-1.53	0.031
1123414	*F.Enterobacteriaceae*	1.42	0.021	3376513	*G.[Ruminococcus].S.gnavus*	-1.11	0.033
1116674	*F.Enterobacteriaceae*	1.29	0.021	217734	*G.Streptococcus.S.anginosus*	-1.28	0.033
322798	*F.Clostridiaceae*	1.10	0.022	3483793	*F.Enterobacteriaceae*	-1.49	0.035
668514	*F.Enterobacteriaceae*	1.52	0.023	4426874	*G.[Ruminococcus].S.gnavus*	-1.05	0.037
241415	*F.Enterobacteriaceae*	1.55	0.025	2283111	*G.Bacteroides.S.uniformis*	-1.13	0.037
819999	*F.Enterobacteriaceae*	1.58	0.028	589071	*G.Bacteroides.S.uniformis*	-1.40	0.039
4376230	*F.Enterobacteriaceae*	1.55	0.029	4385577	*F.Lachnospiraceae*	-1.24	0.040
686972	*F.Enterobacteriaceae*	1.48	0.030	1028632	*G.Escherichia.S.coli*	-1.39	0.041
236821	*F.Enterobacteriaceae*	1.50	0.030	4452632	*G.Clostridium.S.butyricum*	-0.99	0.042
1083508	*F.Xanthomonadaceae*	0.99	0.031	336012	*G.Bacteroides.S.uniformis*	-0.94	0.044
582691	*F.Clostridiaceae*	0.99	0.031	1839271	*G.[Ruminococcus].S.gnavus*	-1.15	0.045
258785	*F.Enterobacteriaceae*	1.01	0.032	331575	*G.[Ruminococcus].S.gnavus*	-1.03	0.046
2529285	*F.Enterobacteriaceae*	1.43	0.042	646549	*G.Pseudomonas*	-0.89	0.051
3506872	*G.Veillonella.S.dispar*	1.10	0.046	1551841	*G.[Ruminococcus].S.gnavus*	-1.06	0.051
579608	*G.Streptococcus*	1.07	0.051	2683271	*G.[Ruminococcus].S.gnavus*	-1.10	0.054
425721	*F.Enterobacteriaceae*	1.08	0.051	320395	*G.Bacteroides.S.uniformis*	-1.09	0.055
813457	*F.Enterobacteriaceae*	0.97	0.051	362997	*G.Bacteroides*	-1.00	0.058
780650	*F.Clostridiaceae*	1.33	0.054	NCROTU4975	*G.Bacteroides.S.uniformis*	-1.16	0.059
396697	*G.Clostridium*	1.10	0.055	3531225	*F.Enterobacteriaceae*	-1.32	0.061
232696	*F.Enterobacteriaceae*	1.19	0.058	1033018	*G.Janthinobacterium.S.lividum*	-0.75	0.062
15366	*G.Streptococcus*	1.23	0.058	503315	*G.Finegoldia*	-1.27	0.063
337909	*G.Clostridium*	0.91	0.060	1654474	*G.[Ruminococcus].S.gnavus*	-1.07	0.070
759061	*F.Enterobacteriaceae*	1.22	0.063	176704	*G.[Ruminococcus].S.gnavus*	-0.95	0.071
972033	*G.Streptococcus*	1.12	0.068	332588	*G.Bacteroides.S.uniformis*	-0.87	0.074
355471	*F.Clostridiaceae*	0.87	0.071	288442	*G.[Ruminococcus].S.gnavus*	-1.05	0.077
903426	*G.Rothia.S.mucilaginosa*	1.33	0.074	359954	*G.Veillonella*	-0.71	0.079
518002	*F.Enterobacteriaceae*	1.19	0.076	NROTU36	*F.Lachnospiraceae*	-0.78	0.080
988542	*G.Haemophilus.S.parainfluenzae*	1.22	0.077	254938	*F.Oxalobacteraceae*	-0.63	0.085
703635	*F.Enterobacteriaceae*	0.87	0.077	1108656	*F.Enterobacteriaceae*	-1.21	0.090
15431	*G.Streptococcus*	1.17	0.080	328617	*G.Bacteroides.S.uniformis*	-0.78	0.090
4349891	*G.Lactobacillus*	0.75	0.083	2876801	*G.Bacteroides.S.uniformis*	-0.98	0.093
210269	*F.Enterobacteriaceae*	1.23	0.085	352304	*F.Lachnospiraceae*	-0.99	0.094
754778	*F.Enterobacteriaceae*	1.15	0.085	182517	*G.[Ruminococcus].S.gnavus*	-0.99	0.094
203579	*F.Enterobacteriaceae*	1.12	0.085	4333897	*F.Enterobacteriaceae*	-1.18	0.095
3228974	*F.Enterobacteriaceae*	0.73	0.086	299267	*F.Enterobacteriaceae*	-1.12	0.096
NCROTU586	*F.Enterobacteriaceae*	0.76	0.089	577710	*G.Blautia.S.producta*	-0.75	0.096
1119540	*F.Enterobacteriaceae*	1.19	0.091	4371046	*G.Bacteroides.S.uniformis*	-1.00	0.096
776980	*F.Enterobacteriaceae*	1.23	0.091	271214	*G.Bacteroides*	-1.22	0.096
261241	*G.Enterococcus*	0.76	0.092				
17309	*G.Lactobacillus*	0.86	0.095				
466445	*F.Enterobacteriaceae*	0.74	0.096				
794205	*G.Lactobacillus*	0.77	0.099				

*NROTU = New.ReferenceOTU

**NCROTU = New.CleanUp.ReferenceOTU

***O., *F.*, *G.*, and *S.* in taxonomy labels indicate that the level of taxonomy is order, family, genus, or species.

Table S2b. Relation of maternal dairy intake with infant stool microbial OTUs, in infants delivered vaginally (n = 97)

Positive Associations				Negative Associations			
**OTU**	**Taxonomy*** **	**Coefficient**	**p-value**	**OTU**	**Taxonomy*** **	**Coefficient**	**p-value**
523589	*G.Clostridium.S.neonatale*	2.80	2.2E-03	NCROTU3325	*F.Enterobacteriaceae*	-1.54	7.7E-03
2202350	*G.Staphylococcus*	1.50	4.4E-03	309720	*F.Ruminococcaceae*	-1.41	0.027
806179	*G.Lactobacillus*	1.45	0.013	NROTU20	*F.Lachnospiraceae*	-1.21	0.040
NCROTU2904	*G.Streptococcus*	1.08	0.022	4401450	*F.Enterobacteriaceae*	-1.23	0.058
370183	*G.Blautia*	1.65	0.024	NROTU25	*F.Lachnospiraceae*	-1.15	0.059
NCROTU4270	*G.Clostridium.S.neonatale*	1.13	0.024	851323	*G.Parabacteroides*	-1.82	0.062
503406	*G.Peptoniphilus*	1.77	0.027	364034	*F.Lachnospiraceae*	-1.46	0.071
503315	*G.Finegoldia*	1.88	0.028	563654	*G.Lactobacillus*	-0.99	0.075
299267	*F.Enterobacteriaceae*	1.79	0.034	316675	*F.Peptostreptococcaceae*	-1.24	0.091
563086	*G.[Ruminococcus]*	1.14	0.036	92535	*G.Streptococcus*	-1.32	0.091
1108960	*G.Sphingomonas*	1.15	0.038	4472685	*G.Streptococcus*	-1.32	0.094
NCROTU3131	*G.Streptococcus*	1.16	0.044	322798	*F.Clostridiaceae*	-1.02	0.095
176775	*G.Phascolarctobacterium*	1.45	0.054	526583	*F.Clostridiaceae*	-1.03	0.100
359175	*F.Ruminococcaceae*	1.20	0.059	572843	G.Enterococcus	-1.40	0.101
4310208	*G.Veillonella*	1.13	0.064	360238	*F.Erysipelotrichaceae*	-1.25	0.106
1084906	*G.Staphylococcus*	0.49	0.069	342666	*F.Clostridiaceae*	-0.79	0.109
984924	*G.Staphylococcus*	1.21	0.070	682726	*G.Eggerthella.S.lenta*	-1.48	0.116
217734	*G.Streptococcus.S.anginosus*	1.38	0.070	1649772	*G.Escherichia.S.coli*	-1.11	0.117
1047077	*G.Actinomyces*	1.24	0.074	4376230	*F.Enterobacteriaceae*	-1.40	0.122
4376828	*G.Bifidobacterium*	1.19	0.081	876714	*G.Pseudomonas*	-1.05	0.123
2250983	*G.Clostridium.S.neonatale*	0.97	0.085	628226	*F.Peptostreptococcaceae*	-0.77	0.135
181239	*G.Bacteroides.S.uniformis*	0.97	0.090	NCROTU1450	*F.Clostridiaceae*	-0.62	0.138
183480	*G.Bacteroides*	1.07	0.098	355471	*F.Clostridiaceae*	-0.88	0.147
137609	*G.Clostridium.S.neonatale*	0.96	0.100	712677	*O.Clostridiales*	-0.99	0.149
4452632	*G.Clostridium.S.butyricum*	1.01	0.102	198423	*G.[Ruminococcus].S.gnavus*	-1.30	0.149
4308688	*G.Bifidobacterium*	1.02	0.103				
NROTU7	*G.Coprococcus*	0.88	0.104				
114821	*G.Veillonella*	1.45	0.113				
1104963	*F.Clostridiaceae*	1.08	0.114				
813944	*G.Lactobacillus*	1.09	0.118				
4294457	*G.Rothia.S.mucilaginosa*	1.25	0.122				
814442	*F.Enterobacteriaceae*	0.97	0.124				
1995363	*G.Staphylococcus.S.aureus*	0.97	0.128				
581474	*G.Lactobacillus*	0.91	0.132				
541328	*G.Clostridium.S.neonatale*	1.27	0.132				
238205	*G.Clostridium.S.neonatale*	0.77	0.133				
4413347	*G.Bifidobacterium*	1.02	0.136				
879972	*G.Streptococcus*	0.80	0.138				
4278525	*G.Bacteroides*	1.03	0.139				
259993	*G.Lactobacillus*	0.89	0.141				
1075821	*G.Alloiococcus*	1.31	0.147				
NCROTU2292	*F.Clostridiaceae*	1.12	0.147				
231787	*F.Enterobacteriaceae*	1.31	0.149				
614083	*G.Staphylococcus*	1.12	0.150				

*NROTU = New.ReferenceOTU

**NCROTU = New.CleanUp.ReferenceOTU

***O., *F.*, *G.*, and *S.* in taxonomy labels indicate that the level of taxonomy is order, family, genus, or species.

Table S2c. Relation of maternal fish and seafood intake with infant stool microbial OTUs, in infants delivered vaginally (n = 97)

Positive Associations				Negative Associations			
**OTU**	**Taxonomy*** **	**Coefficient**	**p-value**	**OTU**	**Taxonomy*** **	**Coefficient**	**p-value**
92535	*G.Streptococcus*	21.99	1.8E-03	797229	*F.Enterobacteriaceae*	-11.28	6.3E-03
1098340	*G.Streptococcus*	18.32	3.0E-03	344154	*G.Bacteroides.S.uniformis*	-16.71	7.6E-03
1027587	*G.Streptococcus*	16.08	3.5E-03	2876801	*G.Bacteroides.S.uniformis*	-16.21	0.016
754778	*F.Enterobacteriaceae*	21.95	4.3E-03	336012	*G.Bacteroides.S.uniformis*	-12.50	0.020
875735	*G.Actinomyces*	16.55	5.2E-03	4447072	*G.Bacteroides.S.uniformis*	-13.27	0.022
425721	*F.Enterobacteriaceae*	17.28	6.7E-03	197072	*G.Bacteroides.S.uniformis*	-14.86	0.022
821080	*F.Enterobacteriaceae*	21.64	7.6E-03	326662	*G.Bacteroides.S.uniformis*	-13.83	0.028
236821	*F.Enterobacteriaceae*	20.92	9.0E-03	364179	*G.Bacteroides.S.caccae*	-15.11	0.032
518002	*F.Enterobacteriaceae*	19.63	0.011	2283111	*G.Bacteroides.S.uniformis*	-13.39	0.033
210269	*F.Enterobacteriaceae*	20.30	0.013	4371046	*G.Bacteroides.S.uniformis*	-14.74	0.034
4376230	*F.Enterobacteriaceae*	20.33	0.014	348027	*G.Bacteroides.S.uniformis*	-12.52	0.034
4328189	*F.Enterobacteriaceae*	12.80	0.018	320395	*G.Bacteroides.S.uniformis*	-13.64	0.038
1101669	*F.Gemellaceae*	18.82	0.018	589071	*G.Bacteroides.S.uniformis*	-15.38	0.052
274754	*F.Enterobacteriaceae*	16.76	0.020	369555	*G.Ruminococcus*	-10.23	0.052
233220	*F.Enterobacteriaceae*	18.95	0.021	332588	*G.Bacteroides.S.uniformis*	-10.93	0.053
963779	*G.Agrobacterium*	12.21	0.028	350277	*G.Bacteroides.S.uniformis*	-12.99	0.056
119010	*F.Enterobacteriaceae*	15.81	0.028	4420408	*G.Bacteroides*	-13.17	0.056
688934	*F.Enterobacteriaceae*	16.94	0.030	465079	*G.Staphylococcus.S.aureus*	-8.39	0.063
759061	*F.Enterobacteriaceae*	16.32	0.031	231787	*F.Enterobacteriaceae*	-15.26	0.067
2529285	*F.Enterobacteriaceae*	17.47	0.032	NCROTU2904	*G.Streptococcus*	-7.99	0.068
4305815	*G.Streptococcus*	11.93	0.033	1109247	*F.Enterobacteriaceae*	-14.64	0.077
668514	*F.Enterobacteriaceae*	16.40	0.035	304641	*G.Escherichia.S.coli*	-12.34	0.078
817734	*G.Pseudomonas*	11.10	0.036	503315	*G.Finegoldia*	-13.95	0.078
152859	*F.Enterobacteriaceae*	12.58	0.037	181239	*G.Bacteroides.S.uniformis*	-9.26	0.079
4416562	*F.Enterobacteriaceae*	16.31	0.038	295411	*F.Clostridiaceae*	-7.41	0.087
819999	*F.Enterobacteriaceae*	17.25	0.039	852030	*G.Staphylococcus*	-8.59	0.092
776980	*F.Enterobacteriaceae*	17.34	0.039	1052663	*G.Staphylococcus*	-9.60	0.094
466445	*F.Enterobacteriaceae*	10.59	0.039	592160	*G.Lactobacillus*	-14.44	0.099
686972	*F.Enterobacteriaceae*	16.25	0.041				
1083508	*F.Xanthomonadaceae*	10.82	0.043				
3908638	*F.Enterobacteriaceae*	16.21	0.044				
232696	*F.Enterobacteriaceae*	14.50	0.046				
4290143	*G.Streptococcus*	11.99	0.046				
794205	*G.Lactobacillus*	10.72	0.047				
NROTU27	*F.Enterobacteriaceae*	9.32	0.048				
192342	*F.Enterobacteriaceae*	13.41	0.048				
NCROTU2526	*F.Enterobacteriaceae*	9.12	0.061				
149034	*F.Enterobacteriaceae*	11.16	0.062				
241415	*F.Enterobacteriaceae*	14.79	0.068				
342397	*G.[Ruminococcus].S.gnavus*	11.65	0.069				
243185	*F.Enterobacteriaceae*	14.65	0.073				
743120	*F.Enterobacteriaceae*	9.42	0.073				
1110763	*F.Enterobacteriaceae*	14.07	0.074				
861807	*G.Corynebacterium*	13.78	0.074				
164789	*F.Enterobacteriaceae*	9.64	0.074				
1104936	*F.Enterobacteriaceae*	14.62	0.075				
511795	*G.Streptococcus.S.anginosus*	9.25	0.076				
1083194	*G.Streptococcus*	13.63	0.078				
1123414	*F.Enterobacteriaceae*	12.73	0.078				
203579	*F.Enterobacteriaceae*	13.22	0.081				
813457	*F.Enterobacteriaceae*	10.02	0.083				
1119540	*F.Enterobacteriaceae*	14.20	0.083				
228556	*F.Enterobacteriaceae*	13.13	0.084				
813217	*F.Enterobacteriaceae*	14.24	0.090				
801438	*F.Enterobacteriaceae*	12.97	0.092				
656517	*F.Enterobacteriaceae*	10.41	0.098				

*NROTU = New.ReferenceOTU

**NCROTU = New.CleanUp.ReferenceOTU

***O., *F.*, *G.*, and *S.* in taxonomy labels indicate that the level of taxonomy is order, family, genus, or species.

Table S2d. Relation of maternal fruit intake with infant stool microbial OTUs, in infants delivered vaginally (n = 97)

Positive Associations				Negative Associations			
**OTU**	**Taxonomy*** **	**Coefficient**	**p-value**	**OTU**	**Taxonomy*** **	**Coefficient**	**p-value**
10085	*F.Enterobacteriaceae*	2.32	5.9E-03	3171486	*F.Enterobacteriaceae*	-3.14	2.8E-03
203579	*F.Enterobacteriaceae*	2.51	0.015	102049	*G.Bifidobacterium*	-2.21	3.1E-03
236821	*F.Enterobacteriaceae*	2.51	0.024	471180	*G.Bifidobacterium*	-2.92	6.9E-03
1625448	*F.Clostridiaceae*	1.14	0.027	4413347	*G.Bifidobacterium*	-2.24	9.1E-03
173744	*G.Megasphaera*	1.65	0.031	1073276	*G.Streptococcus*	-2.06	0.011
589277	*G.Bacteroides*	1.09	0.032	813479	*G.Bifidobacterium*	-2.61	0.013
4478358	*G.Veillonella.S.dispar*	1.89	0.033	292521	*G.Bifidobacterium*	-1.05	0.016
4328189	*F.Enterobacteriaceae*	1.57	0.036	3483793	*F.Enterobacteriaceae*	-2.72	0.016
582691	*F.Clostridiaceae*	1.54	0.037	4312969	*G.Staphylococcus*	-1.49	0.019
239863	*F.Clostridiaceae*	1.05	0.039	696563	*G.Blautia.S.producta*	-2.30	0.020
668514	*F.Enterobacteriaceae*	2.18	0.043	254938	*F.Oxalobacteraceae*	-1.36	0.020
2529285	*F.Enterobacteriaceae*	2.25	0.045	NCROTU2601	*F.Enterobacteriaceae*	-1.28	0.020
988542	*G.Haemophilus.S.parainfluenzae*	2.19	0.047	1017249	*G.Bifidobacterium*	-2.38	0.022
1105343	*F.Ruminococcaceae*	1.31	0.047	NROTU36	*F.Lachnospiraceae*	-1.62	0.023
166896	*F.Clostridiaceae*	1.13	0.051	339532	*G.Bifidobacterium*	-2.30	0.027
NCROTU586	*F.Enterobacteriaceae*	1.37	0.056	553611	*G.Bifidobacterium*	-2.30	0.027
1116674	*F.Enterobacteriaceae*	1.72	0.056	983335	*G.Streptococcus*	-1.59	0.028
295411	*F.Clostridiaceae*	1.12	0.060	577294	*G.Parabacteroides.S.distasonis*	-1.78	0.043
369429	*G.[Ruminococcus]*	1.80	0.063	361702	*G.Ruminococcus*	-1.70	0.044
1119540	*F.Enterobacteriaceae*	2.09	0.064	484304	*G.Bifidobacterium*	-2.15	0.045
NROTU27	*F.Enterobacteriaceae*	1.20	0.065	365484	*O.Clostridiales*	-1.63	0.049
303379	*F.Clostridiaceae*	0.82	0.065	489671	*G.Staphylococcus*	-1.83	0.050
315982	*F.Clostridiaceae*	1.28	0.066	1142029	*G.Bifidobacterium*	-2.26	0.058
NCROTU2526	*F.Enterobacteriaceae*	1.23	0.068	132041	*G.Bifidobacterium*	-1.58	0.059
NCROTU3787	*F.Clostridiaceae*	0.75	0.072	797229	*F.Enterobacteriaceae*	-1.07	0.062
894969	*G.Streptococcus*	1.27	0.076	4303016	*G.Streptococcus*	-2.05	0.062
3908638	*F.Enterobacteriaceae*	1.96	0.078	541299	*G.Phenylobacterium*	-1.24	0.063
801438	*F.Enterobacteriaceae*	1.84	0.083	997439	*G.Bifidobacterium*	-1.80	0.065
331697	*F.Enterobacteriaceae*	1.78	0.084	289709	*G.Escherichia.S.coli*	-2.06	0.067
681779	*F.Enterobacteriaceae*	1.53	0.084	524725	*G.Atopobium*	-2.06	0.067
355471	*F.Clostridiaceae*	1.28	0.098	555945	*F.Peptostreptococcaceae*	-1.37	0.068
171518	*F.Enterobacteriaceae*	1.49	0.098	503315	*G.Finegoldia*	-1.96	0.073
				4376828	*G.Bifidobacterium*	-1.55	0.074
				2202350	*G.Staphylococcus*	-1.21	0.075
				840914	*G.Prevotella.S.copri*	-1.52	0.088
				589071	*G.Bacteroides.S.uniformis*	-1.86	0.089
				369555	*G.Ruminococcus*	-1.24	0.090
				511795	*G.Streptococcus.S.anginosus*	-1.19	0.099

*NROTU = New.ReferenceOTU

**NCROTU = New.CleanUp.ReferenceOTU

***O., *F.*, *G.*, and *S.* in taxonomy labels indicate that the level of taxonomy is order, family, genus, or species.

Table S2e. Relation of maternal red and processed meat intake with infant stool microbial OTUs, in infants delivered vaginally (n = 97)

Positive Associations				Negative Associations			
**OTU**	**Taxonomy*****	**Coefficient**	**p-value**	**OTU**	**Taxonomy*****	**Coefficient**	**p-value**
342666	*F.Clostridiaceae*	4.34	0.013	4440670	*G.Veillonella*	-6.03	7.9E-03
589071	*G.Bacteroides.S.uniformis*	6.99	0.022	4472685	*G.Streptococcus*	-7.07	0.011
351231	*G.Bacteroides.S.fragilis*	5.42	0.023	579608	*G.Streptococcus*	-5.67	0.020
164413	*G.Enterococcus*	3.31	0.031	NROTU23	*F.Lachnospiraceae*	-7.09	0.024
NROTU25	*F.Lachnospiraceae*	4.47	0.039	470382	*G.Coprococcus*	-4.41	0.029
4447072	*G.Bacteroides.S.uniformis*	4.45	0.047	836783	*G.Shewanella*	-3.87	0.030
344154	*G.Bacteroides.S.uniformis*	4.73	0.053	515869	*G.Faecalibacterium.S.prausnitzii*	-6.03	0.031
NCROTU4975	*G.Bacteroides.S.uniformis*	5.17	0.060	516814	*G.Streptococcus*	-3.94	0.033
362539	*F.Lachnospiraceae*	4.79	0.065	320888	*F.Clostridiaceae*	-3.63	0.036
4371046	*G.Bacteroides.S.uniformis*	4.96	0.066	364034	*F.Lachnospiraceae*	-5.91	0.039
362997	*G.Bacteroides*	4.32	0.067	173744	*G.Megasphaera*	-4.29	0.045
332718	*G.Streptococcus*	3.01	0.068	342427	*G.Veillonella.S.dispar*	-4.61	0.047
320395	*G.Bacteroides.S.uniformis*	4.49	0.077	NCROTU3131	*G.Streptococcus*	-4.00	0.050
4457268	*F.Enterobacteriaceae*	4.23	0.079	134265	*G.Prevotella*	-4.09	0.054
354850	*G.Bacteroides*	5.60	0.081	554338	*G.Blautia*	-5.49	0.062
345362	*F.Enterobacteriaceae*	5.26	0.086	12574	*G.Actinomyces*	-6.01	0.067
4060124	*G.Bacteroides*	5.02	0.092	328458	*G.Streptococcus*	-3.46	0.069
197072	*G.Bacteroides.S.uniformis*	4.22	0.095	332732	*G.Bacteroides*	-3.92	0.069
1108656	*F.Enterobacteriaceae*	5.26	0.102	364029	*G.Bacteroides*	-3.82	0.072
3472078	*G.Bacteroides.S.fragilis*	4.84	0.105	903426	*G.Rothia.S.mucilaginosa*	-5.93	0.076
4479397	*G.Bacteroides.S.fragilis*	4.10	0.114	17309	*G.Lactobacillus*	-4.11	0.076
972033	*G.Streptococcus*	4.26	0.123	239863	*F.Clostridiaceae*	-2.53	0.078
668514	*F.Enterobacteriaceae*	4.58	0.130	177150	*G.Bacteroides*	-3.93	0.080
339599	*G.Bacteroides.S.fragilis*	4.12	0.132	NROTU11	*F.Enterobacteriaceae*	-2.59	0.081
349024	*G.Streptococcus*	4.56	0.133	316675	*F.Peptostreptococcaceae*	-4.52	0.084
323231	*G.Bacteroides*	4.85	0.134	941096	*G.Streptococcus*	-4.49	0.086
3887769	*G.Bacteroides*	4.52	0.140	514272	*G.Coprococcus*	-4.82	0.086
563654	*G.Lactobacillus*	2.87	0.147	892845	*G.Enterococcus*	-3.89	0.088
				1108960	*G.Sphingomonas*	-3.33	0.093
				963344	*G.Enhydrobacter*	-3.91	0.095
				189971	*G.Blautia*	-2.97	0.096
				1007926	*G.Streptococcus*	-3.31	0.099
				703741	*G.Lactobacillus*	-3.39	0.100
				1625448	*F.Clostridiaceae*	-2.36	0.105
				1090059	*G.Enterococcus*	-2.76	0.110
				2656868	*G.Bacteroides*	-3.51	0.111
				4333020	*F.Enterobacteriaceae*	-4.36	0.116
				988542	*G.Haemophilus.S.parainfluenzae*	-4.87	0.116
				3228974	*F.Enterobacteriaceae*	-2.99	0.117
				548587	*G.[Eubacterium].S.dolichum*	-4.85	0.122
				529180	*G.Coprococcus*	-3.68	0.137
				2272797	*G.Enterococcus*	-4.18	0.137
				925707	*G.Streptococcus*	-3.43	0.147
				152859	*F.Enterobacteriaceae*	-3.39	0.147

*NROTU = New.ReferenceOTU

**NCROTU = New.CleanUp.ReferenceOTU

***O., *F.*, *G.*, and *S.* in taxonomy labels indicate that the level of taxonomy is order, family, genus, or species.

Table S2f. Relation of maternal MUFA:SFA ratio with infant stool microbial OTUs, in infants delivered vaginally (n = 97)

Positive Associations				Negative Associations			
**OTU**	**Taxonomy*** **	**Coefficient**	**p-value**	**OTU**	**Taxonomy*** **	**Coefficient**	**p-value**
NCROTU1008	*G.Blautia*	7.35	5.1E-03	NCROTU2904	*G.Streptococcus*	-5.99	0.017
628226	*F.Peptostreptococcaceae*	7.42	6.0E-03	103166	*F.Enterobacteriaceae*	-6.06	0.018
92535	*G.Streptococcus*	11.10	7.1E-03	503406	*G.Peptoniphilus*	-10.04	0.019
325977	*G.[Ruminococcus]*	8.28	0.020	532521	*G.Peptostreptococcus.S.anaerobius*	-7.18	0.024
297057	*G.Bacteroides*	6.86	0.020	10085	*F.Enterobacteriaceae*	-7.73	0.030
320888	*F.Clostridiaceae*	6.03	0.020	1726426	*F.Enterobacteriaceae*	-6.03	0.042
920226	*G.Streptococcus*	8.14	0.020	465079	*G.Staphylococcus.S.aureus*	-5.20	0.047
295411	*F.Clostridiaceae*	5.51	0.027	814442	*F.Enterobacteriaceae*	-6.55	0.051
364034	*F.Lachnospiraceae*	9.22	0.032	148620	*F.Enterobacteriaceae*	-6.18	0.053
187035	*G.Blautia*	5.43	0.040	299267	*F.Enterobacteriaceae*	-8.61	0.056
4453060	*G.Enterococcus*	8.79	0.041	4294457	*G.Rothia.S.mucilaginosa*	-8.17	0.059
345448	*F.Clostridiaceae*	5.12	0.041	336012	*G.Bacteroides.S.uniformis*	-5.77	0.066
4480970	*G.Bacteroides.S.caccae*	6.31	0.049	4310208	*G.Veillonella*	-5.97	0.067
572843	*G.Enterococcus*	8.89	0.051	581021	*F.Enterobacteriaceae*	-6.62	0.067
193466	*G.Blautia*	5.34	0.055	114821	*G.Veillonella*	-8.95	0.068
1147925	*F.Clostridiaceae*	5.14	0.055	523589	*G.Clostridium.S.neonatale*	-9.05	0.068
15431	*G.Streptococcus*	8.52	0.058	4473176	*F.Enterobacteriaceae*	-6.40	0.069
302683	*G.Blautia*	5.14	0.062	328617	*G.Bacteroides.S.uniformis*	-5.61	0.072
364926	*G.Bacteroides*	8.80	0.063	1078207	*G.Streptococcus*	-6.75	0.080
NROTU23	*F.Lachnospiraceae*	8.70	0.067	836783	*G.Shewanella*	-4.68	0.082
766768	*G.Enterococcus*	8.70	0.067	171518	*F.Enterobacteriaceae*	-6.56	0.084
1065974	*G.Enterococcus*	8.64	0.068	466445	*F.Enterobacteriaceae*	-4.99	0.096
577170	*G.Bacteroides*	6.37	0.071				
195157	*G.Bacteroides.S.ovatus*	8.91	0.074				
360238	*F.Erysipelotrichaceae*	7.30	0.077				
189971	*G.Blautia*	4.68	0.081				
NROTU20	*F.Lachnospiraceae*	5.52	0.082				
579608	*G.Streptococcus*	6.36	0.085				
1625448	*F.Clostridiaceae*	3.66	0.094				
470382	*G.Coprococcus*	5.11	0.094				

*NROTU = New.ReferenceOTU

**NCROTU = New.CleanUp.ReferenceOTU

***O., *F.*, *G.*, and *S.* in taxonomy labels indicate that the level of taxonomy is order, family, genus, or species.

Table S2g. Relation of maternal DHA intake with infant stool microbial OTUs, in infants delivered vaginally (n = 97)

Positive Associations				Negative Associations			
**OTU**	**Taxonomy*** **	**Coefficient**	**p-value**	**OTU**	**Taxonomy*** **	**Coefficient**	**p-value**
236821	*F.Enterobacteriaceae*	30.93	1.7E-03	369555	*G.Ruminococcus*	-17.85	5.7E-03
819999	*F.Enterobacteriaceae*	31.77	1.8E-03	1111294	*G.Escherichia.S.coli*	-28.52	6.2E-03
92535	*G.Streptococcus*	27.10	1.9E-03	231787	*F.Enterobacteriaceae*	-26.05	0.011
152859	*F.Enterobacteriaceae*	22.74	2.0E-03	114510	*F.Enterobacteriaceae*	-25.15	0.012
688934	*F.Enterobacteriaceae*	29.27	2.1E-03	1109247	*F.Enterobacteriaceae*	-24.71	0.015
210269	*F.Enterobacteriaceae*	30.79	2.3E-03	289709	*G.Escherichia.S.coli*	-23.93	0.017
4462083	*G.Streptococcus*	17.87	2.8E-03	3531225	*F.Enterobacteriaceae*	-23.58	0.020
821080	*F.Enterobacteriaceae*	29.31	3.4E-03	132661	*G.Enterococcus*	-23.14	0.021
801438	*F.Enterobacteriaceae*	27.43	3.4E-03	299267	*F.Enterobacteriaceae*	-21.84	0.023
3908638	*F.Enterobacteriaceae*	28.65	3.7E-03	4294457	*G.Rothia.S.mucilaginosa*	-20.29	0.027
119010	*F.Enterobacteriaceae*	25.52	3.9E-03	782953	*F.Enterobacteriaceae*	-21.77	0.034
2529285	*F.Enterobacteriaceae*	28.74	3.9E-03	521851	*G.Enterococcus*	-20.76	0.036
203579	*F.Enterobacteriaceae*	26.06	4.8E-03	3483793	*F.Enterobacteriaceae*	-21.36	0.036
228556	*F.Enterobacteriaceae*	26.07	4.9E-03	4333897	*F.Enterobacteriaceae*	-21.10	0.038
1119540	*F.Enterobacteriaceae*	28.07	5.0E-03	1107335	*G.Acinetobacter.S.rhizosphaerae*	-14.03	0.038
754778	*F.Enterobacteriaceae*	26.51	5.4E-03	968675	*G.Haemophilus.S.parainfluenzae*	-13.26	0.040
4376230	*F.Enterobacteriaceae*	28.16	5.6E-03	797229	*F.Enterobacteriaceae*	-10.48	0.043
813217	*F.Enterobacteriaceae*	28.23	6.0E-03	581079	*G.Oscillospira*	-21.40	0.044
776980	*F.Enterobacteriaceae*	27.61	7.5E-03	4457268	*F.Enterobacteriaceae*	-15.52	0.044
232696	*F.Enterobacteriaceae*	23.65	7.9E-03	141145	*F.Enterobacteriaceae*	-20.49	0.044
1110763	*F.Enterobacteriaceae*	24.81	0.010	1696853	*G.Enterococcus*	-12.29	0.046
233220	*F.Enterobacteriaceae*	26.04	0.010	369027	*F.Lachnospiraceae*	-13.28	0.049
241415	*F.Enterobacteriaceae*	25.35	0.011	563086	*G.[Ruminococcus]*	-12.22	0.050
518002	*F.Enterobacteriaceae*	24.15	0.011	1108656	*F.Enterobacteriaceae*	-19.30	0.061
759061	*F.Enterobacteriaceae*	23.11	0.014	345362	*F.Enterobacteriaceae*	-18.39	0.061
243185	*F.Enterobacteriaceae*	24.62	0.014	132041	*G.Bifidobacterium*	-13.75	0.068
258785	*F.Enterobacteriaceae*	16.21	0.016	1028632	*G.Escherichia.S.coli*	-17.68	0.071
331697	*F.Enterobacteriaceae*	21.96	0.016	879972	*G.Streptococcus*	-10.87	0.076
511795	*G.Streptococcus.S.anginosus*	15.20	0.018	588216	*F.Enterobacteriaceae*	-17.48	0.079
813457	*F.Enterobacteriaceae*	16.69	0.019	301149	*F.Enterobacteriaceae*	-10.34	0.080
164789	*F.Enterobacteriaceae*	15.55	0.019	304641	*G.Escherichia.S.coli*	-14.83	0.088
963779	*G.Agrobacterium*	15.94	0.020				
861807	*G.Corynebacterium*	22.03	0.020				
425721	*F.Enterobacteriaceae*	18.23	0.022				
NROTU20	*F.Lachnospiraceae*	15.34	0.023				
737912	*F.Enterobacteriaceae*	16.10	0.029				
686972	*F.Enterobacteriaceae*	21.39	0.030				
1104936	*F.Enterobacteriaceae*	21.78	0.032				
274754	*F.Enterobacteriaceae*	19.12	0.032				
969149	*F.Enterobacteriaceae*	16.93	0.032				
1123414	*F.Enterobacteriaceae*	18.91	0.034				
875735	*G.Actinomyces*	15.36	0.038				
656517	*F.Enterobacteriaceae*	15.99	0.039				
4328189	*F.Enterobacteriaceae*	13.81	0.041				
1108275	*G.Comamonas*	17.90	0.053				
917641	*G.Staphylococcus*	17.85	0.055				
1111874	*F.Enterobacteriaceae*	19.69	0.056				
1116674	*F.Enterobacteriaceae*	15.34	0.059				
837283	*G.Serratia*	18.39	0.059				
746679	*F.Enterobacteriaceae*	18.09	0.060				
1726426	*F.Enterobacteriaceae*	11.43	0.072				
2250983	*G.Clostridium.S.neonatale*	11.45	0.076				
1101669	*F.Gemellaceae*	17.03	0.088				
4318990	*F.Enterobacteriaceae*	12.49	0.090				

*NROTU = New.ReferenceOTU

**NCROTU = New.CleanUp.ReferenceOTU

***O., *F.*, *G.*, and *S.* in taxonomy labels indicate that the level of taxonomy is order, family, genus, or species.

Table S2h. Relation of maternal EPA intake with infant stool microbial OTUs, in infants delivered vaginally (n = 97)

Positive Associations				Negative Associations			
OTU	Taxonomy***	Coefficient	p-value	OTU	Taxonomy***	Coefficient	p-value
152859	*F.Enterobacteriaceae*	23.66	3.6E-04	1111294	*G.Escherichia.S.coli*	-25.50	7.2E-03
4462083	*G.Streptococcus*	18.92	4.5E-04	114510	*F.Enterobacteriaceae*	-22.48	0.014
917641	*G.Staphylococcus*	23.74	4.6E-03	369555	*G.Ruminococcus*	-14.16	0.017
236821	*F.Enterobacteriaceae*	23.98	7.9E-03	231787	*F.Enterobacteriaceae*	-21.58	0.021
232696	*F.Enterobacteriaceae*	21.42	8.3E-03	289709	*G.Escherichia.S.coli*	-20.25	0.027
861807	*G.Corynebacterium*	22.35	0.010	968675	*G.Haemophilus.S.parainfluenzae*	-12.78	0.029
688934	*F.Enterobacteriaceae*	22.59	0.010	132041	*G.Bifidobacterium*	-14.66	0.032
203579	*F.Enterobacteriaceae*	21.76	0.010	1107335	*G.Acinetobacter.S.rhizosphaerae*	-13.05	0.034
210269	*F.Enterobacteriaceae*	23.65	0.011	299267	*F.Enterobacteriaceae*	-18.50	0.035
801438	*F.Enterobacteriaceae*	21.56	0.012	3531225	*F.Enterobacteriaceae*	-19.33	0.036
3908638	*F.Enterobacteriaceae*	22.29	0.014	1696853	*G.Enterococcus*	-11.66	0.038
2529285	*F.Enterobacteriaceae*	22.45	0.014	355307	*F.Ruminococcaceae*	-12.64	0.040
258785	*F.Enterobacteriaceae*	14.86	0.015	132661	*G.Enterococcus*	-18.58	0.042
819999	*F.Enterobacteriaceae*	22.69	0.015	1109247	*F.Enterobacteriaceae*	-18.77	0.044
813217	*F.Enterobacteriaceae*	22.49	0.017	581079	*G.Oscillospira*	-19.29	0.046
821080	*F.Enterobacteriaceae*	21.83	0.018	4294457	*G.Rothia.S.mucilaginosa*	-16.39	0.051
813457	*F.Enterobacteriaceae*	15.16	0.019	563086	*G.[Ruminococcus]*	-10.97	0.053
92535	*G.Streptococcus*	18.74	0.020	521851	*G.Enterococcus*	-17.01	0.059
776980	*F.Enterobacteriaceae*	21.75	0.021	141145	*F.Enterobacteriaceae*	-17.47	0.060
228556	*F.Enterobacteriaceae*	19.45	0.022	369027	*F.Lachnospiraceae*	-11.48	0.062
241415	*F.Enterobacteriaceae*	20.82	0.022	3483793	*F.Enterobacteriaceae*	-17.18	0.065
1110763	*F.Enterobacteriaceae*	20.17	0.022	211191	*F.Ruminococcaceae*	-10.91	0.066
4326406	*G.Streptococcus*	13.15	0.023	NROTU25	*F.Lachnospiraceae*	-11.23	0.076
233220	*F.Enterobacteriaceae*	21.02	0.023	512239	*G.Enterococcus*	-15.31	0.087
754778	*F.Enterobacteriaceae*	19.75	0.024	782953	*F.Enterobacteriaceae*	-15.87	0.091
969149	*F.Enterobacteriaceae*	16.17	0.025	4480970	*G.Bacteroides.S.caccae*	-10.58	0.091
274754	*F.Enterobacteriaceae*	18.21	0.025	716006	*G.Lactococcus*	-13.05	0.094
119010	*F.Enterobacteriaceae*	18.13	0.026	345362	*F.Enterobacteriaceae*	-14.95	0.095
1119540	*F.Enterobacteriaceae*	20.36	0.027	191487	*G.Bacteroides.S.caccae*	-10.14	0.099
2250983	*G.Clostridium.S.neonatale*	12.72	0.029				
759061	*F.Enterobacteriaceae*	18.38	0.032				
518002	*F.Enterobacteriaceae*	18.41	0.035				
511795	*G.Streptococcus.S.anginosus*	12.27	0.036				
331697	*F.Enterobacteriaceae*	17.42	0.037				
243185	*F.Enterobacteriaceae*	19.10	0.038				
4376230	*F.Enterobacteriaceae*	19.21	0.040				
1116674	*F.Enterobacteriaceae*	15.08	0.041				
1055132	*G.Staphylococcus*	12.40	0.043				
NROTU20	*F.Lachnospiraceae*	12.40	0.044				
NCROTU3436	*G.Staphylococcus*	17.82	0.045				
1085410	*G.Streptococcus*	10.58	0.050				
837283	*G.Serratia*	17.34	0.050				
686972	*F.Enterobacteriaceae*	17.54	0.051				
414943	*G.Haemophilus*	13.91	0.055				
656517	*F.Enterobacteriaceae*	13.40	0.059				
1039477	*G.Staphylococcus*	16.26	0.063				
4318990	*F.Enterobacteriaceae*	12.42	0.063				
1111874	*F.Enterobacteriaceae*	17.16	0.068				
737912	*F.Enterobacteriaceae*	12.24	0.069				
1726426	*F.Enterobacteriaceae*	10.39	0.073				
1108275	*G.Comamonas*	15.07	0.074				
1104936	*F.Enterobacteriaceae*	16.47	0.076				
4473176	*F.Enterobacteriaceae*	12.05	0.079				
712047	*F.Clostridiaceae*	7.77	0.087				
539107	*F.Enterobacteriaceae*	8.51	0.088				
1068082	*G.Staphylococcus*	14.85	0.092				
164789	*F.Enterobacteriaceae*	10.24	0.093				

*NROTU = New.ReferenceOTU

**NCROTU = New.CleanUp.ReferenceOTU

***O., *F.*, *G.*, and *S.* in taxonomy labels indicate that the level of taxonomy is order, family, genus, or species.

Table S2i. Relation of maternal nut, legume, and soy intake with infant stool microbial OTUs, in infants delivered vaginally (n = 97)

Positive Associations				Negative Associations			
**OTU**	**Taxonomy*** **	**Coefficient**	**p-value**	**OTU**	**Taxonomy*** **	**Coefficient**	**p-value**
365484	*O.Clostridiales*	4.39	6.2E-04	1726426	*F.Enterobacteriaceae*	-2.41	0.031
544493	*F.Oxalobacteraceae*	2.56	3.5E-03	1551841	*G.[Ruminococcus].S.gnavus*	-2.73	0.047
NCROTU2601	*F.Enterobacteriaceae*	2.05	0.019	312140	*G.Bacteroides*	-2.84	0.049
1078587	*G.Blautia*	2.88	0.019	4359220	*G.Veillonella.S.dispar*	-2.04	0.051
NCROTU835	*F.Enterobacteriaceae*	2.41	0.021	331575	*G.[Ruminococcus].S.gnavus*	-2.42	0.065
766768	*G.Enterococcus*	3.97	0.026	4294457	*G.Rothia.S.mucilaginosa*	-2.85	0.081
316675	*F.Peptostreptococcaceae*	3.22	0.029	3376513	*G.[Ruminococcus].S.gnavus*	-2.29	0.083
NCROTU1008	*G.Blautia*	2.16	0.030	364029	*G.Bacteroides*	-2.08	0.084
193466	*G.Blautia*	2.17	0.038	198788	*G.Bacteroides*	-3.04	0.085
1108960	*G.Sphingomonas*	2.30	0.039	587530	*G.[Eubacterium].S.dolichum*	-1.75	0.090
794205	*G.Lactobacillus*	2.38	0.043	878104	*G.Veillonella.S.dispar*	-2.11	0.097
526583	*F.Clostridiaceae*	2.50	0.046	NCROTU586	*F.Enterobacteriaceae*	-1.86	0.102
297057	*G.Bacteroides*	2.23	0.046	2415144	*G.Bacteroides*	-2.25	0.104
572843	*G.Enterococcus*	3.38	0.049	2683271	*G.[Ruminococcus].S.gnavus*	-2.33	0.108
195157	*G.Bacteroides.S.ovatus*	3.65	0.052	NROTU25	*F.Lachnospiraceae*	-1.94	0.115
359538	*G.Bacteroides.S.caccae*	3.62	0.052	539107	*F.Enterobacteriaceae*	-1.53	0.115
579608	*G.Streptococcus*	2.67	0.054	176704	*G.[Ruminococcus].S.gnavus*	-2.06	0.121
17309	*G.Lactobacillus*	2.43	0.063	606927	*F.Peptostreptococcaceae*	-2.41	0.124
364034	*F.Lachnospiraceae*	2.94	0.071	1106617	*G.Limnohabitans*	-2.01	0.127
187035	*G.Blautia*	1.77	0.077	4478358	*G.Veillonella.S.dispar*	-2.16	0.127
1055824	*G.Staphylococcus*	2.08	0.080	1906483	*G.Bacteroides*	-2.84	0.130
3663794	*G.Lactobacillus*	1.87	0.090	757622	*G.Veillonella.S.dispar*	-2.58	0.131
577294	*G.Parabacteroides.S.distasonis*	2.34	0.094	754778	*F.Enterobacteriaceae*	-2.56	0.133
2582263	*F.Enterobacteriaceae*	2.04	0.094	1654474	*G.[Ruminococcus].S.gnavus*	-2.24	0.134
189971	*G.Blautia*	1.66	0.101	1566189	*G.Bacteroides*	-2.16	0.138
4473975	*G.Enterococcus*	1.92	0.103	10085	*F.Enterobacteriaceae*	-1.98	0.143
320888	*F.Clostridiaceae*	1.58	0.108	1809696	*G.Bacteroides*	-2.68	0.144
336559	*G.Bacteroides*	1.74	0.116				
1065974	*G.Enterococcus*	2.77	0.121				
1055132	*G.Staphylococcus*	1.84	0.121				
1696853	*G.Enterococcus*	1.69	0.122				
173654	*F.Enterobacteriaceae*	2.51	0.123				
1029949	*G.Lachnospira*	1.84	0.126				
226338	*G.Enterococcus*	2.49	0.128				
920226	*G.Streptococcus*	2.01	0.132				
291090	*G.Parabacteroides.S.distasonis*	2.79	0.141				

*NROTU = New.ReferenceOTU

**NCROTU = New.CleanUp.ReferenceOTU

***O., *F.*, *G.*, and *S.* in taxonomy labels indicate that the level of taxonomy is order, family, genus, or species.

Table S2j. Relation of maternal PUFA intake with infant stool microbial OTUs, in infants delivered vaginally (n = 97)

Positive Associations				Negative Associations			
**OTU**	**Taxonomy*** **	**Coefficient**	**p-value**	**OTU**	**Taxonomy*** **	**Coefficient**	**p-value**
NCROTU1008	*G.Blautia*	0.77	5.1E-03	364029	*G.Bacteroides*	-0.81	0.014
794205	*G.Lactobacillus*	0.83	0.011	217734	*G.Streptococcus.S.anginosus*	-0.92	0.029
364034	*F.Lachnospiraceae*	1.12	0.012	606927	*F.Peptostreptococcaceae*	-0.93	0.031
92535	*G.Streptococcus*	1.04	0.016	352304	*F.Lachnospiraceae*	-0.88	0.033
15431	*G.Streptococcus*	1.07	0.022	NCROTU2904	*G.Streptococcus*	-0.56	0.035
1110317	*G.Lactobacillus*	1.00	0.041	187623	*G.Bacteroides*	-0.78	0.042
316675	*F.Peptostreptococcaceae*	0.82	0.044	224670	*F.Enterobacteriaceae*	-0.59	0.050
302683	*G.Blautia*	0.58	0.044	1007926	*G.Streptococcus*	-0.58	0.067
187035	*G.Blautia*	0.56	0.045	1105343	*F.Ruminococcaceae*	-0.53	0.068
572843	*G.Enterococcus*	0.94	0.047	3583645	*G.Bacteroides*	-0.75	0.080
359538	*G.Bacteroides.S.caccae*	1.02	0.049	776980	*F.Enterobacteriaceae*	-0.84	0.102
365484	*O.Clostridiales*	0.68	0.064	NROTU38	*G.Ruminococcus*	-0.50	0.103
1078587	*G.Blautia*	0.62	0.069	879972	*G.Streptococcus*	-0.48	0.108
193466	*G.Blautia*	0.51	0.079	238205	*G.Clostridium.S.neonatale*	-0.46	0.111
198145	*G.Blautia*	0.45	0.082	196176	*G.Dorea*	-0.78	0.113
292364	*G.Enterococcus*	0.57	0.087	3531225	*F.Enterobacteriaceae*	-0.78	0.120
1076316	*G.Staphylococcus*	0.75	0.087	1107335	*G.Acinetobacter.S.rhizosphaerae*	-0.51	0.124
102049	*G.Bifidobacterium*	0.56	0.091	304641	*G.Escherichia.S.coli*	-0.64	0.130
189971	*G.Blautia*	0.47	0.091	380567	*G.Corynebacterium*	-0.55	0.139
4413347	*G.Bifidobacterium*	0.63	0.100	4334711	*G.Bacteroides*	-0.54	0.148
NROTU20	*F.Lachnospiraceae*	0.54	0.101				
72820	*G.Bifidobacterium*	0.48	0.102				
NROTU11	*F.Enterobacteriaceae*	0.38	0.104				
285497	*F.Caulobacteraceae*	0.50	0.109				
541299	*G.Phenylobacterium*	0.46	0.117				
4388645	*G.Enterococcus*	0.73	0.118				
2582263	*F.Enterobacteriaceae*	0.51	0.130				
342666	*F.Clostridiaceae*	0.42	0.130				
1064036	*G.Peptoniphilus*	0.69	0.133				
524292	*G.Staphylococcus*	0.61	0.140				
1055132	*G.Staphylococcus*	0.48	0.143				
132041	*G.Bifidobacterium*	0.54	0.143				
505053	*G.Staphylococcus*	0.46	0.144				
134265	*G.Prevotella*	0.49	0.145				
701864	*G.Enterococcus*	0.57	0.150				
1101669	*F.Gemellaceae*	0.70	0.150				

*NROTU = New.ReferenceOTU

**NCROTU = New.CleanUp.ReferenceOTU

***O., *F.*, *G.*, and *S.* in taxonomy labels indicate that the level of taxonomy is order, family, genus, or species.

Table S2k. Relation of maternal vegetable intake with infant stool microbial OTUs, in infants delivered vaginally (n = 97)

Positive Associations				Negative Associations			
**OTU**	**Taxonomy*** **	**Coefficient**	**p-value**	**OTU**	**Taxonomy*** **	**Coefficient**	**p-value**
920226	*G.Streptococcus*	1.43	0.013	362539	*F.Lachnospiraceae*	-1.88	2.9E-03
1067519	*G.Staphylococcus*	1.69	0.021	2876801	*G.Bacteroides.S.uniformis*	-1.57	0.014
92535	*G.Streptococcus*	1.55	0.023	3171486	*F.Enterobacteriaceae*	-1.77	0.016
997439	*G.Bifidobacterium*	1.52	0.023	181239	*G.Bacteroides.S.uniformis*	-1.19	0.017
291090	*G.Parabacteroides.S.distasonis*	1.85	0.025	114510	*F.Enterobacteriaceae*	-1.82	0.019
515869	*G.Faecalibacterium.S.prausnitzii*	1.52	0.028	4447072	*G.Bacteroides.S.uniformis*	-1.29	0.020
2676430	*G.Veillonella.S.dispar*	1.49	0.034	588216	*F.Enterobacteriaceae*	-1.78	0.020
15431	*G.Streptococcus*	1.53	0.038	696563	*G.Blautia.S.producta*	-1.57	0.021
285497	*F.Caulobacteraceae*	0.99	0.043	197072	*G.Bacteroides.S.uniformis*	-1.40	0.023
3506872	*G.Veillonella.S.dispar*	1.23	0.043	NROTU7	*G.Coprococcus*	-1.06	0.025
1063759	*G.Corynebacterium*	1.27	0.044	320395	*G.Bacteroides.S.uniformis*	-1.38	0.028
917641	*G.Staphylococcus*	1.41	0.050	231787	*F.Enterobacteriaceae*	-1.74	0.028
996487	*G.Staphylococcus*	1.46	0.052	NCROTU4975	*G.Bacteroides.S.uniformis*	-1.46	0.031
861807	*G.Corynebacterium*	1.35	0.067	362997	*G.Bacteroides*	-1.23	0.034
585419	*G.Veillonella.S.dispar*	1.43	0.075	3531225	*F.Enterobacteriaceae*	-1.64	0.036
1082539	*G.Streptococcus*	0.66	0.076	577710	*G.Blautia.S.producta*	-1.03	0.037
4321400	*G.Streptococcus*	1.04	0.077	589071	*G.Bacteroides.S.uniformis*	-1.56	0.039
888300	*G.Streptococcus*	1.05	0.079	299267	*F.Enterobacteriaceae*	-1.51	0.042
903426	*G.Rothia.S.mucilaginosa*	1.32	0.111	562376	*G.Dorea*	-1.33	0.043
1108960	*G.Sphingomonas*	0.76	0.118	1109247	*F.Enterobacteriaceae*	-1.58	0.045
537894	*G.Streptococcus*	0.98	0.122	1142029	*G.Bifidobacterium*	-1.64	0.047
1110317	*G.Lactobacillus*	1.19	0.123	2283111	*G.Bacteroides.S.uniformis*	-1.20	0.047
737912	*F.Enterobacteriaceae*	0.88	0.124	348027	*G.Bacteroides.S.uniformis*	-1.11	0.050
4316391	*G.Veillonella.S.dispar*	1.00	0.125	3483793	*F.Enterobacteriaceae*	-1.49	0.059
1101669	*F.Gemellaceae*	1.17	0.127	289709	*G.Escherichia.S.coli*	-1.47	0.060
539107	*F.Enterobacteriaceae*	0.63	0.134	211191	*F.Ruminococcaceae*	-0.93	0.063
757622	*G.Veillonella.S.dispar*	1.12	0.134	332588	*G.Bacteroides.S.uniformis*	-1.00	0.065
4388775	*G.Veillonella.S.dispar*	0.78	0.135	344154	*G.Bacteroides.S.uniformis*	-1.11	0.066
780650	*F.Clostridiaceae*	1.15	0.136	646549	*G.Pseudomonas*	-0.93	0.066
4473975	*G.Enterococcus*	0.77	0.138	554338	*G.Blautia*	-1.34	0.066
925707	*G.Streptococcus*	0.86	0.142	345362	*F.Enterobacteriaceae*	-1.37	0.069
414943	*G.Haemophilus*	0.89	0.149	4371046	*G.Bacteroides.S.uniformis*	-1.21	0.070
				548587	*G.[Eubacterium].S.dolichum*	-1.39	0.071
				336012	*G.Bacteroides.S.uniformis*	-0.91	0.078
				523589	*G.Clostridium.S.neonatale*	-1.43	0.080
				4420408	*G.Bacteroides*	-1.16	0.080
				364179	*G.Bacteroides.S.caccae*	-1.16	0.086
				328617	*G.Bacteroides.S.uniformis*	-0.87	0.091
				531722	*G.Bacteroides.S.ovatus*	-0.85	0.096
				2689396	*F.Enterobacteriaceae*	-0.86	0.098
				304641	*G.Escherichia.S.coli*	-1.11	0.098
				1111294	*G.Escherichia.S.coli*	-1.34	0.100
				NROTU35	*G.Blautia.S.producta*	-0.90	0.107
				4454531	*F.Enterobacteriaceae*	-1.02	0.112
				141145	*F.Enterobacteriaceae*	-1.25	0.114
				782953	*F.Enterobacteriaceae*	-1.25	0.117
				364029	*G.Bacteroides*	-0.82	0.119
				436032	*G.Blautia*	-0.76	0.119
				587530	*G.[Eubacterium].S.dolichum*	-0.70	0.122
				4294457	*G.Rothia.S.mucilaginosa*	-1.09	0.128
				4278525	*G.Bacteroides*	-0.92	0.130
				370183	*G.Blautia*	-0.97	0.133
				1107335	*G.Acinetobacter.S.rhizosphaerae*	-0.79	0.133
				189384	*G.Bacteroides*	-0.87	0.136
				332732	*G.Bacteroides*	-0.79	0.139
				173654	*F.Enterobacteriaceae*	-1.05	0.142
				326662	*G.Bacteroides.S.uniformis*	-0.88	0.145
				4334711	*G.Bacteroides*	-0.86	0.147

*NROTU = New.ReferenceOTU

**NCROTU = New.CleanUp.ReferenceOTU

***O., *F.*, *G.*, and *S.* in taxonomy labels indicate that the level of taxonomy is order, family, genus, or species.

Table S2l. Relation of maternal whole grain intake with infant stool microbial OTUs, in infants delivered vaginally (n = 97)

Positive Associations				Negative Associations			
**OTU**	**Taxonomy*** **	**Coefficient**	**p-value**	**OTU**	**Taxonomy*** **	**Coefficient**	**p-value**
579608	*G.Streptococcus*	3.07	0.024	4385577	*F.Lachnospiraceae*	-3.64	0.015
1649772	*G.Escherichia.S.coli*	3.14	0.024	1839271	*G.[Ruminococcus].S.gnavus*	-3.46	0.015
835880	*F.Enterobacteriaceae*	2.30	0.031	4426874	*G.[Ruminococcus].S.gnavus*	-2.88	0.022
1029949	*G.Lachnospira*	2.42	0.041	184729	*F.Lachnospiraceae*	-2.84	0.022
15431	*G.Streptococcus*	3.16	0.057	1551841	*G.[Ruminococcus].S.gnavus*	-2.93	0.030
4472685	*G.Streptococcus*	2.83	0.071	327851	*G.Streptococcus*	-1.82	0.034
2656868	*G.Bacteroides*	2.18	0.074	176704	*G.[Ruminococcus].S.gnavus*	-2.57	0.048
NROTU23	*F.Lachnospiraceae*	3.15	0.074	182517	*G.[Ruminococcus].S.gnavus*	-2.86	0.053
4440670	*G.Veillonella*	2.28	0.075	3376513	*G.[Ruminococcus].S.gnavus*	-2.51	0.053
342427	*G.Veillonella.S.dispar*	2.18	0.092	331575	*G.[Ruminococcus].S.gnavus*	-2.49	0.054
3583645	*G.Bacteroides*	2.48	0.102	342380	*G.Blautia*	-1.95	0.062
305946	*G.Bacteroides*	2.36	0.109	703741	*G.Lactobacillus*	-2.08	0.070
963344	*G.Enhydrobacter*	2.09	0.109	1027587	*G.Streptococcus*	-2.16	0.073
17309	*G.Lactobacillus*	2.02	0.118	1059729	*G.Granulicatella*	-2.81	0.074
4454531	*F.Enterobacteriaceae*	2.26	0.120	328617	*G.Bacteroides.S.uniformis*	-2.04	0.077
350832	*F.Clostridiaceae*	1.52	0.137	183651	*G.Blautia*	-1.76	0.079
134265	*G.Prevotella*	1.76	0.138	380567	*G.Corynebacterium*	-2.31	0.079
516814	*G.Streptococcus*	1.54	0.139	302880	*G.Streptococcus*	-1.46	0.082
1108960	*G.Sphingomonas*	1.60	0.147	2683271	*G.[Ruminococcus].S.gnavus*	-2.39	0.095
4303016	*G.Streptococcus*	2.48	0.150	1654474	*G.[Ruminococcus].S.gnavus*	-2.46	0.095
				191999	*F.Lachnospiraceae*	-1.99	0.096
				369429	*G.[Ruminococcus]*	-2.51	0.098
				NCROTU4270	*G.Clostridium.S.neonatale*	-1.65	0.099
				2575651	*G.[Ruminococcus].S.gnavus*	-2.23	0.101
				NCROTU1450	*F.Clostridiaceae*	-1.34	0.105
				4376828	*G.Bifidobacterium*	-2.19	0.108
				217734	*G.Streptococcus.S.anginosus*	-2.36	0.119
				189403	*G.[Ruminococcus].S.gnavus*	-1.80	0.121
				1097359	*G.Acinetobacter*	-1.57	0.123
				298427	*G.Enterococcus*	-1.55	0.126
				365181	*G.Collinsella.S.aerofaciens*	-2.20	0.136
				4310208	*G.Veillonella*	-1.76	0.146
				875735	*G.Actinomyces*	-1.87	0.149
				4476604	*O.Clostridiales*	-1.67	0.149

*NROTU = New.ReferenceOTU

**NCROTU = New.CleanUp.ReferenceOTU

***O., *F*., *G*., and *S*. in taxonomy labels indicate that the level of taxonomy is order, family, genus, or species.

Table S3. Relation of microbial community composition in six week old infants delivered by Cesarean section with maternal diet (n = 48)

**Dietary Factor**	***p*-value**[1,2]
aMED Score	0.67
Dairy	0.034
Fruit	0.76
Vegetables	0.94
Whole Grains	0.88
Fish and Seafood	0.75
Nuts, Legumes, and Soy	0.39
Red and Processed Meat	0.91
Polyunsaturated Fat	0.27
EPA	0.66
DHA	0.71
MUFA:SFA Ratio	0.50

[1]All *p*-values are determined by PERMANOVA

[2]*p*-values are adjusted for infant feeding method, maternal BMI, parity, and batch

Table S4. Infant gut microbiome cluster is influenced by maternal diet.

Dietary Factor	OR (95% Confidence Interval)[2] Cluster 2	Cluster 3
aMED score	0.98 (0.54,1.77)	1.03 (0.63,1.68)
Dairy	2.36 (1.05,5.30)[3]	1.87 (0.96,3.62)[3]
Fruit	0.55 (0.20,1.55)	0.69 (0.33,1.46)
Vegetables	0.72 (0.32,1.62)	0.93 (0.59,1.47)
Whole Grains	0.29 (0.05,1.90)	1.83 (0.52,6.45)
Fish and Seafood	25.15 (0.10,6379.60)	0.03 (0.00,11.03)
Nuts, Legumes, and Soy	0.99 (0.38,2.58)	0.86 (0.33,2.22)
Red and Processed Meat	1.19 (0.15,9.58)	0.88 (0.16,4.81)
Polyunsaturated fat	0.91 (0.68,1.23)	1.14 (0.91,1.42)
EPA	1.49 (0.00,3050.80)	0.01 (0.00,27.06)
DHA	3.90 (0.00,3996.77)	0.19 (0.00,119.54)
MUFA:SFA ratio	1.01 (0.06,18.34)	0.93 (0.07,11.79)

[1]Models include infants delivered by Cesarean section (n = 48)
[2]Cluster 1 is the reference group
[3]Corresponds to *Figure 2b*

Table S5a. Relation of maternal aMED score with infant stool microbial OTUs, in infants delivered by cesarean (n = 48)

Positive Associations				Negative Associations			
**OTU**	**Taxonomy*** **	**Coefficient**	**p-value**	**OTU**	**Taxonomy*** **	**Coefficient**	**p-value**
1696853	*G.Enterococcus*	2.55	1.6E-04	369027	*F.Lachnospiraceae*	-2.56	7.2E-04
949863	*G.Lactobacillus.S.zeae*	3.47	2.0E-03	289709	*G.Escherichia.S.coli*	-4.25	8.9E-04
336632	*G.Akkermansia.S.muciniphila*	2.04	3.2E-03	141145	*F.Enterobacteriaceae*	-4.15	1.3E-03
NROTU14	*F.Lachnospiraceae*	2.03	0.011	4308688	*G.Bifidobacterium*	-1.35	4.6E-03
NCROTU4061	*G.Bacteroides*	1.24	0.012	4472685	*G.Streptococcus*	-3.19	4.9E-03
362767	*F.Lachnospiraceae*	2.95	0.015	114510	*F.Enterobacteriaceae*	-3.64	7.2E-03
NROTU25	*F.Lachnospiraceae*	1.84	0.024	588216	*F.Enterobacteriaceae*	-3.35	9.3E-03
377546	*F.Caulobacteraceae*	1.36	0.026	4111715	*F.Enterobacteriaceae*	-2.74	0.016
270094	*G.Bacteroides*	1.74	0.029	1108656	*F.Enterobacteriaceae*	-2.89	0.017
10085	*F.Enterobacteriaceae*	1.97	0.044	383714	*G.Anaerococcus*	-1.63	0.017
861807	*G.Corynebacterium*	2.37	0.053	4441855	*G.Streptococcus*	-2.90	0.019
1055212	*G.Enterococcus*	1.76	0.055	941096	*G.Streptococcus*	-2.53	0.021
12574	*G.Actinomyces*	2.46	0.061	538000	*F.Enterobacteriaceae*	-2.88	0.022
4433947	*G.Bacteroides*	0.86	0.072	1109247	*F.Enterobacteriaceae*	-3.24	0.023
4345397	*F.Enterobacteriaceae*	1.11	0.072	513500	*G.Streptococcus*	-2.60	0.026
4349891	*G.Lactobacillus*	1.58	0.074	4333897	*F.Enterobacteriaceae*	-2.59	0.032
331575	*G.[Ruminococcus].S.gnavus*	1.15	0.075	604966	*G.Lactobacillus*	-1.86	0.033
835771	*F.Enterobacteriaceae*	1.28	0.082	231787	*F.Enterobacteriaceae*	-2.80	0.035
NROTU15	*G.Streptococcus*	1.50	0.083	782953	*F.Enterobacteriaceae*	-2.70	0.040
949789	*G.Enterococcus*	1.40	0.085	1085410	*G.Streptococcus*	-1.65	0.040
4333020	*F.Enterobacteriaceae*	1.91	0.091	183651	*G.Blautia*	-1.73	0.045
1111582	*G.Enterococcus*	0.72	0.094	797229	*F.Enterobacteriaceae*	-1.26	0.049
				356760	*F.Erysipelotrichaceae*	-2.38	0.056
				302683	*G.Blautia*	-1.41	0.064
				193466	*G.Blautia*	-1.71	0.067
				4303016	*G.Streptococcus*	-2.06	0.078
				3483793	*F.Enterobacteriaceae*	-2.23	0.078
				972033	*G.Streptococcus*	-2.06	0.079
				299267	*F.Enterobacteriaceae*	-2.26	0.080
				1078587	*G.Blautia*	-1.72	0.080
				1111294	*G.Escherichia.S.coli*	-2.38	0.083
				92535	*G.Streptococcus*	-2.09	0.084
				563654	*G.Lactobacillus*	-1.58	0.085
				916151	*G.Veillonella*	-1.39	0.086
				780650	*F.Clostridiaceae*	-2.33	0.095

*NROTU = New.ReferenceOTU

**NCROTU = New.CleanUp.ReferenceOTU

***O., *F*., *G*., and *S*. in taxonomy labels indicate that the level of taxonomy is order, family, genus, or species.

Table S5b. Relation of maternal dairy intake with infant stool microbial OTUs, in infants delivered by cesarean (n = 48)

Positive Associations				Negative Associations			
**OTU**	**Taxonomy*** **	**Coefficient**	**p-value**	**OTU**	**Taxonomy*** **	**Coefficient**	**p-value**
1107335	*G.Acinetobacter.S.rhizosphaerae*	2.01	4.0E-03	861807	*G.Corynebacterium*	-3.45	4.4E-03
359175	*F.Ruminococcaceae*	1.99	6.1E-03	1017249	*G.Bifidobacterium*	-3.20	8.6E-03
4333897	*F.Enterobacteriaceae*	3.26	6.8E-03	769643	*G.Pseudomonas*	-2.16	0.011
173654	*F.Enterobacteriaceae*	2.42	0.017	646549	*G.Pseudomonas*	-1.52	0.019
511795	*G.Streptococcus.S.anginosus*	2.71	0.018	NROTU27	*F.Enterobacteriaceae*	-1.59	0.035
538000	*F.Enterobacteriaceae*	3.02	0.019	1055212	*G.Enterococcus*	-1.95	0.037
1791578	*F.Enterobacteriaceae*	1.67	0.020	NROTU25	*F.Lachnospiraceae*	-1.74	0.038
304641	*G.Escherichia.S.coli*	1.74	0.022	903426	*G.Rothia.S.mucilaginosa*	-2.42	0.040
588216	*F.Enterobacteriaceae*	2.97	0.025	4433947	*G.Bacteroides*	-0.98	0.041
1028632	*G.Escherichia.S.coli*	2.66	0.028	484304	*G.Bifidobacterium*	-2.39	0.054
4454531	*F.Enterobacteriaceae*	1.93	0.030	1075821	*G.Alloiococcus*	-2.43	0.067
295053	*F.Enterobacteriaceae*	2.01	0.039	817734	*G.Pseudomonas*	-1.48	0.088
4457268	*F.Enterobacteriaceae*	1.78	0.040	577710	*G.Blautia.S.producta*	-1.37	0.089
3531225	*F.Enterobacteriaceae*	2.76	0.041	270094	*G.Bacteroides*	-1.39	0.090
NROTU11	*F.Enterobacteriaceae*	1.36	0.048	949863	*G.Lactobacillus.S.zeae*	-2.01	0.092
529979	*F.Erysipelotrichaceae*	1.78	0.050	292521	*G.Bifidobacterium*	-1.10	0.101
465079	*G.Staphylococcus.S.aureus*	1.51	0.052	544493	*F.Oxalobacteraceae*	-1.00	0.104
782953	*F.Enterobacteriaceae*	2.55	0.057	234488	*G.Bacteroides*	-0.57	0.107
541328	*G.Clostridium.S.neonatale*	1.91	0.059	183651	*G.Blautia*	-1.43	0.108
2676432	*F.Clostridiaceae*	2.05	0.060	359538	*G.Bacteroides.S.caccae*	-1.58	0.109
1059655	*G.Streptococcus*	1.44	0.061	4334711	*G.Bacteroides*	-0.54	0.110
442743	*F.Enterobacteriaceae*	1.58	0.061	3745352	*G.Bacteroides*	-0.52	0.113
975306	*G.Roseburia.S.faecis*	2.06	0.066	997439	*G.Bifidobacterium*	-2.11	0.115
289709	*G.Escherichia.S.coli*	2.46	0.073	197273	*G.Streptococcus*	-1.90	0.118
1083508	*F.Xanthomonadaceae*	1.55	0.077	2656868	*G.Bacteroides*	-0.87	0.119
1084865	*G.Staphylococcus*	2.01	0.079	339013	*G.Bacteroides.S.ovatus*	-1.93	0.121
NCROTU2292	*F.Clostridiaceae*	2.27	0.079	130468	*G.Lactobacillus*	-1.31	0.122
114510	*F.Enterobacteriaceae*	2.45	0.084	4316391	*G.Veillonella.S.dispar*	-1.79	0.125
350832	*F.Clostridiaceae*	1.58	0.087	182517	*G.[Ruminococcus].S.gnavus*	-1.21	0.128
852030	*G.Staphylococcus*	1.64	0.089	876714	*G.Pseudomonas*	-1.66	0.129
835880	*F.Enterobacteriaceae*	1.63	0.096	189971	*G.Blautia*	-1.26	0.139
241415	*F.Enterobacteriaceae*	1.70	0.098	NROTU20	*F.Lachnospiraceae*	-1.31	0.144
260410	*G.Bacteroides.S.ovatus*	1.19	0.103	573270	*O.Burkholderiales*	-0.85	0.147
801438	*F.Enterobacteriaceae*	1.86	0.105	NROTU29	*G.Bacteroides.S.caccae*	-1.60	0.148
141145	*F.Enterobacteriaceae*	2.18	0.115				
NCROTU1492	*F.Enterobacteriaceae*	1.30	0.118				
327851	*G.Streptococcus*	1.46	0.124				
178478	*F.Rikenellaceae*	0.73	0.129				
4388775	*G.Veillonella.S.dispar*	1.34	0.139				
1040220	*O.Bacillales*	1.36	0.140				
356760	*F.Erysipelotrichaceae*	1.89	0.140				
984924	*G.Staphylococcus*	1.46	0.146				

*NROTU = New.ReferenceOTU

**NCROTU = New.CleanUp.ReferenceOTU

***O., *F*., *G*., and *S*. in taxonomy labels indicate that the level of taxonomy is order, family, genus, or species.

Table S5c. Relation of maternal fish and seafood intake with infant stool microbial OTUs, in infants delivered by cesarean (n = 48)

Positive Associations				Negative Associations			
**OTU**	**Taxonomy*** **	**Coefficient**	**p-value**	**OTU**	**Taxonomy*** **	**Coefficient**	**p-value**
302880	*G.Streptococcus*	30.82	1.7E-04	958584	*G.Clostridium.S.neonatale*	-28.45	0.011
1076969	*G.Streptococcus*	28.18	3.9E-03	4111715	*F.Enterobacteriaceae*	-25.27	0.026
NROTU15	*G.Streptococcus*	23.72	3.9E-03	894969	*G.Streptococcus*	-15.35	0.031
567972	*G.Streptococcus.S.agalactiae*	31.65	4.5E-03	NCROTU4270	*G.Clostridium.S.neonatale*	-14.65	0.031
743120	*F.Enterobacteriaceae*	16.31	6.2E-03	541328	*G.Clostridium.S.neonatale*	-20.61	0.035
327851	*G.Streptococcus*	23.87	7.2E-03	369027	*F.Lachnospiraceae*	-16.00	0.040
332718	*G.Streptococcus*	25.36	9.4E-03	1073276	*G.Streptococcus*	-17.96	0.041
173744	*G.Megasphaera*	15.90	0.011	NCROTU3657	*G.Clostridium.S.butyricum*	-14.90	0.045
328617	*G.Bacteroides.S.uniformis*	7.45	0.011	1078207	*G.Streptococcus*	-15.84	0.047
2876801	*G.Bacteroides.S.uniformis*	7.45	0.011	4303016	*G.Streptococcus*	-22.12	0.054
312140	*G.Bacteroides*	7.45	0.011	523140	*G.Ruminococcus*	-18.86	0.057
336012	*G.Bacteroides.S.uniformis*	7.45	0.011	878104	*G.Veillonella.S.dispar*	-17.88	0.067
194909	*G.Bacteroides*	7.45	0.011	889025	*G.Acinetobacter*	-16.91	0.068
2137001	*G.Bacteroides*	7.62	0.011	299267	*F.Enterobacteriaceae*	-21.44	0.093
3940440	*G.Bacteroides*	7.62	0.011	291090	*G.Parabacteroides.S.distasonis*	-16.63	0.095
184753	*G.Bacteroides*	7.62	0.011				
161423	*G.Bacteroides*	7.72	0.012				
NCROTU3323	*G.Bacteroides*	7.72	0.012				
177150	*G.Bacteroides*	7.72	0.012				
1566189	*G.Bacteroides*	7.72	0.012				
3272632	*G.Bacteroides*	7.72	0.012				
190638	*G.Bacteroides*	7.72	0.012				
4312969	*G.Staphylococcus*	19.32	0.012				
199716	*G.Bacteroides*	7.79	0.012				
844375	*G.Bacteroides*	7.79	0.012				
560336	*G.Bacteroides*	8.23	0.013				
4060124	*G.Bacteroides*	8.33	0.013				
513445	*G.Bacteroides*	14.11	0.016				
271214	*G.Bacteroides*	16.13	0.019				
349024	*G.Streptococcus*	28.18	0.022				
197052	*G.Bacteroides*	10.29	0.027				
365181	*G.Collinsella.S.aerofaciens*	14.45	0.044				
841907	*G.Bilophila*	7.63	0.049				
1995363	*G.Staphylococcus.S.aureus*	18.51	0.054				
NCROTU835	*F.Enterobacteriaceae*	15.87	0.054				
4305815	*G.Streptococcus*	9.66	0.056				
1090059	*G.Enterococcus*	15.35	0.061				
1055824	*G.Staphylococcus*	16.12	0.065				
4316391	*G.Veillonella.S.dispar*	20.66	0.066				
4440670	*G.Veillonella*	16.20	0.068				
953855	*F.Rikenellaceae*	14.52	0.071				
1058950	*G.Staphylococcus*	13.95	0.072				
362767	*F.Lachnospiraceae*	21.67	0.075				
548587	*G.[Eubacterium].S.dolichum*	20.01	0.075				
NCROTU4061	*G.Bacteroides*	8.69	0.080				
176775	*G.Phascolarctobacterium*	13.55	0.081				
554338	*G.Blautia*	20.41	0.088				
2283111	*G.Bacteroides.S.uniformis*	6.15	0.089				
326662	*G.Bacteroides.S.uniformis*	9.59	0.096				
1075821	*G.Alloiococcus*	21.39	0.097				

*NROTU = New.ReferenceOTU

**NCROTU = New.CleanUp.ReferenceOTU

***O., *F.*, *G.*, and *S.* in taxonomy labels indicate that the level of taxonomy is order, family, genus, or species.

Table S5d. Relation of maternal fruit intake with infant stool microbial OTUs, in infants delivered by cesarean (n = 48)

Positive Associations				Negative Associations			
**OTU**	**Taxonomy*** **	**Coefficient**	**p-value**	**OTU**	**Taxonomy*** **	**Coefficient**	**p-value**
554338	*G.Blautia*	5.47	1.6E-03	513500	*G.Streptococcus*	-5.64	7.7E-04
NROTU25	*F.Lachnospiraceae*	3.07	0.012	289709	*G.Escherichia.S.coli*	-5.82	2.7E-03
102049	*G.Bifidobacterium*	1.70	0.015	538000	*F.Enterobacteriaceae*	-5.09	6.3E-03
949863	*G.Lactobacillus.S.zeae*	4.16	0.016	588216	*F.Enterobacteriaceae*	-4.68	0.016
NROTU14	*F.Lachnospiraceae*	2.82	0.019	941096	*G.Streptococcus*	-3.84	0.019
1055212	*G.Enterococcus*	3.07	0.024	231787	*F.Enterobacteriaceae*	-4.48	0.023
526583	*F.Clostridiaceae*	2.95	0.027	3483793	*F.Enterobacteriaceae*	-4.22	0.024
1027587	*G.Streptococcus*	1.84	0.031	141145	*F.Enterobacteriaceae*	-4.44	0.027
342397	*G.[Ruminococcus].S.gnavus*	3.44	0.033	1108656	*F.Enterobacteriaceae*	-3.97	0.029
364926	*G.Bacteroides*	3.92	0.037	1147925	*F.Clostridiaceae*	-3.24	0.029
316675	*F.Peptostreptococcaceae*	3.23	0.043	187035	*G.Blautia*	-2.60	0.036
531722	*G.Bacteroides.S.ovatus*	1.56	0.050	1064036	*G.Peptoniphilus*	-3.18	0.037
577170	*G.Bacteroides*	1.34	0.054	3171486	*F.Enterobacteriaceae*	-3.13	0.039
360015	*G.[Ruminococcus].S.gnavus*	2.99	0.066	302683	*G.Blautia*	-2.28	0.044
232696	*F.Enterobacteriaceae*	2.95	0.066	1047077	*G.Actinomyces*	-3.26	0.046
226338	*G.Enterococcus*	2.85	0.067	2689396	*F.Enterobacteriaceae*	-2.29	0.047
1696853	*G.Enterococcus*	1.93	0.076	NCROTU1008	*G.Blautia*	-2.44	0.048
309720	*F.Ruminococcaceae*	1.96	0.084	4457268	*F.Enterobacteriaceae*	-2.52	0.049
746679	*F.Enterobacteriaceae*	3.10	0.094	383714	*G.Anaerococcus*	-2.01	0.052
270094	*G.Bacteroides*	1.99	0.099	4454531	*F.Enterobacteriaceae*	-2.54	0.053
				183651	*G.Blautia*	-2.49	0.055
				1109247	*F.Enterobacteriaceae*	-4.12	0.055
				114510	*F.Enterobacteriaceae*	-3.99	0.055
				345362	*F.Enterobacteriaceae*	-3.04	0.065
				359175	*F.Ruminococcaceae*	-2.01	0.068
				4472685	*G.Streptococcus*	-3.19	0.068
				782953	*F.Enterobacteriaceae*	-3.60	0.068
				3531225	*F.Enterobacteriaceae*	-3.60	0.071
				925707	*G.Streptococcus*	-2.25	0.080
				4326406	*G.Streptococcus*	-2.21	0.090
				1625448	*F.Clostridiaceae*	-2.77	0.097

*NROTU = New.ReferenceOTU

**NCROTU = New.CleanUp.ReferenceOTU

***O., *F.*, *G.*, and *S.* in taxonomy labels indicate that the level of taxonomy is order, family, genus, or species.

Table S5e. Relation of maternal red and processed meat intake with infant stool microbial OTUs, in infants delivered by cesarean (n = 48)

Positive Associations				Negative Associations			
**OTU**	**Taxonomy*** **	**Coefficient**	**p-value**	**OTU**	**Taxonomy*** **	**Coefficient**	**p-value**
1028632	*G.Escherichia.S.coli*	13.81	4.1E-04	703635	*F.Enterobacteriaceae*	-7.59	3.1E-03
231787	*F.Enterobacteriaceae*	14.63	8.6E-04	835771	*F.Enterobacteriaceae*	-6.64	7.2E-03
1108656	*F.Enterobacteriaceae*	12.23	2.8E-03	526682	*G.Actinomyces*	-7.37	8.3E-03
701221	*G.Roseburia*	10.49	3.5E-03	192342	*F.Enterobacteriaceae*	-8.29	0.012
442743	*F.Enterobacteriaceae*	8.09	3.6E-03	656517	*F.Enterobacteriaceae*	-7.78	0.016
4111715	*F.Enterobacteriaceae*	11.21	3.9E-03	889025	*G.Acinetobacter*	-7.57	0.018
334656	*G.Enterococcus*	7.53	5.0E-03	4328189	*F.Enterobacteriaceae*	-5.97	0.019
15257	*G.Enterococcus*	11.38	5.4E-03	152859	*F.Enterobacteriaceae*	-6.54	0.027
345362	*F.Enterobacteriaceae*	10.12	6.1E-03	967427	*G.Streptococcus*	-5.23	0.029
1109247	*F.Enterobacteriaceae*	13.19	6.4E-03	1064036	*G.Peptoniphilus*	-7.52	0.033
369429	*G.[Ruminococcus]*	11.15	7.3E-03	103166	*F.Enterobacteriaceae*	-4.77	0.035
289709	*G.Escherichia.S.coli*	12.06	7.9E-03	274754	*F.Enterobacteriaceae*	-8.37	0.039
851865	*G.Faecalibacterium.S.prausnitzii*	10.10	0.010	164789	*F.Enterobacteriaceae*	-6.19	0.043
141145	*F.Enterobacteriaceae*	11.67	0.011	878104	*G.Veillonella.S.dispar*	-6.86	0.044
606927	*F.Peptostreptococcaceae*	11.48	0.013	315429	*G.Bacteroides*	-5.00	0.054
538000	*F.Enterobacteriaceae*	10.63	0.014	10085	*F.Enterobacteriaceae*	-6.51	0.055
3483793	*F.Enterobacteriaceae*	10.50	0.014	969149	*F.Enterobacteriaceae*	-6.02	0.056
782953	*F.Enterobacteriaceae*	10.91	0.015	813457	*F.Enterobacteriaceae*	-5.64	0.057
114510	*F.Enterobacteriaceae*	11.43	0.016	331697	*F.Enterobacteriaceae*	-7.94	0.062
4308688	*G.Bifidobacterium*	4.01	0.016	86428	*G.Veillonella.S.dispar*	-7.61	0.062
196176	*G.Dorea*	10.94	0.017	1047077	*G.Actinomyces*	-7.03	0.063
299267	*F.Enterobacteriaceae*	10.44	0.017	4357712	*G.Bacteroides*	-2.68	0.065
1111294	*G.Escherichia.S.coli*	10.83	0.020	4473176	*F.Enterobacteriaceae*	-5.54	0.066
228894	*G.Enterococcus*	7.88	0.022	4359220	*G.Veillonella.S.dispar*	-5.68	0.069
797229	*F.Enterobacteriaceae*	5.05	0.022	258785	*F.Enterobacteriaceae*	-5.77	0.071
641490	*G.Enterococcus*	6.31	0.023	759061	*F.Enterobacteriaceae*	-7.71	0.072
4303016	*G.Streptococcus*	9.01	0.024	NROTU20	*F.Lachnospiraceae*	-5.43	0.073
304641	*G.Escherichia.S.coli*	5.82	0.025	320395	*G.Bacteroides.S.uniformis*	-3.59	0.074
369027	*F.Lachnospiraceae*	5.94	0.029	988542	*G.Haemophilus.S.parainfluenzae*	-6.82	0.075
766768	*G.Enterococcus*	8.90	0.031	2875735	*G.Bacteroides*	-3.69	0.077
NROTU24	*G.Enterococcus*	7.42	0.036	2624257	*G.Bacteroides*	-3.66	0.077
254662	*F.Enterobacteriaceae*	8.87	0.037	544493	*F.Oxalobacteraceae*	3.67	0.079
573270	*O.Burkholderiales*	4.06	0.039	4294457	*G.Rothia.S.mucilaginosa*	-7.44	0.082
1067519	*G.Staphylococcus*	7.03	0.040	176704	*G.[Ruminococcus].S.gnavus*	-3.37	0.082
996487	*G.Staphylococcus*	7.73	0.044	4476604	*O.Clostridiales*	-3.23	0.084
696563	*G.Blautia.S.producta*	7.65	0.044	12574	*G.Actinomyces*	-7.83	0.086
4413347	*G.Bifidobacterium*	5.70	0.045	876714	*G.Pseudomonas*	-6.27	0.089
588216	*F.Enterobacteriaceae*	9.08	0.045	1625448	*F.Clostridiaceae*	-6.53	0.090
958584	*G.Clostridium.S.neonatale*	7.99	0.045	3887769	*G.Bacteroides*	-3.36	0.091
628226	*F.Peptostreptococcaceae*	7.63	0.045	211706	*G.Bacteroides*	-3.41	0.091
NROTU36	*F.Lachnospiraceae*	5.62	0.053	518002	*F.Enterobacteriaceae*	-6.46	0.094
NCReOTU1492	*F.Enterobacteriaceae*	5.38	0.055	NROTU15	*G.Streptococcus*	-5.00	0.095
4433947	*G.Bacteroides*	3.10	0.059	425721	*F.Enterobacteriaceae*	-6.06	0.096
3531225	*F.Enterobacteriaceae*	8.46	0.066	224670	*F.Enterobacteriaceae*	-5.41	0.096
183651	*G.Blautia*	5.43	0.070	303379	*F.Clostridiaceae*	-6.42	0.098
4376828	*G.Bifidobacterium*	4.73	0.086	814442	*F.Enterobacteriaceae*	-3.59	0.100
339532	*G.Bifidobacterium*	5.86	0.086				
1649772	*G.Escherichia.S.coli*	4.99	0.088				
548587	*G.[Eubacterium].S.dolichum*	6.59	0.096				
589277	*G.Bacteroides*	4.33	0.097				

*NROTU = New.ReferenceOTU

**NCROTU = New.CleanUp.ReferenceOTU

***O., *F.*, *G.*, and *S.* in taxonomy labels indicate that the level of taxonomy is order, family, genus, or species.

Table S5f. Relation of maternal MUFA:SFA ratio with infant stool microbial OTUs, in infants delivered by cesarean (n = 48)

Positive Associations				Negative Associations			
OTU	Taxonomy***	Coefficient	p-value	OTU	Taxonomy***	Coefficient	p-value
582691	*F.Clostridiaceae*	17.87	1.0E-03	132041	*G.Bifidobacterium*	-10.76	8.1E-03
148620	*F.Enterobacteriaceae*	8.98	1.1E-03	356760	*F.Erysipelotrichaceae*	-15.31	0.012
187623	*G.Bacteroides*	12.52	1.3E-03	958584	*G.Clostridium.S.neonatale*	-13.79	0.015
322798	*F.Clostridiaceae*	16.86	2.0E-03	NROTU36	*F.Lachnospiraceae*	-10.03	0.015
337909	*G.Clostridium*	15.69	2.8E-03	1649772	*G.Escherichia.S.coli*	-9.57	0.021
712047	*F.Clostridiaceae*	15.59	3.2E-03	588216	*F.Enterobacteriaceae*	-13.92	0.033
315982	*F.Clostridiaceae*	15.42	3.6E-03	917641	*G.Staphylococcus*	-10.87	0.034
345448	*F.Clostridiaceae*	15.10	5.2E-03	114510	*F.Enterobacteriaceae*	-14.50	0.036
355471	*F.Clostridiaceae*	15.02	5.8E-03	NROTU2	*F.Erysipelotrichaceae*	-12.75	0.036
532521	*G.Peptostreptococcus.S.anaerobius*	6.74	0.010	NCROTU4270	*G.Clostridium.S.neonatale*	-7.19	0.037
4328189	*F.Enterobacteriaceae*	9.15	0.013	604966	*G.Lactobacillus*	-9.07	0.037
1095073	*G.Propionibacterium.S.acnes*	10.00	0.013	716006	*G.Lactococcus*	-9.89	0.048
304779	*F.Clostridiaceae*	12.58	0.014	3908638	*F.Enterobacteriaceae*	-9.50	0.048
828483	*O.Clostridiales*	14.90	0.019	173654	*F.Enterobacteriaceae*	-9.88	0.050
810399	*G.Enterococcus*	11.45	0.023	975306	*G.Roseburia.S.faecis*	-10.71	0.050
183480	*G.Bacteroides*	7.55	0.026	295053	*F.Enterobacteriaceae*	-9.16	0.055
1147925	*F.Clostridiaceae*	10.64	0.032	339532	*G.Bifidobacterium*	-9.18	0.061
524725	*G.Atopobium*	13.01	0.032	4472685	*G.Streptococcus*	-10.85	0.062
NCROTU4696	*G.Bacteroides*	4.95	0.034	851865	*G.Faecalibacterium.S.prausnitzii*	-10.64	0.065
4475758	*G.Veillonella.S.dispar*	11.54	0.036	15431	*G.Streptococcus*	-10.48	0.068
3583645	*G.Bacteroides*	5.00	0.037	1111294	*G.Escherichia.S.coli*	-12.39	0.070
315429	*G.Bacteroides*	7.74	0.038	299267	*F.Enterobacteriaceae*	-11.63	0.071
10085	*F.Enterobacteriaceae*	10.01	0.040	169182	*F.Enterobacteriaceae*	-7.36	0.073
2800178	*G.Bacteroides.S.fragilis*	3.60	0.044	489671	*G.Staphylococcus*	-8.19	0.074
4372578	*G.Bacteroides.S.fragilis*	3.68	0.044	141145	*F.Enterobacteriaceae*	-12.06	0.074
2430693	*G.Bacteroides.S.fragilis*	3.78	0.044	369027	*F.Lachnospiraceae*	-7.09	0.075
183603	*G.Bacteroides.S.fragilis*	3.97	0.045	2250983	*G.Clostridium.S.neonatale*	-8.58	0.076
1105343	*F.Ruminococcaceae*	4.93	0.052	4111715	*F.Enterobacteriaceae*	-10.08	0.083
198423	*G.[Ruminococcus].S.gnavus*	10.69	0.056	1067519	*G.Staphylococcus*	-8.47	0.090
593672	*G.Enterococcus*	8.14	0.059	1078587	*G.Blautia*	-8.31	0.091
292364	*G.Enterococcus*	7.91	0.060	523589	*G.Clostridium.S.neonatale*	-6.57	0.094
511795	*G.Streptococcus.S.anginosus*	10.72	0.061	NCROTU3657	*G.Clostridium.S.butyricum*	-6.34	0.095
835771	*F.Enterobacteriaceae*	6.69	0.068				
369555	*G.Ruminococcus*	5.36	0.075				
1068499	*G.Streptococcus*	10.77	0.076				
365181	*G.Collinsella.S.aerofaciens*	6.28	0.087				
4359220	*G.Veillonella.S.dispar*	7.58	0.094				
285497	*F.Caulobacteraceae*	6.71	0.099				

*NROTU = New.ReferenceOTU

**NCROTU = New.CleanUp.ReferenceOTU

***O., *F.*, *G.*, and *S.* in taxonomy labels indicate that the level of taxonomy is order, family, genus, or species.

Table S5g. Relation of maternal DHA intake with infant stool microbial OTUs, in infants delivered by cesarean (n = 48)

Positive Associations				Negative Associations			
**OTU**	**Taxonomy*** **	**Coefficient**	**p-value**	**OTU**	**Taxonomy*** **	**Coefficient**	**p-value**
NROTU15	*G.Streptococcus*	37.76	2.0E-04	4111715	*F.Enterobacteriaceae*	-35.31	0.014
362767	*F.Lachnospiraceae*	43.58	3.8E-03	369027	*F.Lachnospiraceae*	-24.19	0.014
2582263	*F.Enterobacteriaceae*	25.52	8.1E-03	588216	*F.Enterobacteriaceae*	-39.50	0.015
743120	*F.Enterobacteriaceae*	19.26	0.012	299267	*F.Enterobacteriaceae*	-37.48	0.019
1052663	*G.Staphylococcus*	25.35	0.016	1078587	*G.Blautia*	-28.25	0.021
1995363	*G.Staphylococcus.S.aureus*	28.04	0.021	141145	*F.Enterobacteriaceae*	-37.25	0.027
302880	*G.Streptococcus*	25.25	0.023	1111294	*G.Escherichia.S.coli*	-37.26	0.029
10085	*F.Enterobacteriaceae*	27.51	0.024	231787	*F.Enterobacteriaceae*	-36.18	0.030
320395	*G.Bacteroides.S.uniformis*	15.86	0.029	114510	*F.Enterobacteriaceae*	-37.29	0.032
852030	*G.Staphylococcus*	25.57	0.030	1108656	*F.Enterobacteriaceae*	-32.38	0.035
2875735	*G.Bacteroides*	16.27	0.031	289709	*G.Escherichia.S.coli*	-35.51	0.035
377546	*F.Caulobacteraceae*	16.52	0.032	958584	*G.Clostridium.S.neonatale*	-30.00	0.039
2624257	*G.Bacteroides*	15.97	0.033	523140	*G.Ruminococcus*	-25.95	0.040
861807	*G.Corynebacterium*	32.17	0.036	581079	*G.Oscillospira*	-32.96	0.043
4312969	*G.Staphylococcus*	20.44	0.040	145801	*F.Erysipelotrichaceae*	-19.41	0.060
567972	*G.Streptococcus.S.agalactiae*	29.12	0.047	4303016	*G.Streptococcus*	-27.29	0.063
NROTU20	*F.Lachnospiraceae*	21.79	0.047	NROTU23	*F.Lachnospiraceae*	-30.22	0.066
271214	*G.Bacteroides*	17.69	0.047	1028632	*G.Escherichia.S.coli*	-27.66	0.068
328617	*G.Bacteroides.S.uniformis*	7.55	0.048	NROTU2	*F.Erysipelotrichaceae*	-28.15	0.069
2876801	*G.Bacteroides.S.uniformis*	7.55	0.048	356760	*F.Erysipelotrichaceae*	-28.61	0.069
312140	*G.Bacteroides*	7.55	0.048	345362	*F.Enterobacteriaceae*	-24.38	0.079
336012	*G.Bacteroides.S.uniformis*	7.55	0.048	4308688	*G.Bifidobacterium*	-10.81	0.081
194909	*G.Bacteroides*	7.55	0.048	442743	*F.Enterobacteriaceae*	-18.17	0.083
2137001	*G.Bacteroides*	7.72	0.049	1073276	*G.Streptococcus*	-19.55	0.085
3940440	*G.Bacteroides*	7.72	0.049	4376828	*G.Bifidobacterium*	-17.01	0.090
184753	*G.Bacteroides*	7.72	0.049	1109247	*F.Enterobacteriaceae*	-30.71	0.091
161423	*G.Bacteroides*	7.81	0.050	696563	*G.Blautia.S.producta*	-23.29	0.096
NCROTU3323	*G.Bacteroides*	7.81	0.050				
177150	*G.Bacteroides*	7.81	0.050				
1566189	*G.Bacteroides*	7.81	0.050				
3272632	*G.Bacteroides*	7.81	0.050				
190638	*G.Bacteroides*	7.81	0.050				
1696853	*G.Enterococcus*	17.88	0.050				
199716	*G.Bacteroides*	7.88	0.050				
844375	*G.Bacteroides*	7.88	0.050				
560336	*G.Bacteroides*	8.31	0.053				
4060124	*G.Bacteroides*	8.41	0.054				
1055824	*G.Staphylococcus*	21.28	0.057				
2724175	*G.[Ruminococcus].S.gnavus*	11.09	0.067				
164413	*G.Enterococcus*	20.79	0.068				
4328189	*F.Enterobacteriaceae*	16.94	0.073				
984924	*G.Staphylococcus*	21.09	0.088				
1084906	*G.Staphylococcus*	4.19	0.092				
2283111	*G.Bacteroides.S.uniformis*	7.80	0.093				
NROTU11	*F.Enterobacteriaceae*	14.14	0.099				

*NROTU = New.ReferenceOTU

**NCROTU = New.CleanUp.ReferenceOTU

***O., *F.*, *G.*, and *S.* in taxonomy labels indicate that the level of taxonomy is order, family, genus, or species.

Table S5h. Relation of maternal EPA intake with infant stool microbial OTUs, in infants delivered by cesarean (n = 48)

Positive Associations				Negative Associations			
**OTU**	**Taxonomy*** **	**Coefficient**	**p-value**	**OTU**	**Taxonomy*** **	**Coefficient**	**p-value**
NROTU15	*G.Streptococcus*	52.83	7.2E-07	1078587	*G.Blautia*	-30.78	0.025
377546	*F.Caulobacteraceae*	24.16	4.3E-03	958584	*G.Clostridium.S.neonatale*	-35.53	0.029
320395	*G.Bacteroides.S.uniformis*	22.44	4.9E-03	889025	*G.Acinetobacter*	-27.72	0.036
2875735	*G.Bacteroides*	22.93	5.7E-03	4303016	*G.Streptococcus*	-33.93	0.039
2624257	*G.Bacteroides*	22.55	6.2E-03	145801	*F.Erysipelotrichaceae*	-21.01	0.071
511378	*G.Veillonella*	34.97	8.2E-03	4416562	*F.Enterobacteriaceae*	-28.41	0.078
532521	*G.Peptostreptococcus.S.anaerobius*	19.40	8.6E-03	1111874	*F.Enterobacteriaceae*	-31.69	0.083
1095073	*G.Propionibacterium.S.acnes*	29.90	8.7E-03	4111715	*F.Enterobacteriaceae*	-27.88	0.091
271214	*G.Bacteroides*	25.31	0.010	821080	*F.Enterobacteriaceae*	-27.85	0.093
362767	*F.Lachnospiraceae*	41.66	0.015				
NROTU11	*F.Enterobacteriaceae*	22.87	0.015				
328617	*G.Bacteroides.S.uniformis*	10.23	0.016				
2876801	*G.Bacteroides.S.uniformis*	10.23	0.016				
312140	*G.Bacteroides*	10.23	0.016				
336012	*G.Bacteroides.S.uniformis*	10.23	0.016				
194909	*G.Bacteroides*	10.23	0.016				
2137001	*G.Bacteroides*	10.43	0.016				
3940440	*G.Bacteroides*	10.43	0.016				
184753	*G.Bacteroides*	10.43	0.016				
161423	*G.Bacteroides*	10.55	0.017				
NCROTU3323	*G.Bacteroides*	10.55	0.017				
177150	*G.Bacteroides*	10.55	0.017				
1566189	*G.Bacteroides*	10.55	0.017				
3272632	*G.Bacteroides*	10.55	0.017				
190638	*G.Bacteroides*	10.55	0.017				
199716	*G.Bacteroides*	10.64	0.017				
844375	*G.Bacteroides*	10.64	0.017				
560336	*G.Bacteroides*	11.16	0.019				
4060124	*G.Bacteroides*	11.29	0.020				
302880	*G.Streptococcus*	28.70	0.022				
567972	*G.Streptococcus.S.agalactiae*	37.44	0.022				
3127555	*G.Bacteroides*	20.26	0.028				
470382	*G.Coprococcus*	25.75	0.029				
315429	*G.Bacteroides*	22.90	0.030				
2582263	*F.Enterobacteriaceae*	23.41	0.033				
1809696	*G.Bacteroides*	20.55	0.035				
1906483	*G.Bacteroides*	22.02	0.037				
NCROTU835	*F.Enterobacteriaceae*	24.48	0.038				
2283111	*G.Bacteroides.S.uniformis*	10.58	0.040				
3141094	*G.Bacteroides.S.ovatus*	3.49	0.048				
4381553	*G.Bacteroides*	3.49	0.048				
3304236	*G.Bacteroides*	3.49	0.048				
4455163	*G.Bacteroides*	3.49	0.048				
1129060	*G.Bacteroides*	3.49	0.048				
4447072	*G.Bacteroides.S.uniformis*	3.49	0.048				
2740953	*G.Bacteroides*	3.49	0.048				
861807	*G.Corynebacterium*	33.42	0.054				
975306	*G.Roseburia.S.faecis*	28.74	0.065				
270094	*G.Bacteroides*	20.54	0.071				
NCROTU1208	*G.Streptococcus*	17.14	0.071				
NROTU20	*F.Lachnospiraceae*	22.37	0.071				
1076969	*G.Streptococcus*	25.11	0.084				
875735	*G.Actinomyces*	22.35	0.085				
1108275	*G.Comamonas*	22.32	0.087				
1055824	*G.Staphylococcus*	21.18	0.093				

*NROTU = New.ReferenceOTU

**NCROTU = New.CleanUp.ReferenceOTU

***O., *F.*, *G.*, and *S.* in taxonomy labels indicate that the level of taxonomy is order, family, genus, or species.

Table S5i. Relation of maternal nut, legume, and soy intake with infant stool microbial OTUs, in infants delivered by cesarean (n = 48)

Positive Associations				Negative Associations			
**OTU**	**Taxonomy*** **	**Coefficient**	**p-value**	**OTU**	**Taxonomy*** **	**Coefficient**	**p-value**
86428	*G.Veillonella.S.dispar*	6.92	7.1E-04	851865	*G.Faecalibacterium.S.prausnitzii*	-5.41	8.7E-03
878104	*G.Veillonella.S.dispar*	5.48	1.5E-03	289709	*G.Escherichia.S.coli*	-6.22	0.010
962249	*G.Veillonella.S.dispar*	4.68	2.9E-03	701221	*G.Roseburia*	-4.92	0.011
4318671	*G.Veillonella.S.dispar*	5.27	3.1E-03	231787	*F.Enterobacteriaceae*	-6.09	0.011
148620	*F.Enterobacteriaceae*	2.94	4.2E-03	1067519	*G.Staphylococcus*	-4.41	0.014
4359220	*G.Veillonella.S.dispar*	4.46	5.5E-03	1109247	*F.Enterobacteriaceae*	-6.34	0.014
526583	*F.Clostridiaceae*	3.97	0.014	1108656	*F.Enterobacteriaceae*	-5.25	0.017
4478358	*G.Veillonella.S.dispar*	4.90	0.017	369027	*F.Lachnospiraceae*	-3.36	0.019
703635	*F.Enterobacteriaceae*	3.22	0.020	299267	*F.Enterobacteriaceae*	-5.40	0.020
315429	*G.Bacteroides*	3.00	0.028	3171486	*F.Enterobacteriaceae*	-4.23	0.021
4475758	*G.Veillonella.S.dispar*	4.43	0.028	173654	*F.Enterobacteriaceae*	-4.17	0.022
4371880	*G.Veillonella.S.dispar*	3.96	0.033	1028632	*G.Escherichia.S.coli*	-4.85	0.025
712047	*F.Clostridiaceae*	4.22	0.034	3483793	*F.Enterobacteriaceae*	-5.09	0.026
4334770	*G.Veillonella.S.dispar*	4.77	0.036	141145	*F.Enterobacteriaceae*	-5.36	0.028
524725	*G.Atopobium*	4.60	0.039	588216	*F.Enterobacteriaceae*	-5.18	0.030
1083508	*F.Xanthomonadaceae*	3.08	0.050	1111294	*G.Escherichia.S.coli*	-5.36	0.031
968675	*G.Haemophilus.S.parainfluenzae*	2.80	0.053	1649772	*G.Escherichia.S.coli*	-3.28	0.031
211191	*F.Ruminococcaceae*	2.40	0.053	114510	*F.Enterobacteriaceae*	-5.40	0.032
369555	*G.Ruminococcus*	2.11	0.054	917641	*G.Staphylococcus*	-3.86	0.040
3506872	*G.Veillonella.S.dispar*	3.75	0.054	538000	*F.Enterobacteriaceae*	-4.75	0.041
889025	*G.Acinetobacter*	3.27	0.056	782953	*F.Enterobacteriaceae*	-4.87	0.043
315982	*F.Clostridiaceae*	3.82	0.057	356760	*F.Erysipelotrichaceae*	-4.58	0.044
337909	*G.Clostridium*	3.78	0.057	667570	*F.Enterobacteriaceae*	-4.36	0.044
304779	*F.Clostridiaceae*	3.47	0.070	553611	*G.Bifidobacterium*	-4.79	0.050
285497	*F.Caulobacteraceae*	2.68	0.071	NROTU35	*G.Blautia.S.producta*	-3.55	0.052
152859	*F.Enterobacteriaceae*	2.82	0.075	4333897	*F.Enterobacteriaceae*	-4.29	0.053
198423	*G.[Ruminococcus].S.gnavus*	3.62	0.077	4457268	*F.Enterobacteriaceae*	-3.02	0.053
835771	*F.Enterobacteriaceae*	2.30	0.088	132041	*G.Bifidobacterium*	-2.91	0.056
4321400	*G.Streptococcus*	2.48	0.089	NROTU36	*F.Lachnospiraceae*	-2.91	0.058
316675	*F.Peptostreptococcaceae*	3.32	0.091	523140	*G.Ruminococcus*	-3.47	0.059
4192048	*G.Veillonella.S.dispar*	2.70	0.093	1085410	*G.Streptococcus*	-2.75	0.063
10085	*F.Enterobacteriaceae*	2.96	0.100	128382	*G.Dialister*	-3.92	0.065
				523589	*G.Clostridium.S.neonatale*	-2.62	0.066
				4376828	*G.Bifidobacterium*	-2.64	0.068
				339532	*G.Bifidobacterium*	-3.19	0.076
				996487	*G.Staphylococcus*	-3.59	0.078
				345362	*F.Enterobacteriaceae*	-3.51	0.081
				614083	*G.Staphylococcus*	-3.67	0.082
				4111715	*F.Enterobacteriaceae*	-3.65	0.086
				4303016	*G.Streptococcus*	-3.63	0.090
				304641	*G.Escherichia.S.coli*	-2.35	0.091
				3531225	*F.Enterobacteriaceae*	-4.12	0.091
				471180	*G.Bifidobacterium*	-4.20	0.093
				NCROTU835	*F.Enterobacteriaceae*	-2.57	0.095

*NROTU = New.ReferenceOTU

**NCROTU = New.CleanUp.ReferenceOTU

***O., *F.*, *G.*, and *S.* in taxonomy labels indicate that the level of taxonomy is order, family, genus, or species.

Table S5j. Relation of maternal PUFA intake with infant stool microbial OTUs, in infants delivered by cesarean (n = 48)

Positive Associations				Negative Associations			
**OTU**	**Taxonomy*** **	**Coefficient**	**p-value**	**OTU**	**Taxonomy*** **	**Coefficient**	**p-value**
4359220	*G.Veillonella.S.dispar*	1.47	5.0E-05	NROTU2	*F.Erysipelotrichaceae*	-1.46	5.1E-03
4328189	*F.Enterobacteriaceae*	1.08	4.8E-04	356760	*F.Erysipelotrichaceae*	-1.38	0.010
1696853	*G.Enterococcus*	0.89	3.7E-03	682726	*G.Eggerthella.S.lenta*	-1.27	0.014
298427	*G.Enterococcus*	1.01	6.3E-03	523140	*G.Ruminococcus*	-1.06	0.015
291508	*G.Enterococcus*	1.05	6.8E-03	173654	*F.Enterobacteriaceae*	-1.04	0.017
NCROTU2904	*G.Streptococcus*	0.90	8.9E-03	554338	*G.Blautia*	-1.18	0.024
878104	*G.Veillonella.S.dispar*	1.08	0.011	NCROTU1450	*F.Clostridiaceae*	-1.11	0.027
4318671	*G.Veillonella.S.dispar*	1.09	0.012	369027	*F.Lachnospiraceae*	-0.73	0.034
949789	*G.Enterococcus*	0.86	0.014	299267	*F.Enterobacteriaceae*	-1.10	0.051
577710	*G.Blautia.S.producta*	0.83	0.014	780650	*F.Clostridiaceae*	-1.18	0.052
3506872	*G.Veillonella.S.dispar*	1.10	0.017	716006	*G.Lactococcus*	-0.83	0.058
4371880	*G.Veillonella.S.dispar*	1.03	0.020	520369	*F.Clostridiaceae*	-1.10	0.060
524292	*G.Staphylococcus*	1.07	0.024	523589	*G.Clostridium.S.neonatale*	-0.63	0.063
743120	*F.Enterobacteriaceae*	0.61	0.024	198788	*G.Bacteroides*	-0.48	0.066
322798	*F.Clostridiaceae*	1.10	0.026	336710	*G.Bacteroides*	-0.48	0.066
4192048	*G.Veillonella.S.dispar*	0.83	0.028	553611	*G.Bifidobacterium*	-1.06	0.072
841907	*G.Bilophila*	0.37	0.029	72820	*G.Bifidobacterium*	-0.60	0.072
888300	*G.Streptococcus*	0.97	0.030	3663794	*G.Lactobacillus*	-0.54	0.078
292364	*G.Enterococcus*	0.78	0.034	1000592	*G.Anaerococcus*	-0.94	0.079
4388645	*G.Enterococcus*	1.01	0.035	1078587	*G.Blautia*	-0.75	0.082
10085	*F.Enterobacteriaceae*	0.88	0.039	4303016	*G.Streptococcus*	-0.89	0.082
701864	*G.Enterococcus*	0.88	0.042	169182	*F.Enterobacteriaceae*	-0.62	0.085
593672	*G.Enterococcus*	0.76	0.042	588216	*F.Enterobacteriaceae*	-0.99	0.088
4433947	*G.Bacteroides*	0.41	0.046	555945	*F.Peptostreptococcaceae*	-0.70	0.088
189403	*G.[Ruminococcus].S.gnavus*	0.40	0.046	604966	*G.Lactobacillus*	-0.65	0.091
892845	*G.Enterococcus*	0.81	0.049	4376828	*G.Bifidobacterium*	-0.59	0.091
113773	*G.Enterococcus*	1.02	0.051				
4475758	*G.Veillonella.S.dispar*	0.94	0.052				
103166	*F.Enterobacteriaceae*	0.56	0.054				
17976	*G.Enterococcus*	0.68	0.056				
2656868	*G.Bacteroides*	0.45	0.057				
810399	*G.Enterococcus*	0.83	0.064				
365181	*G.Collinsella.S.aerofaciens*	0.59	0.064				
198449	*G.Bacteroides.S.caccae*	0.88	0.065				
4453060	*G.Enterococcus*	0.95	0.065				
198423	*G.[Ruminococcus].S.gnavus*	0.88	0.072				
703635	*F.Enterobacteriaceae*	0.60	0.073				
NCROTU3436	*G.Staphylococcus*	0.82	0.075				
4478358	*G.Veillonella.S.dispar*	0.89	0.077				
234488	*G.Bacteroides*	0.26	0.080				
4334711	*G.Bacteroides*	0.25	0.081				
3745352	*G.Bacteroides*	0.25	0.081				
925707	*G.Streptococcus*	0.65	0.083				
100039	*G.Enterococcus*	0.69	0.091				
226338	*G.Enterococcus*	0.77	0.091				
1095073	*G.Propionibacterium.S.acnes*	0.61	0.093				
521851	*G.Enterococcus*	0.87	0.094				
996487	*G.Staphylococcus*	0.82	0.094				
1116674	*F.Enterobacteriaceae*	0.71	0.098				

*NROTU = New.ReferenceOTU

**NCROTU = New.CleanUp.ReferenceOTU

***O., *F.*, *G.*, and *S.* in taxonomy labels indicate that the level of taxonomy is order, family, genus, or species.

Table S5k. Relation of maternal vegetable intake with infant stool microbial OTUs, in infants delivered by cesarean (n = 48)

Positive Associations				Negative Associations			
**OTU**	**Taxonomy*****	**Coefficient**	**p-value**	**OTU**	**Taxonomy*****	**Coefficient**	**p-value**
470382	*G.Coprococcus*	2.11	8.8E-03	4472685	*G.Streptococcus*	-3.95	2.3E-04
555945	*F.Peptostreptococcaceae*	2.21	0.014	513500	*G.Streptococcus*	-3.10	6.0E-03
554338	*G.Blautia*	2.83	0.015	4326406	*G.Streptococcus*	-1.90	0.024
1097359	*G.Acinetobacter*	1.51	0.017	1047077	*G.Actinomyces*	-2.35	0.027
1055212	*G.Enterococcus*	2.12	0.017	1064036	*G.Peptoniphilus*	-2.17	0.030
226338	*G.Enterococcus*	2.30	0.022	925707	*G.Streptococcus*	-1.80	0.031
686789	*F.Enterococcaceae*	1.96	0.024	1085410	*G.Streptococcus*	-1.64	0.038
1696853	*G.Enterococcus*	1.58	0.025	4441855	*G.Streptococcus*	-2.50	0.040
17976	*G.Enterococcus*	1.70	0.033	898871	*G.Staphylococcus*	-1.75	0.045
NROTU24	*G.Enterococcus*	2.10	0.037	183651	*G.Blautia*	-1.66	0.051
949863	*G.Lactobacillus.S.zeae*	2.37	0.037	871442	*G.Streptococcus*	-1.43	0.064
511378	*G.Veillonella*	1.85	0.048	3472078	*G.Bacteroides.S.fragilis*	-0.97	0.081
701864	*G.Enterococcus*	1.88	0.053	3507744	*G.Bacteroides.S.fragilis*	-0.96	0.081
1065974	*G.Enterococcus*	2.06	0.060	4377091	*G.Bacteroides.S.fragilis*	-0.94	0.081
15257	*G.Enterococcus*	2.22	0.063	972033	*G.Streptococcus*	-2.01	0.082
996487	*G.Staphylococcus*	2.02	0.065	4345821	*G.Bacteroides.S.fragilis*	-0.93	0.082
17309	*G.Lactobacillus*	1.70	0.066	4356331	*G.Bacteroides.S.fragilis*	-0.92	0.082
365484	*O.Clostridiales*	1.60	0.067	4456852	*G.Bacteroides.S.fragilis*	-0.92	0.082
4333020	*F.Enterobacteriaceae*	1.98	0.073	1078207	*G.Streptococcus*	-1.39	0.082
NCROTU3436	*G.Staphylococcus*	1.85	0.074	2944933	*G.Bacteroides.S.fragilis*	-0.90	0.083
132661	*G.Enterococcus*	1.98	0.081	184567	*G.Bacteroides.S.fragilis*	-0.89	0.083
NROTU35	*G.Blautia.S.producta*	1.71	0.084	92535	*G.Streptococcus*	-2.07	0.083
759061	*F.Enterobacteriaceae*	2.10	0.086	4329112	*G.Bacteroides.S.fragilis*	-0.90	0.083
696563	*G.Blautia.S.producta*	1.87	0.086	4479397	*G.Bacteroides.S.fragilis*	-0.89	0.083
334656	*G.Enterococcus*	1.34	0.090	2636449	*G.Bacteroides.S.fragilis*	-0.87	0.084
2026051	*F.Enterococcaceae*	1.42	0.096	302683	*G.Blautia*	-1.28	0.087
975306	*G.Roseburia.S.faecis*	1.80	0.098	339599	*G.Bacteroides.S.fragilis*	-1.06	0.093
572843	*G.Enterococcus*	2.03	0.102	920226	*G.Streptococcus*	-1.70	0.096
1111582	*G.Enterococcus*	0.69	0.104	941096	*G.Streptococcus*	-1.80	0.100
593781	*G.Enterococcus*	1.52	0.108	536866	*G.Streptococcus*	-1.98	0.102
356733	*G.Staphylococcus*	1.44	0.109	342380	*G.Blautia*	-1.27	0.109
512239	*G.Enterococcus*	1.90	0.109	86428	*G.Veillonella.S.dispar*	-1.87	0.109
4473975	*G.Enterococcus*	1.10	0.110	350277	*G.Bacteroides.S.uniformis*	-1.47	0.111
4349891	*G.Lactobacillus*	1.39	0.112	187035	*G.Blautia*	-1.30	0.116
2575651	*G.[Ruminococcus].S.gnavus*	1.05	0.113	176775	*G.Phascolarctobacterium*	-1.21	0.120
2724175	*G.[Ruminococcus].S.gnavus*	0.74	0.118	134265	*G.Prevotella*	-0.59	0.121
292364	*G.Enterococcus*	1.30	0.118	4462083	*G.Streptococcus*	-1.17	0.132
113773	*G.Enterococcus*	1.83	0.123				
737912	*F.Enterobacteriaceae*	1.14	0.125				
892845	*G.Enterococcus*	1.42	0.126				
703741	*G.Lactobacillus*	0.98	0.127				
810399	*G.Enterococcus*	1.51	0.135				
364034	*F.Lachnospiraceae*	1.55	0.142				
641490	*G.Enterococcus*	1.17	0.146				
701221	*G.Roseburia*	1.54	0.149				

*NROTU = New.ReferenceOTU

**NCROTU = New.CleanUp.ReferenceOTU

***O., *F.*, *G.*, and *S.* in taxonomy labels indicate that the level of taxonomy is order, family, genus, or species.

Table S5l. Relation of maternal whole grain intake with infant stool microbial OTUs, in infants delivered by cesarean (n = 48)

Positive Associations				Negative Associations			
**OTU**	**Taxonomy*** **	**Coefficient**	**p-value**	**OTU**	**Taxonomy*** **	**Coefficient**	**p-value**
814442	*F.Enterobacteriaceae*	5.03	4.2E-04	1109247	*F.Enterobacteriaceae*	-10.26	2.0E-03
4318990	*F.Enterobacteriaceae*	6.21	2.0E-03	141145	*F.Enterobacteriaceae*	-8.65	6.3E-03
176704	*G.[Ruminococcus].S.gnavus*	3.87	2.9E-03	114510	*F.Enterobacteriaceae*	-8.84	6.8E-03
4476604	*O.Clostridiales*	3.70	3.2E-03	289709	*G.Escherichia.S.coli*	-8.30	8.7E-03
258785	*F.Enterobacteriaceae*	5.82	7.2E-03	3483793	*F.Enterobacteriaceae*	-7.49	0.012
2575651	*G.[Ruminococcus].S.gnavus*	4.17	8.0E-03	1108656	*F.Enterobacteriaceae*	-7.18	0.013
703635	*F.Enterobacteriaceae*	4.79	8.0E-03	548587	*G.[Eubacterium].S.dolichum*	-6.67	0.013
331575	*G.[Ruminococcus].S.gnavus*	4.02	8.0E-03	516814	*G.Streptococcus*	-2.47	0.020
963779	*G.Agrobacterium*	2.99	0.010	1111294	*G.Escherichia.S.coli*	-7.43	0.023
523589	*G.Clostridium.S.neonatale*	4.68	0.011	4333897	*F.Enterobacteriaceae*	-6.59	0.023
3044876	*G.[Ruminococcus].S.gnavus*	4.74	0.012	128382	*G.Dialister*	-6.03	0.030
2683271	*G.[Ruminococcus].S.gnavus*	4.55	0.012	193466	*G.Blautia*	-4.79	0.031
1551841	*G.[Ruminococcus].S.gnavus*	4.30	0.014	538000	*F.Enterobacteriaceae*	-6.45	0.035
NROTU17	*G.[Ruminococcus].S.gnavus*	5.96	0.019	4111715	*F.Enterobacteriaceae*	-5.83	0.036
189403	*G.[Ruminococcus].S.gnavus*	2.48	0.023	3171486	*F.Enterobacteriaceae*	-5.00	0.040
290849	*F.Enterobacteriaceae*	4.00	0.028	231787	*F.Enterobacteriaceae*	-6.36	0.047
380567	*G.Corynebacterium*	4.24	0.029	573270	*O.Burkholderiales*	-2.71	0.049
656517	*F.Enterobacteriaceae*	4.89	0.032	782953	*F.Enterobacteriaceae*	-6.22	0.049
835771	*F.Enterobacteriaceae*	3.75	0.032	196176	*G.Dorea*	-6.09	0.059
164789	*F.Enterobacteriaceae*	4.53	0.033	326662	*G.Bacteroides.S.uniformis*	-2.63	0.061
4385577	*F.Lachnospiraceae*	4.61	0.036	183651	*G.Blautia*	-3.83	0.067
3376513	*G.[Ruminococcus].S.gnavus*	4.04	0.037	696563	*G.Blautia.S.producta*	-4.87	0.067
362767	*F.Lachnospiraceae*	5.96	0.044	797229	*F.Enterobacteriaceae*	-2.80	0.072
288442	*G.[Ruminococcus].S.gnavus*	3.90	0.044	173744	*G.Megasphaera*	-2.79	0.074
813457	*F.Enterobacteriaceae*	4.11	0.046	360238	*F.Erysipelotrichaceae*	-3.02	0.074
3946926	*G.Lactobacillus*	3.78	0.046	442743	*F.Enterobacteriaceae*	-3.56	0.075
184729	*F.Lachnospiraceae*	2.82	0.048	588216	*F.Enterobacteriaceae*	-5.63	0.076
2582263	*F.Enterobacteriaceae*	3.65	0.052	302683	*G.Blautia*	-3.20	0.081
191999	*F.Lachnospiraceae*	2.64	0.053	3531225	*F.Enterobacteriaceae*	-5.56	0.084
1839271	*G.[Ruminococcus].S.gnavus*	3.25	0.053	189971	*G.Blautia*	-3.45	0.085
1654474	*G.[Ruminococcus].S.gnavus*	2.76	0.055	3745352	*G.Bacteroides*	-1.31	0.090
4345397	*F.Enterobacteriaceae*	2.83	0.057	4334711	*G.Bacteroides*	-1.35	0.091
1052663	*G.Staphylococcus*	3.89	0.058	234488	*G.Bacteroides*	-1.40	0.091
NCROTU2526	*F.Enterobacteriaceae*	2.33	0.059	173654	*F.Enterobacteriaceae*	-4.09	0.094
2724175	*G.[Ruminococcus].S.gnavus*	2.18	0.059				
4426874	*G.[Ruminococcus].S.gnavus*	3.05	0.062				
360015	*G.[Ruminococcus].S.gnavus*	4.78	0.068				
NCROTU586	*F.Enterobacteriaceae*	2.73	0.071				
4408758	*G.Veillonella.S.dispar*	4.48	0.071				
191251	*G.Parabacteroides.S.distasonis*	4.52	0.074				
192342	*F.Enterobacteriaceae*	4.16	0.077				
861807	*G.Corynebacterium*	5.23	0.077				
NCROTU3436	*G.Staphylococcus*	4.46	0.080				
182517	*G.[Ruminococcus].S.gnavus*	3.27	0.082				
103166	*F.Enterobacteriaceae*	2.76	0.083				
316132	*G.Bacteroides*	2.58	0.085				
949863	*G.Lactobacillus.S.zeae*	4.82	0.087				
917641	*G.Staphylococcus*	4.28	0.087				
149034	*F.Enterobacteriaceae*	3.51	0.087				
336632	*G.Akkermansia.S.muciniphila*	2.91	0.092				
NROTU15	*G.Streptococcus*	3.48	0.095				

*NROTU = New.ReferenceOTU

**NCROTU = New.CleanUp.ReferenceOTU

***O., *F.*, *G.*, and *S.* in taxonomy labels indicate that the level of taxonomy is order, family, genus, or species.

Figure S2. Participant Flow Chart

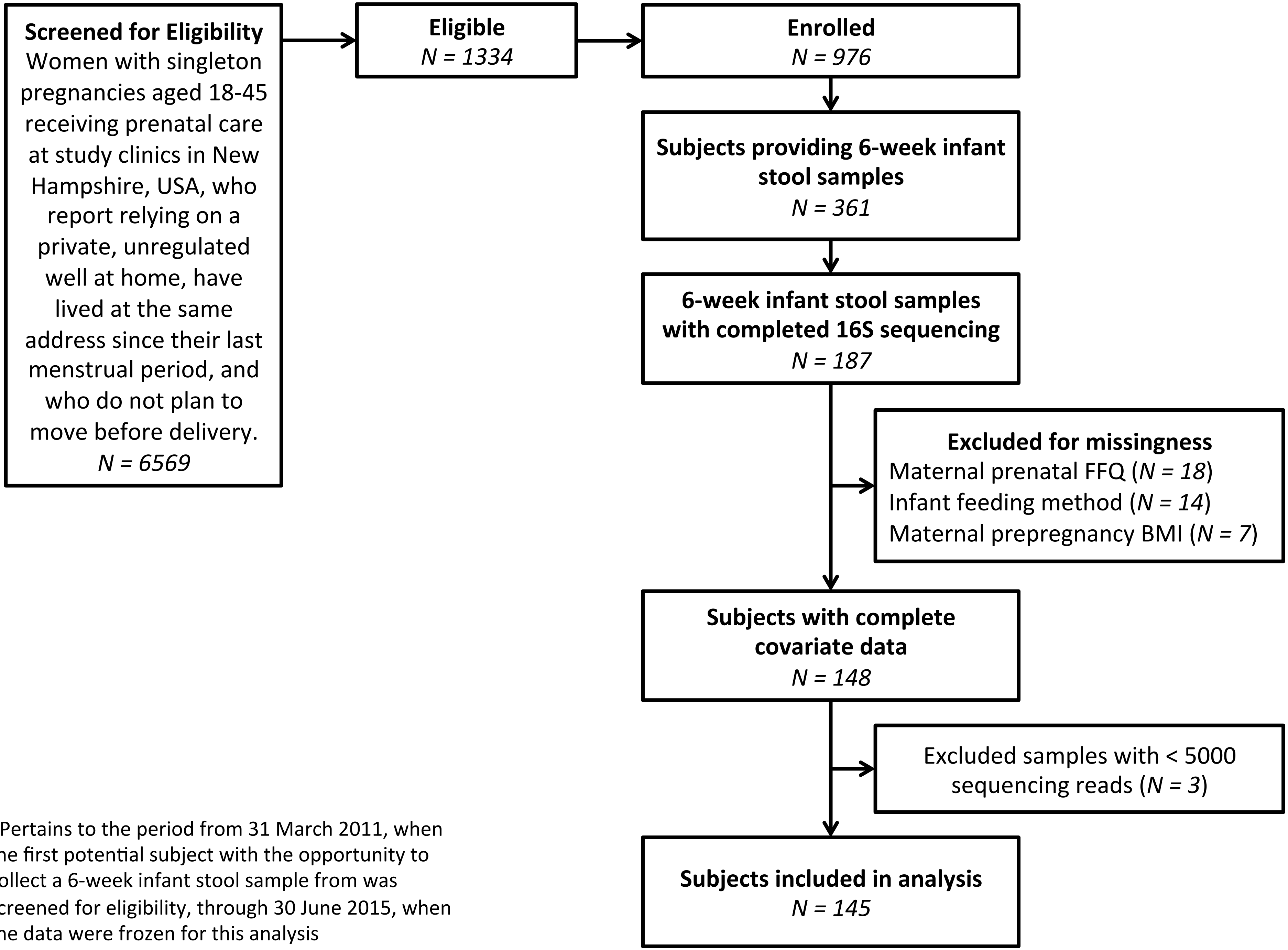

*Pertains to the period from 31 March 2011, when the first potential subject with the opportunity to collect a 6-week infant stool sample from was screened for eligibility, through 30 June 2015, when the data were frozen for this analysis

Table S6. Comparison of infant gut microbiome types in all versus full term (n = 137) infants

		Sensitivity analysis, without premature infants		
		IGMT 1	*IGMT 2*	*IGMT 3*
Original analysis	*IGMT 1*	*39*	*0*	*0*
	IGMT 2	*0*	*42*	*0*
	IGMT 3	*0*	*1*	*55*

Figure S3. Infant gut microbiota relative abundances restricted to full-term infants.

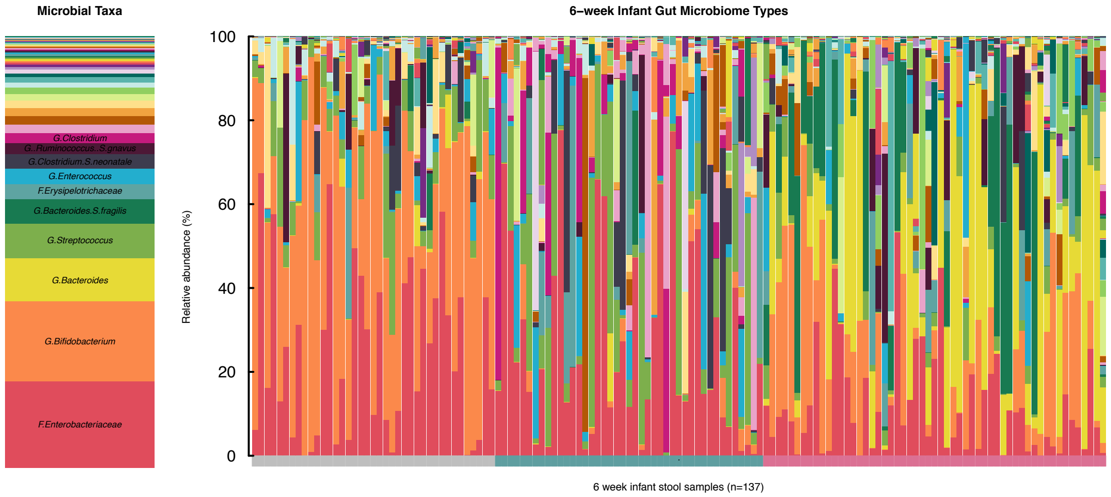

PAM clustering of all (n = 137) infants results in the same three clusters or infant gut microbiome types (IGMT) with high concordance after removing all infants delivered prior to 37 weeks of gestation.

Table S7. Comparison of infant gut microbiome types in the vaginal delivery group in all versus full term (n = 92) infants

		Sensitivity analysis, without premature infants		
		IGMT 1	*IGMT 2*	*IGMT 3*
Original analysis	*IGMT 1*	*23*	*0*	*0*
	IGMT 2	*0*	*19*	*0*
	IGMT 3	*0*	*0*	*50*

Figure S4. Infant gut microbiota relative abundances restricted to full-term vaginally delivered infants.

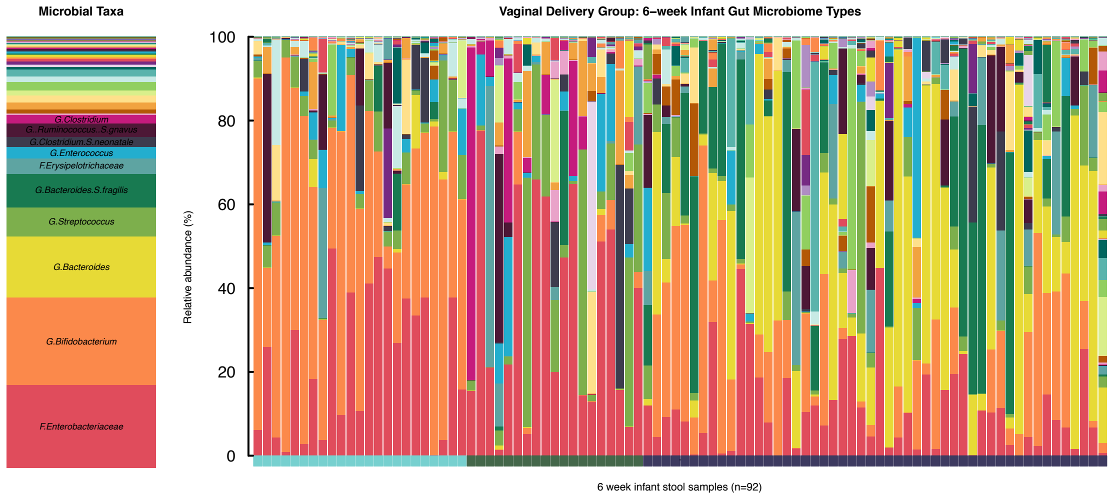

PAM clustering of vaginally delivered infants (n = 92) results in the same three clusters, with perfect concordance.

Table S8. Comparison of infant gut microbiome types in infants delivered by Cesarean section in all versus full term (n = 45) infants

		Sensitivity analysis, without premature infants	
		IGMT 1	*IGMT 2*
Original analysis	*IGMT 1*	*14*	*0*
	IGMT 2	*1*	*10*
	IGMT 3	*0*	*20*

Figure S5. Infant gut microbiota relative abundances restricted to full-term infants delivered by Cesarean Section.

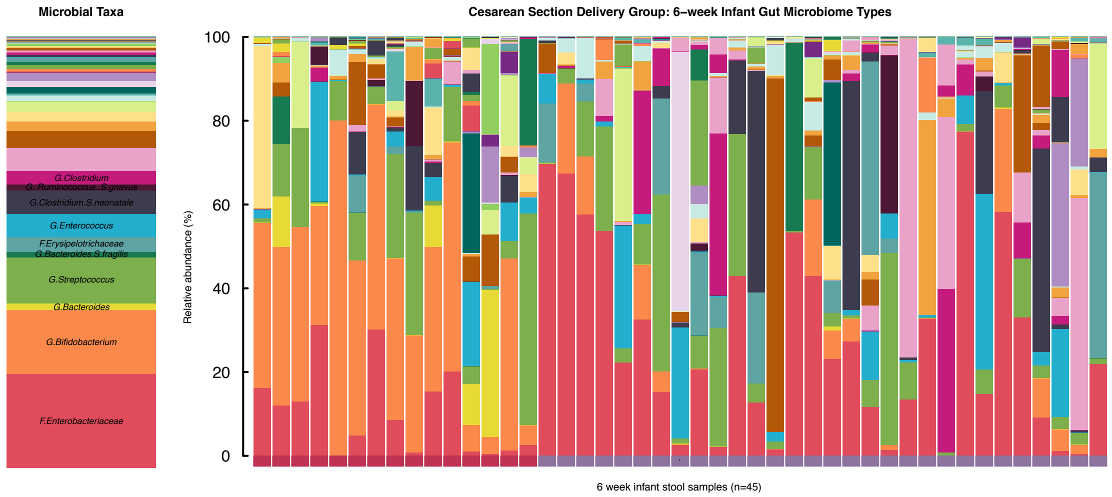

PAM clustering of infants delivered by Cesarean section (n = 45) results in two clusters (instead of three), with high concordance – the 2nd and 3rd clusters combine.

Multinomial logistic regression models testing the relationship between maternal dietary factors as continuous measures and infant gut microbiome type do not qualitatively change after excluding premature infants. Maternal fruit intake is associated with higher odds of vaginally delivered infants clustering to the second cluster (OR (95% CI) = 2.84 (1.39,5.79)) or the third cluster (OR (95% CI) = 1.72 (1.00, 294)) compared to the first cluster. Higher maternal dairy intake is positively associated with the membership in the second compared to the first cluster in infants delivered by Cesarean section (OR (95% CI) = 2.14 (1.12, 4.07)). All estimates are adjusted for infant feeding method, maternal BMI, parity, and sequencing batch.

Table S9. Maternal diet is related to infant gut microbiome cluster membership according to delivery mode, excluding infants born before 37 weeks gestation

Dietary Factor	Vaginal (n = 92)[2]		Cesarean (n = 45)[2]
	Cluster 2	Cluster 3	Cluster 2
aMED score	1.34 (0.91,1.96)	0.98 (0.74,1.31)	1.00 (0.64,1.55)
Dairy	0.83 (0.52,1.34)	0.78 (0.54,1.12)	2.14 (1.12,4.07)
Fruit	2.84 (1.39,5.79)	1.72 (1.00,2.94)	0.61 (0.30,1.24)
Vegetables	0.85 (0.57,1.26)	0.80 (0.58,1.10)	0.92 (0.60,1.40)
Whole Grains	0.99 (0.38,2.60)	1.12 (0.57,2.19)	1.43 (0.47,4.36)
Fish and Seafood	1.18 (0.02,71.83)	1.18 (0.04,36.48)	0.19 (0.00,13.46)
Nuts, Legumes, and Soy	0.57 (0.21,1.51)	0.48 (0.22,1.02)	1.02 (0.47,2.23)
Red and Processed Meat	3.55 (0.64,19.64)	2.07 (0.52,8.13)	0.79 (0.14,4.31)
Polyunsaturated fat	0.85 (0.65,1.11)	0.87 (0.72,1.07)	1.12 (0.91,1.38)
EPA	0.17 (0.00,75.67)	2.94 (0.05,182.12)	0.02 (0.00,10.66)
DHA	0.18 (0.00,95.32)	3.12 (0.04,227.22)	0.18 (0.00,52.67)
MUFA:SFA ratio	4.40 (0.30,64.01)	3.65 (0.41,32.49)	1.19 (0.13,10.68)

[1]Cluster 1 is the reference in all models

[2]All estimates are adjusted for infant feeding method, maternal BMI, parity, and batch

Figure S6. Predicted probability of cluster membership by maternal diet, excluding infants born before 37 weeks gestation

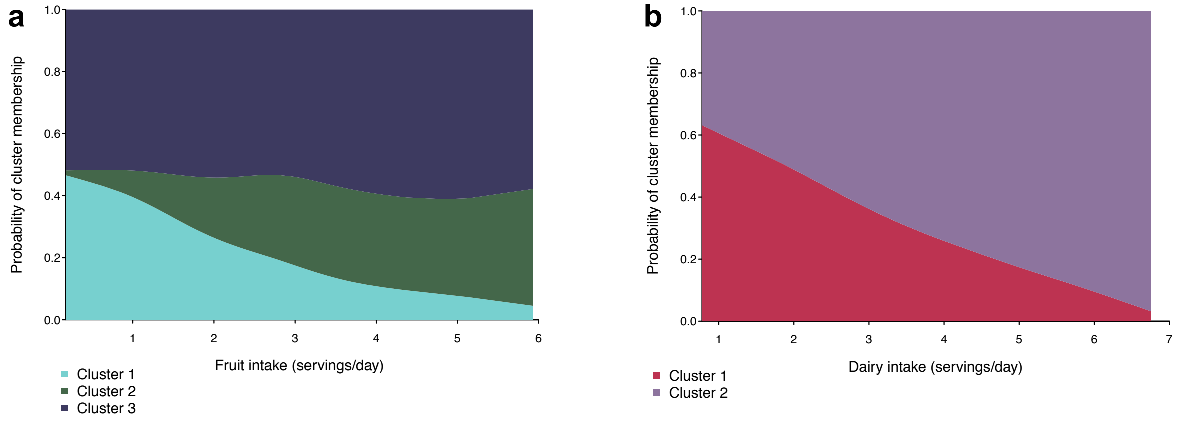

Predicted probability plot of infant stool cluster membership (within delivery mode groups) by maternal (a) fruit intake during pregnancy in infants delivered vaginally (n = 92) and (b) dairy intake during pregnancy in infants delivered by Cesarean section (n = 45) from multinomial logistic regression models adjusted for infant feeding method, maternal BMI, parity, and batch. Cluster 1 is the reference group in both models.

Table S10. Relation of microbial community composition in six week old infants stratified by delivery mode, excluding infants born before 37 weeks gestation

	p-value[1, 2]	
**Dietary Factor**	**Vaginal delivery (n = 92)**	**Cesarean section (n = 45)**
aMED Score	0.16	0.69
Dairy	0.45	0.049
Fruit	0.030	0.80
Vegetables	0.51	0.94
Whole Grains	0.35	0.83
Fish and Seafood	0.28	0.76
Nuts, Legumes, and Soy	0.50	0.51
Red and Processed Meat	0.84	0.94
Polyunsaturated Fat	0.85	0.35
EPA	0.28	0.59
DHA	0.35	0.82
MUFA:SFA Ratio	0.33	0.37

[1]All *p*-values are determined by PERMANOVA
[2]*p*-values are adjusted for infant feeding method, maternal BMI, parity, and batch

Figure S7. Maternal dietary factors in relation to overall microbial community structure, excluding infants delivered before 37 weeks gestation

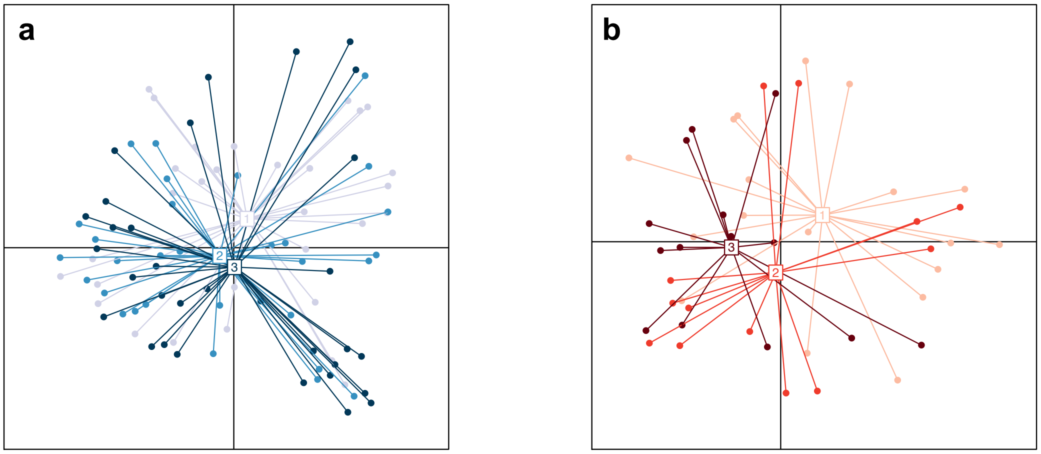

PCoA plots showing generalized UniFrac distances for (a) vaginally delivered infants, colored by maternal fruit intake tertiles (PERMANOVA *p*-value = 0.030 for maternal fruit intake as a continuous variable) and (b) Cesarean delivered infants, colored by maternal dairy intake tertiles (PERMANOVA p = 0.049 for maternal dairy intake as a continuous variable). Samples are shown as points, where points closer together have more similar microbial communities and points farther apart have dissimilar microbial communities.

Table S11a. Relation of maternal aMED score with infant stool microbial OTUs, in infants delivered vaginally (n = 92)

Positive Associations				Negative Associations			
**OTU**	**Taxonomy*** **	**Coefficient**	**p-value**	**OTU**	**Taxonomy*** **	**Coefficient**	**p-value**
92535	*G.Streptococcus*	1.93	1.6E-03	4447072	*G.Bacteroides.S.uniformis*	-1.35	6.6E-03
NCROTU2526	*F.Enterobacteriaceae*	1.28	2.5E-03	523589	*G.Clostridium.S.neonatale*	-1.95	8.9E-03
228556	*F.Enterobacteriaceae*	1.94	3.3E-03	231787	*F.Enterobacteriaceae*	-1.78	0.012
4318990	*F.Enterobacteriaceae*	1.52	3.6E-03	975306	*G.Roseburia.S.faecis*	-1.45	0.015
920226	*G.Streptococcus*	1.47	5.2E-03	797229	*F.Enterobacteriaceae*	-0.90	0.015
737912	*F.Enterobacteriaceae*	1.39	7.5E-03	1109247	*F.Enterobacteriaceae*	-1.75	0.016
801438	*F.Enterobacteriaceae*	1.76	7.8E-03	562376	*G.Dorea*	-1.37	0.017
233220	*F.Enterobacteriaceae*	1.86	9.4E-03	NROTU7	*G.Coprococcus*	-1.05	0.018
1063759	*G.Corynebacterium*	1.50	9.8E-03	197072	*G.Bacteroides.S.uniformis*	-1.34	0.018
3908638	*F.Enterobacteriaceae*	1.79	0.011	114510	*F.Enterobacteriaceae*	-1.71	0.018
681779	*F.Enterobacteriaceae*	1.43	0.011	4310208	*G.Veillonella*	-1.17	0.019
4328189	*F.Enterobacteriaceae*	1.22	0.011	344154	*G.Bacteroides.S.uniformis*	-1.27	0.022
688934	*F.Enterobacteriaceae*	1.74	0.011	1111294	*G.Escherichia.S.coli*	-1.70	0.023
NROTU27	*F.Enterobacteriaceae*	1.05	0.012	289709	*G.Escherichia.S.coli*	-1.62	0.024
821080	*F.Enterobacteriaceae*	1.79	0.013	3376513	*G.[Ruminococcus].S.gnavus*	-1.20	0.025
345540	*F.Enterobacteriaceae*	1.39	0.015	3171486	*F.Enterobacteriaceae*	-1.47	0.026
888300	*G.Streptococcus*	1.34	0.015	3483793	*F.Enterobacteriaceae*	-1.59	0.027
152859	*F.Enterobacteriaceae*	1.27	0.017	141145	*F.Enterobacteriaceae*	-1.57	0.027
1116674	*F.Enterobacteriaceae*	1.37	0.018	NCROTU2904	*G.Streptococcus*	-0.85	0.029
746679	*F.Enterobacteriaceae*	1.60	0.018	4426874	*G.[Ruminococcus].S.gnavus*	-1.14	0.029
686972	*F.Enterobacteriaceae*	1.64	0.018	NROTU36	*F.Lachnospiraceae*	-0.96	0.030
1123414	*F.Enterobacteriaceae*	1.48	0.019	588216	*F.Enterobacteriaceae*	-1.54	0.030
236821	*F.Enterobacteriaceae*	1.65	0.019	356760	*F.Erysipelotrichaceae*	-1.47	0.033
1083508	*F.Xanthomonadaceae*	1.06	0.019	331575	*G.[Ruminococcus].S.gnavus*	-1.12	0.035
241415	*F.Enterobacteriaceae*	1.64	0.022	336012	*G.Bacteroides.S.uniformis*	-1.00	0.036
2529285	*F.Enterobacteriaceae*	1.57	0.027	4473977	*F.Lachnospiraceae*	-0.84	0.037
258785	*F.Enterobacteriaceae*	1.05	0.028	217734	*G.Streptococcus.S.anginosus*	-1.27	0.038
315982	*F.Clostridiaceae*	0.97	0.028	4452632	*G.Clostridium.S.butyricum*	-1.03	0.042
322798	*F.Clostridiaceae*	1.06	0.032	1839271	*G.[Ruminococcus].S.gnavus*	-1.17	0.046
4376230	*F.Enterobacteriaceae*	1.55	0.034	646549	*G.Pseudomonas*	-0.94	0.049
819999	*F.Enterobacteriaceae*	1.55	0.036	1028632	*G.Escherichia.S.coli*	-1.36	0.051
668514	*F.Enterobacteriaceae*	1.44	0.037	4385577	*F.Lachnospiraceae*	-1.20	0.052
518002	*F.Enterobacteriaceae*	1.39	0.040	1551841	*G.[Ruminococcus].S.gnavus*	-1.08	0.052
582691	*F.Clostridiaceae*	0.96	0.044	2283111	*G.Bacteroides.S.uniformis*	-1.05	0.057
425721	*F.Enterobacteriaceae*	1.14	0.046	2683271	*G.[Ruminococcus].S.gnavus*	-1.11	0.058
759061	*F.Enterobacteriaceae*	1.31	0.047	503315	*G.Finegoldia*	-1.31	0.061
780650	*F.Clostridiaceae*	1.33	0.050	211191	*F.Ruminococcaceae*	-0.83	0.062
232696	*F.Enterobacteriaceae*	1.25	0.051	529979	*F.Erysipelotrichaceae*	-0.96	0.064
3506872	*G.Veillonella.S.dispar*	1.11	0.051	362997	*G.Bacteroides*	-0.99	0.066
210269	*F.Enterobacteriaceae*	1.40	0.053	1654474	*G.[Ruminococcus].S.gnavus*	-1.09	0.071
15366	*G.Streptococcus*	1.28	0.054	1033018	*G.Janthinobacterium.S.lividum*	-0.75	0.072
NCROTU586	*F.Enterobacteriaceae*	0.86	0.059	176704	*G.[Ruminococcus].S.gnavus*	-0.94	0.078
813457	*F.Enterobacteriaceae*	0.95	0.067	589071	*G.Bacteroides.S.uniformis*	-1.22	0.078
703635	*F.Enterobacteriaceae*	0.91	0.069	3531225	*F.Enterobacteriaceae*	-1.27	0.080
972033	*G.Streptococcus*	1.14	0.070	320395	*G.Bacteroides.S.uniformis*	-1.00	0.081
754778	*F.Enterobacteriaceae*	1.23	0.072	288442	*G.[Ruminococcus].S.gnavus*	-1.06	0.081
516814	*G.Streptococcus*	0.73	0.075	254938	*F.Oxalobacteraceae*	-0.66	0.082
203579	*F.Enterobacteriaceae*	1.19	0.077	332588	*G.Bacteroides.S.uniformis*	-0.86	0.083
4441855	*G.Streptococcus*	1.16	0.077	352304	*F.Lachnospiraceae*	-1.06	0.084
2647328	*G.Haemophilus.S.parainfluenzae*	0.60	0.077	102049	*G.Bifidobacterium*	-0.81	0.085
337909	*G.Clostridium*	0.88	0.077	299267	*F.Enterobacteriaceae*	-1.19	0.085
579608	*G.Streptococcus*	0.98	0.080	328617	*G.Bacteroides.S.uniformis*	-0.83	0.085
10085	*F.Enterobacteriaceae*	0.90	0.087	4333897	*F.Enterobacteriaceae*	-1.24	0.086
466445	*F.Enterobacteriaceae*	0.79	0.088	NCROTU4975	*G.Bacteroides.S.uniformis*	-1.07	0.087
355471	*F.Clostridiaceae*	0.84	0.091	1108656	*F.Enterobacteriaceae*	-1.23	0.087
4333020	*F.Enterobacteriaceae*	1.07	0.091	189403	*G.[Ruminococcus].S.gnavus*	-0.81	0.089
3228974	*F.Enterobacteriaceae*	0.73	0.096	587530	*G.[Eubacterium].S.dolichum*	-0.70	0.098
				182517	*G.[Ruminococcus].S.gnavus*	-1.00	0.099

*NROTU = New.ReferenceOTU

**NCROTU = New.CleanUp.ReferenceOTU

***O., F., G., and S. in taxonomy labels indicate that the level of taxonomy is order, family, genus, or species.

Table S11b. Relation of maternal dairy intake with infant stool microbial OTUs, in infants delivered vaginally (n = 92)

Positive Associations				Negative Associations			
**OTU**	**Taxonomy*** **	**Coefficient**	**p-value**	**OTU**	**Taxonomy*** **	**Coefficient**	**p-value**
523589	*G.Clostridium.S.neonatale*	3.16	8.7E-04	NCROTU3325	*F.Enterobacteriaceae*	-1.68	6.4E-03
2202350	*G.Staphylococcus*	1.45	7.4E-03	NROTU20	*F.Lachnospiraceae*	-1.53	0.012
370183	*G.Blautia*	1.85	0.016	364034	*F.Lachnospiraceae*	-1.93	0.020
NCROTU4270	*G.Clostridium.S.neonatale*	1.26	0.016	572843	*G.Enterococcus*	-1.71	0.053
NCROTU2904	*G.Streptococcus*	1.19	0.017	1649772	*G.Escherichia.S.coli*	-1.42	0.055
806179	*G.Lactobacillus*	1.38	0.024	628226	*F.Peptostreptococcaceae*	-0.98	0.055
4294457	*G.Rothia.S.mucilaginosa*	1.82	0.027	4401450	*F.Enterobacteriaceae*	-1.33	0.056
503406	*G.Peptoniphilus*	1.80	0.029	851323	*G.Parabacteroides*	-1.87	0.059
217734	*G.Streptococcus.S.anginosus*	1.65	0.037	898871	*G.Staphylococcus*	-1.07	0.074
563086	*G.[Ruminococcus]*	1.13	0.037	342666	*F.Clostridiaceae*	-0.87	0.094
114821	*G.Veillonella*	1.89	0.038	712677	*O.Clostridiales*	-1.19	0.095
NCROTU3131	*G.Streptococcus*	1.23	0.045				
1108960	*G.Sphingomonas*	1.11	0.047				
176775	*G.Phascolarctobacterium*	1.54	0.047				
4352001	*G.Clostridium.S.neonatale*	1.04	0.051				
1084906	*G.Staphylococcus*	0.54	0.057				
4310208	*G.Veillonella*	1.18	0.067				
503315	*G.Finegoldia*	1.64	0.068				
359175	*F.Ruminococcaceae*	1.19	0.072				
4376828	*G.Bifidobacterium*	1.26	0.076				
137609	*G.Clostridium.S.neonatale*	1.09	0.078				
181239	*G.Bacteroides.S.uniformis*	1.06	0.081				
514272	*G.Coprococcus*	1.43	0.081				
183480	*G.Bacteroides*	1.17	0.090				
4452632	*G.Clostridium.S.butyricum*	1.11	0.091				
299267	*F.Enterobacteriaceae*	1.50	0.092				
814442	*F.Enterobacteriaceae*	1.12	0.093				

*NROTU = New.ReferenceOTU

**NCROTU = New.CleanUp.ReferenceOTU

****O*., *F*., *G*., and *S*. in taxonomy labels indicate that the level of taxonomy is order, family, genus, or species.

Table S11c. Relation of maternal fish and seafood intake with infant stool microbial OTUs, in infants delivered vaginally (n = 92)

Positive Associations				Negative Associations			
**OTU**	**Taxonomy*** **	**Coefficient**	**p-value**	**OTU**	**Taxonomy*** **	**Coefficient**	**p-value**
1098340	*G.Streptococcus*	19.22	3.2E-03	2876801	*G.Bacteroides.S.uniformis*	-20.076	3.6E-03
1027587	*G.Streptococcus*	16.95	3.6E-03	197072	*G.Bacteroides.S.uniformis*	-18.710	4.8E-03
754778	*F.Enterobacteriaceae*	22.43	5.0E-03	4447072	*G.Bacteroides.S.uniformis*	-16.514	5.1E-03
92535	*G.Streptococcus*	19.31	8.5E-03	326662	*G.Bacteroides.S.uniformis*	-17.250	7.4E-03
425721	*F.Enterobacteriaceae*	17.72	8.5E-03	348027	*G.Bacteroides.S.uniformis*	-15.993	7.5E-03
821080	*F.Enterobacteriaceae*	20.52	0.016	2283111	*G.Bacteroides.S.uniformis*	-16.944	8.5E-03
274754	*F.Enterobacteriaceae*	17.84	0.018	320395	*G.Bacteroides.S.uniformis*	-17.237	0.010
1101669	*F.Gemellaceae*	19.12	0.021	797229	*F.Enterobacteriaceae*	-11.219	0.011
236821	*F.Enterobacteriaceae*	19.22	0.021	344154	*G.Bacteroides.S.uniformis*	-16.753	0.011
688934	*F.Enterobacteriaceae*	18.67	0.022	336012	*G.Bacteroides.S.uniformis*	-13.330	0.019
4328189	*F.Enterobacteriaceae*	13.07	0.022	4371046	*G.Bacteroides.S.uniformis*	-16.315	0.022
668514	*F.Enterobacteriaceae*	18.67	0.023	350277	*G.Bacteroides.S.uniformis*	-15.381	0.028
875735	*G.Actinomyces*	13.64	0.026	589071	*G.Bacteroides.S.uniformis*	-17.422	0.033
4376230	*F.Enterobacteriaceae*	19.33	0.026	4420408	*G.Bacteroides*	-14.922	0.034
210269	*F.Enterobacteriaceae*	18.83	0.028	362997	*G.Bacteroides*	-13.442	0.034
518002	*F.Enterobacteriaceae*	17.02	0.033	369555	*G.Ruminococcus*	-11.373	0.041
232696	*F.Enterobacteriaceae*	16.14	0.034	356760	*F.Erysipelotrichaceae*	-16.666	0.042
3439402	*G.Bacteroides.S.ovatus*	11.96	0.034	465079	*G.Staphylococcus.S.aureus*	-9.657	0.043
817734	*G.Pseudomonas*	11.65	0.038	NCROTU2904	*G.Streptococcus*	-9.237	0.045
119010	*F.Enterobacteriaceae*	15.45	0.040	716006	*G.Lactococcus*	-13.815	0.047
4305815	*G.Streptococcus*	11.97	0.043	364179	*G.Bacteroides.S.caccae*	-14.220	0.051
233220	*F.Enterobacteriaceae*	17.36	0.043	NCROTU4975	*G.Bacteroides.S.uniformis*	-14.232	0.053
466445	*F.Enterobacteriaceae*	10.80	0.048	NROTU2	*F.Erysipelotrichaceae*	-15.128	0.054
4290143	*G.Streptococcus*	12.53	0.049	1107335	*G.Acinetobacter.S.rhizosphaerae*	-10.665	0.054
152859	*F.Enterobacteriaceae*	12.51	0.050	592160	*G.Lactobacillus*	-17.631	0.054
794205	*G.Lactobacillus*	11.19	0.052	332588	*G.Bacteroides.S.uniformis*	-10.757	0.066
192342	*F.Enterobacteriaceae*	13.79	0.052	682726	*G.Eggerthella.S.lenta*	-16.092	0.069
241415	*F.Enterobacteriaceae*	16.54	0.052	181239	*G.Bacteroides.S.uniformis*	-10.119	0.070
228556	*F.Enterobacteriaceae*	14.91	0.060	581079	*G.Oscillospira*	-15.425	0.084
743120	*F.Enterobacteriaceae*	10.08	0.061	1109247	*F.Enterobacteriaceae*	-14.975	0.085
NROTU27	*F.Enterobacteriaceae*	9.31	0.062	369486	*G.Lachnospira*	-7.288	0.086
819999	*F.Enterobacteriaceae*	16.15	0.065	328472	*G.Varibaculum*	-11.501	0.099
2529285	*F.Enterobacteriaceae*	15.61	0.066				
203579	*F.Enterobacteriaceae*	14.51	0.068				
686972	*F.Enterobacteriaceae*	15.08	0.068				
1083194	*G.Streptococcus*	14.72	0.070				
164789	*F.Enterobacteriaceae*	10.19	0.070				
776980	*F.Enterobacteriaceae*	15.90	0.072				
759061	*F.Enterobacteriaceae*	14.05	0.075				
511795	*G.Streptococcus.S.anginosus*	9.66	0.079				
1110317	*G.Lactobacillus*	14.95	0.079				
NCROTU2526	*F.Enterobacteriaceae*	8.88	0.083				
681779	*F.Enterobacteriaceae*	11.61	0.084				
NCROTU4968	*G.Clostridium.S.neonatale*	7.61	0.085				
3908638	*F.Enterobacteriaceae*	14.47	0.085				
224670	*F.Enterobacteriaceae*	8.69	0.087				
342397	*G.[Ruminococcus].S.gnavus*	11.47	0.089				
813457	*F.Enterobacteriaceae*	10.34	0.092				
258785	*F.Enterobacteriaceae*	9.62	0.093				

*NROTU = New.ReferenceOTU

**NCROTU = New.CleanUp.ReferenceOTU

***O., F., G., and S. in taxonomy labels indicate that the level of taxonomy is order, family, genus, or species.

Table S11d. Relation of maternal fruit intake with infant stool microbial OTUs, in infants delivered vaginally (n = 92)

Positive Associations				Negative Associations			
OTU	Taxonomy***	Coefficient	p-value	OTU	Taxonomy***	Coefficient	p-value
10085	*F.Enterobacteriaceae*	2.22	7.3E-03	NCROTU3654	*G.Bifidobacterium*	-2.50	1.4E-03
203579	*F.Enterobacteriaceae*	2.68	0.011	3171486	*F.Enterobacteriaceae*	-3.21	1.9E-03
1625448	*F.Clostridiaceae*	1.16	0.016	102049	*G.Bifidobacterium*	-2.25	2.1E-03
236821	*F.Enterobacteriaceae*	2.63	0.019	471180	*G.Bifidobacterium*	-2.95	7.8E-03
988542	*G.Haemophilus.S.parainfluenzae*	2.41	0.031	696563	*G.Blautia.S.producta*	-2.54	9.4E-03
668514	*F.Enterobacteriaceae*	2.37	0.031	4413347	*G.Bifidobacterium*	-2.26	0.010
2529285	*F.Enterobacteriaceae*	2.36	0.037	1073276	*G.Streptococcus*	-2.11	0.011
589277	*G.Bacteroides*	1.09	0.038	813479	*G.Bifidobacterium*	-2.66	0.014
4478358	*G.Veillonella.S.dispar*	1.87	0.040	292521	*G.Bifidobacterium*	-1.06	0.016
4328189	*F.Enterobacteriaceae*	1.57	0.042	3483793	*F.Enterobacteriaceae*	-2.73	0.016
582691	*F.Clostridiaceae*	1.54	0.042	1017249	*G.Bifidobacterium*	-2.45	0.021
295411	*F.Clostridiaceae*	1.15	0.047	254938	*F.Oxalobacteraceae*	-1.38	0.022
173744	*G.Megasphaera*	1.49	0.049	NROTU36	*F.Lachnospiraceae*	-1.59	0.025
320888	*F.Clostridiaceae*	1.13	0.050	553611	*G.Bifidobacterium*	-2.37	0.027
1119540	*F.Enterobacteriaceae*	2.22	0.053	339532	*G.Bifidobacterium*	-2.31	0.031
166896	*F.Clostridiaceae*	1.13	0.054	4312969	*G.Staphylococcus*	-1.37	0.032
369429	*G.[Ruminococcus]*	1.84	0.061	365484	*O.Clostridiales*	-1.65	0.041
1105343	*F.Ruminococcaceae*	1.28	0.061	361702	*G.Ruminococcus*	-1.76	0.041
3908638	*F.Enterobacteriaceae*	2.09	0.063	484304	*G.Bifidobacterium*	-2.19	0.044
331697	*F.Enterobacteriaceae*	1.93	0.066	541299	*G.Phenylobacterium*	-1.32	0.045
801438	*F.Enterobacteriaceae*	1.95	0.066	840914	*G.Prevotella.S.copri*	-1.76	0.047
NCROTU586	*F.Enterobacteriaceae*	1.31	0.070	983335	*G.Streptococcus*	-1.46	0.048
364034	*F.Lachnospiraceae*	1.86	0.071	1142029	*G.Bifidobacterium*	-2.36	0.050
1116674	*F.Enterobacteriaceae*	1.67	0.071	4376828	*G.Bifidobacterium*	-1.66	0.057
315982	*F.Clostridiaceae*	1.27	0.073	524725	*G.Atopobium*	-2.11	0.060
303379	*F.Clostridiaceae*	0.81	0.075	289709	*G.Escherichia.S.coli*	-2.15	0.061
NROTU27	*F.Enterobacteriaceae*	1.17	0.082	132041	*G.Bifidobacterium*	-1.61	0.062
681779	*F.Enterobacteriaceae*	1.57	0.082	797229	*F.Enterobacteriaceae*	-1.10	0.064
894969	*G.Streptococcus*	1.27	0.083	577294	*G.Parabacteroides.S.distasonis*	-1.61	0.065
4352318	*F.Clostridiaceae*	0.79	0.083	997439	*G.Bifidobacterium*	-1.84	0.065
NCROTU2526	*F.Enterobacteriaceae*	1.18	0.085	4303016	*G.Streptococcus*	-2.03	0.067
233220	*F.Enterobacteriaceae*	1.92	0.096	489671	*G.Staphylococcus*	-1.71	0.072
776980	*F.Enterobacteriaceae*	1.96	0.099	589071	*G.Bacteroides.S.uniformis*	-1.97	0.073
171518	*F.Enterobacteriaceae*	1.51	0.100	503315	*G.Finegoldia*	-1.93	0.082
				555945	*F.Peptostreptococcaceae*	-1.28	0.089
				369555	*G.Ruminococcus*	-1.27	0.090
				563086	*G.[Ruminococcus]*	-1.13	0.094

*NROTU = New.ReferenceOTU

**NCROTU = New.CleanUp.ReferenceOTU

****O.*, *F.*, *G.*, and *S.* in taxonomy labels indicate that the level of taxonomy is order, family, genus, or species.

Table S11e. Relation of maternal red and processed meat intake with infant stool microbial OTUs, in infants delivered vaginally (n = 92)

Positive Associations				Negative Associations			
**OTU**	**Taxonomy*** **	**Coefficient**	**p-value**	**OTU**	**Taxonomy*** **	**Coefficient**	**p-value**
342666	*F.Clostridiaceae*	4.27	0.019	515869	*G.Faecalibacterium.S.prausnitzii*	-7.06	0.014
589071	*G.Bacteroides.S.uniformis*	7.25	0.020	4472685	*G.Streptococcus*	-6.84	0.017
NROTU25	*F.Lachnospiraceae*	4.99	0.023	579608	*G.Streptococcus*	-5.80	0.022
4447072	*G.Bacteroides.S.uniformis*	5.14	0.024	836783	*G.Shewanella*	-4.09	0.028
351231	*G.Bacteroides.S.fragilis*	5.38	0.029	4440670	*G.Veillonella*	-4.71	0.031
362539	*F.Lachnospiraceae*	5.39	0.043	134265	*G.Prevotella*	-4.69	0.031
164413	*G.Enterococcus*	3.25	0.044	320888	*F.Clostridiaceae*	-3.53	0.032
4473977	*F.Lachnospiraceae*	3.64	0.047	1106324	*F.Comamonadaceae*	-4.52	0.033
362997	*G.Bacteroides*	4.78	0.049	364034	*F.Lachnospiraceae*	-6.12	0.036
563654	*G.Lactobacillus*	3.64	0.054	173744	*G.Megasphaera*	-4.51	0.037
NCROTU4975	*G.Bacteroides.S.uniformis*	5.29	0.061	516814	*G.Streptococcus*	-3.86	0.038
344154	*G.Bacteroides.S.uniformis*	4.65	0.068	NCROTU3131	*G.Streptococcus*	-4.48	0.038
4371046	*G.Bacteroides.S.uniformis*	4.99	0.069	NROTU23	*F.Lachnospiraceae*	-6.67	0.039
604966	*G.Lactobacillus*	3.04	0.072	342427	*G.Veillonella.S.dispar*	-4.77	0.041
320395	*G.Bacteroides.S.uniformis*	4.62	0.076	941096	*G.Streptococcus*	-5.49	0.044
4060124	*G.Bacteroides*	5.34	0.078	470382	*G.Coprococcus*	-4.00	0.048
354850	*G.Bacteroides*	5.74	0.081	17309	*G.Lactobacillus*	-4.51	0.059
197072	*G.Bacteroides.S.uniformis*	4.43	0.086	548587	*G.[Eubacterium].S.dolichum*	-6.02	0.062
4457268	*F.Enterobacteriaceae*	4.20	0.092	1090059	*G.Enterococcus*	-3.33	0.063
668514	*F.Enterobacteriaceae*	5.31	0.093	903426	*G.Rothia.S.mucilaginosa*	-6.22	0.069
				12574	*G.Actinomyces*	-6.11	0.071
				328458	*G.Streptococcus*	-3.35	0.080
				2656868	*G.Bacteroides*	-3.83	0.083
				189971	*G.Blautia*	-3.21	0.084
				892845	*G.Enterococcus*	-4.03	0.087
				3228974	*F.Enterobacteriaceae*	-3.42	0.088
				NROTU11	*F.Enterobacteriaceae*	-2.62	0.095
				514272	*G.Coprococcus*	-4.78	0.099

*NROTU = New.ReferenceOTU

**NCROTU = New.CleanUp.ReferenceOTU

****O.*, *F.*, *G.*, and *S.* in taxonomy labels indicate that the level of taxonomy is order, family, genus, or species.

Table S11f. Relation of maternal MUFA:SFA ratio with infant stool microbial OTUs, in infants delivered vaginally (n = 92)

Positive Associations				Negative Associations			
**OTU**	**Taxonomy*****	**Coefficient**	**p-value**	**OTU**	**Taxonomy*****	**Coefficient**	**p-value**
92535	*G.Streptococcus*	14.27	9.4E-04	NCROTU2904	*G.Streptococcus*	-6.20	0.024
2035344	*G.Blautia*	7.31	0.012	1078207	*G.Streptococcus*	-9.01	0.028
297057	*G.Bacteroides*	7.13	0.018	369486	*G.Lachnospira*	-5.48	0.029
577170	*G.Bacteroides*	8.09	0.028	523589	*G.Clostridium.S.neonatale*	-11.31	0.033
193466	*G.Blautia*	6.36	0.032	299267	*F.Enterobacteriaceae*	-10.29	0.034
360238	*F.Erysipelotrichaceae*	8.84	0.042	503406	*G.Peptoniphilus*	-9.39	0.038
NCROTU1008	*G.Blautia*	5.40	0.047	836783	*G.Shewanella*	-5.70	0.049
4480970	*G.Bacteroides.S.caccae*	6.85	0.049	1726426	*F.Enterobacteriaceae*	-6.29	0.051
302683	*G.Blautia*	5.84	0.049	532521	*G.Peptostreptococcus.S.anaerobius*	-6.41	0.052
NROTU20	*F.Lachnospiraceae*	6.41	0.057	148620	*F.Enterobacteriaceae*	-6.65	0.055
4472685	*G.Streptococcus*	8.50	0.059	1085410	*G.Streptococcus*	-5.64	0.057
189971	*G.Blautia*	5.42	0.060	103166	*F.Enterobacteriaceae*	-4.99	0.064
3304236	*G.Bacteroides*	7.40	0.065	336012	*G.Bacteroides.S.uniformis*	-6.26	0.065
628226	*F.Peptostreptococcaceae*	5.14	0.066	465079	*G.Staphylococcus.S.aureus*	-5.21	0.067
1055212	*G.Enterococcus*	7.42	0.074	879972	*G.Streptococcus*	-5.70	0.068
15431	*G.Streptococcus*	8.53	0.075	328617	*G.Bacteroides.S.uniformis*	-6.14	0.070
920226	*G.Streptococcus*	6.66	0.077	814442	*F.Enterobacteriaceae*	-6.57	0.071
442743	*F.Enterobacteriaceae*	5.65	0.078	10085	*F.Enterobacteriaceae*	-6.70	0.072
572843	*G.Enterococcus*	8.53	0.078	380567	*G.Corynebacterium*	-6.59	0.077
364926	*G.Bacteroides*	8.84	0.083	4310208	*G.Veillonella*	-6.20	0.080
177150	*G.Bacteroides*	6.00	0.085	2202350	*G.Staphylococcus*	-5.16	0.086
183662	*G.Bacteroides*	6.94	0.086	4447072	*G.Bacteroides.S.uniformis*	-6.05	0.090
4453060	*G.Enterococcus*	7.82	0.087	254938	*F.Oxalobacteraceae*	-4.55	0.091
888300	*G.Streptococcus*	6.75	0.088	4473176	*F.Enterobacteriaceae*	-6.23	0.100
364034	*F.Lachnospiraceae*	7.62	0.096				
191251	*G.Parabacteroides.S.distasonis*	8.84	0.098				
3141094	*G.Bacteroides.S.ovatus*	6.92	0.099				

*NROTU = New.ReferenceOTU

**NCROTU = New.CleanUp.ReferenceOTU

***O., F., G., and S. in taxonomy labels indicate that the level of taxonomy is order, family, genus, or species.

Table S11g. Relation of maternal DHA intake with infant stool microbial OTUs, in infants delivered vaginally (n = 92)

Positive Associations				Negative Associations			
OTU	Taxonomy***	Coefficient	p-value	OTU	Taxonomy***	Coefficient	p-value
152859	*F.Enterobacteriaceae*	24.93	1.3E-03	1111294	*G.Escherichia.S.coli*	-32.35	2.9E-03
819999	*F.Enterobacteriaceae*	32.90	2.0E-03	231787	*F.Enterobacteriaceae*	-29.08	5.1E-03
92535	*G.Streptococcus*	27.39	2.3E-03	1109247	*F.Enterobacteriaceae*	-28.52	7.1E-03
236821	*F.Enterobacteriaceae*	30.79	2.6E-03	289709	*G.Escherichia.S.coli*	-27.77	7.9E-03
688934	*F.Enterobacteriaceae*	29.65	3.0E-03	3531225	*F.Enterobacteriaceae*	-27.51	9.0E-03
119010	*F.Enterobacteriaceae*	26.90	3.4E-03	369555	*G.Ruminococcus*	-17.78	9.3E-03
228556	*F.Enterobacteriaceae*	28.09	3.6E-03	114510	*F.Enterobacteriaceae*	-27.04	0.011
210269	*F.Enterobacteriaceae*	30.13	4.0E-03	4457268	*F.Enterobacteriaceae*	-19.47	0.014
3908638	*F.Enterobacteriaceae*	29.08	4.5E-03	1107335	*G.Acinetobacter.S.rhizosphaerae*	-16.19	0.017
754778	*F.Enterobacteriaceae*	28.01	4.6E-03	4294457	*G.Rothia.S.mucilaginosa*	-21.87	0.019
801438	*F.Enterobacteriaceae*	26.91	5.4E-03	782953	*F.Enterobacteriaceae*	-24.02	0.022
2529285	*F.Enterobacteriaceae*	28.72	5.5E-03	299267	*F.Enterobacteriaceae*	-22.84	0.023
821080	*F.Enterobacteriaceae*	28.96	5.8E-03	141145	*F.Enterobacteriaceae*	-23.24	0.026
258785	*F.Enterobacteriaceae*	19.03	6.3E-03	345362	*F.Enterobacteriaceae*	-22.56	0.027
1119540	*F.Enterobacteriaceae*	28.52	6.4E-03	3483793	*F.Enterobacteriaceae*	-23.23	0.027
164789	*F.Enterobacteriaceae*	18.68	6.5E-03	132661	*G.Enterococcus*	-22.58	0.028
203579	*F.Enterobacteriaceae*	26.34	6.7E-03	4333897	*F.Enterobacteriaceae*	-22.70	0.031
813217	*F.Enterobacteriaceae*	29.15	6.9E-03	1108656	*F.Enterobacteriaceae*	-22.40	0.033
4376230	*F.Enterobacteriaceae*	28.51	7.4E-03	797229	*F.Enterobacteriaceae*	-10.90	0.047
759061	*F.Enterobacteriaceae*	25.56	8.0E-03	581079	*G.Oscillospira*	-21.85	0.047
776980	*F.Enterobacteriaceae*	27.95	9.6E-03	521851	*G.Enterococcus*	-20.04	0.050
232696	*F.Enterobacteriaceae*	24.07	0.010	592160	*G.Lactobacillus*	-21.85	0.054
511795	*G.Streptococcus.S.anginosus*	17.20	0.010	1028632	*G.Escherichia.S.coli*	-19.64	0.055
4462083	*G.Streptococcus*	15.78	0.011	968675	*G.Haemophilus.S.parainfluenzae*	-12.19	0.056
241415	*F.Enterobacteriaceae*	25.97	0.013	588216	*F.Enterobacteriaceae*	-19.94	0.057
813457	*F.Enterobacteriaceae*	18.54	0.014	1055824	*G.Staphylococcus*	-13.06	0.060
274754	*F.Enterobacteriaceae*	22.88	0.014	1696853	*G.Enterococcus*	-11.69	0.064
1110763	*F.Enterobacteriaceae*	24.33	0.015	304641	*G.Escherichia.S.coli*	-16.16	0.067
243185	*F.Enterobacteriaceae*	25.61	0.015	132041	*G.Bifidobacterium*	-14.33	0.072
233220	*F.Enterobacteriaceae*	25.04	0.018	813944	*G.Lactobacillus*	-14.22	0.083
425721	*F.Enterobacteriaceae*	19.57	0.019	364179	*G.Bacteroides.S.caccae*	-15.37	0.089
331697	*F.Enterobacteriaceae*	22.37	0.020	563086	*G.[Ruminococcus]*	-10.54	0.090
963779	*G.Agrobacterium*	16.17	0.020	173654	*F.Enterobacteriaceae*	-15.87	0.098
1104936	*F.Enterobacteriaceae*	23.75	0.023	1019465	*G.Lactobacillus*	-13.05	0.098
NCROTU4968	*G.Clostridium.S.neonatale*	12.20	0.024				
746679	*F.Enterobacteriaceae*	22.18	0.025				
861807	*G.Corynebacterium*	21.64	0.027				
4328189	*F.Enterobacteriaceae*	15.64	0.027				
737912	*F.Enterobacteriaceae*	16.76	0.029				
1123414	*F.Enterobacteriaceae*	20.00	0.030				
518002	*F.Enterobacteriaceae*	20.81	0.035				
969149	*F.Enterobacteriaceae*	17.45	0.035				
837283	*G.Serratia*	20.82	0.038				
656517	*F.Enterobacteriaceae*	16.80	0.039				
686972	*F.Enterobacteriaceae*	21.00	0.039				
875735	*G.Actinomyces*	15.42	0.042				
224670	*F.Enterobacteriaceae*	12.71	0.042				
NROTU20	*F.Lachnospiraceae*	13.78	0.048				
1116674	*F.Enterobacteriaceae*	16.19	0.057				
2676430	*G.Veillonella.S.dispar*	18.01	0.058				
1726426	*F.Enterobacteriaceae*	12.57	0.060				
4473176	*F.Enterobacteriaceae*	14.66	0.061				
184729	*F.Lachnospiraceae*	13.44	0.076				
1108275	*G.Comamonas*	17.02	0.081				
1027587	*G.Streptococcus*	12.54	0.087				
4318990	*F.Enterobacteriaceae*	13.09	0.093				

*NROTU = New.ReferenceOTU

**NCROTU = New.CleanUp.ReferenceOTU

****O.*, *F.*, *G.*, and *S.* in taxonomy labels indicate that the level of taxonomy is order, family, genus, or species.

Table S11h. Relation of maternal EPA intake with infant stool microbial OTUs, in infants delivered vaginally (n = 92)

**Positive Associations**				**Negative Associations**			
**OTU**	**Taxonomy*** **	**Coefficient**	**p-value**	**OTU**	**Taxonomy*** **	**Coefficient**	**p-value**
152859	*F.Enterobacteriaceae*	27.99	8.0E-05	1111294	*G.Escherichia.S.coli*	-30.98	2.2E-03
4462083	*G.Streptococcus*	16.64	3.7E-03	231787	*F.Enterobacteriaceae*	-27.49	4.5E-03
258785	*F.Enterobacteriaceae*	18.45	4.4E-03	114510	*F.Enterobacteriaceae*	-26.38	7.6E-03
861807	*G.Corynebacterium*	25.25	5.3E-03	289709	*G.Escherichia.S.coli*	-25.55	8.8E-03
274754	*F.Enterobacteriaceae*	23.34	6.8E-03	3531225	*F.Enterobacteriaceae*	-24.60	0.012
236821	*F.Enterobacteriaceae*	25.10	8.8E-03	1109247	*F.Enterobacteriaceae*	-23.65	0.017
813457	*F.Enterobacteriaceae*	18.25	9.0E-03	141145	*F.Enterobacteriaceae*	-22.92	0.018
759061	*F.Enterobacteriaceae*	23.05	0.010	299267	*F.Enterobacteriaceae*	-22.00	0.018
3908638	*F.Enterobacteriaceae*	24.37	0.011	1107335	*G.Acinetobacter.S.rhizosphaerae*	-14.13	0.026
819999	*F.Enterobacteriaceae*	25.16	0.012	4457268	*F.Enterobacteriaceae*	-16.45	0.027
92535	*G.Streptococcus*	21.21	0.012	345362	*F.Enterobacteriaceae*	-20.92	0.027
232696	*F.Enterobacteriaceae*	21.73	0.013	3483793	*F.Enterobacteriaceae*	-21.54	0.028
801438	*F.Enterobacteriaceae*	22.20	0.014	369555	*G.Ruminococcus*	-13.87	0.030
813217	*F.Enterobacteriaceae*	24.71	0.014	132041	*G.Bifidobacterium*	-15.80	0.033
511795	*G.Streptococcus.S.anginosus*	15.30	0.015	782953	*F.Enterobacteriaceae*	-20.48	0.037
2529285	*F.Enterobacteriaceae*	23.65	0.015	1108656	*F.Enterobacteriaceae*	-19.85	0.043
754778	*F.Enterobacteriaceae*	22.54	0.015	4294457	*G.Rothia.S.mucilaginosa*	-17.57	0.045
210269	*F.Enterobacteriaceae*	23.88	0.015	359954	*G.Veillonella*	-10.90	0.050
119010	*F.Enterobacteriaceae*	20.85	0.016	176775	*G.Phascolarctobacterium*	-15.54	0.059
203579	*F.Enterobacteriaceae*	21.92	0.016	968675	*G.Haemophilus.S.parainfluenzae*	-11.24	0.059
688934	*F.Enterobacteriaceae*	22.66	0.016	4333897	*F.Enterobacteriaceae*	-18.33	0.063
228556	*F.Enterobacteriaceae*	21.69	0.017	592160	*G.Lactobacillus*	-19.65	0.063
837283	*G.Serratia*	21.94	0.018	132661	*G.Enterococcus*	-17.86	0.064
969149	*F.Enterobacteriaceae*	18.08	0.019	1696853	*G.Enterococcus*	-10.79	0.066
776980	*F.Enterobacteriaceae*	23.63	0.019	581079	*G.Oscillospira*	-18.37	0.074
821080	*F.Enterobacteriaceae*	22.66	0.022	1055824	*G.Staphylococcus*	-11.50	0.075
917641	*G.Staphylococcus*	20.44	0.022	1019465	*G.Lactobacillus*	-12.61	0.087
1119540	*F.Enterobacteriaceae*	22.03	0.025	521851	*G.Enterococcus*	-16.28	0.088
1116674	*F.Enterobacteriaceae*	17.63	0.026	813944	*G.Lactobacillus*	-13.02	0.088
1110763	*F.Enterobacteriaceae*	20.77	0.026	NROTU25	*F.Lachnospiraceae*	-11.30	0.089
243185	*F.Enterobacteriaceae*	21.34	0.030	1028632	*G.Escherichia.S.coli*	-16.10	0.092
241415	*F.Enterobacteriaceae*	21.17	0.031	716006	*G.Lactococcus*	-13.40	0.096
164789	*F.Enterobacteriaceae*	13.86	0.032	3583645	*G.Bacteroides*	-13.84	0.097
4473176	*F.Enterobacteriaceae*	15.59	0.032				
233220	*F.Enterobacteriaceae*	20.97	0.034				
1104936	*F.Enterobacteriaceae*	20.42	0.037				
4376230	*F.Enterobacteriaceae*	20.63	0.039				
1726426	*F.Enterobacteriaceae*	12.65	0.042				
656517	*F.Enterobacteriaceae*	15.39	0.042				
224670	*F.Enterobacteriaceae*	11.73	0.044				
331697	*F.Enterobacteriaceae*	17.79	0.049				
4318990	*F.Enterobacteriaceae*	14.13	0.051				
737912	*F.Enterobacteriaceae*	13.90	0.053				
686972	*F.Enterobacteriaceae*	17.86	0.061				
4326406	*G.Streptococcus*	11.54	0.062				
NCROTU4968	*G.Clostridium.S.neonatale*	9.25	0.068				
414943	*G.Haemophilus*	13.78	0.069				
746679	*F.Enterobacteriaceae*	16.82	0.070				
1123414	*F.Enterobacteriaceae*	15.52	0.072				
4328189	*F.Enterobacteriaceae*	11.88	0.073				
2676430	*G.Veillonella.S.dispar*	15.60	0.079				
518002	*F.Enterobacteriaceae*	16.23	0.079				
425721	*F.Enterobacteriaceae*	13.51	0.085				

*NROTU = New.ReferenceOTU

**NCROTU = New.CleanUp.ReferenceOTU

***O., F., G., and S. in taxonomy labels indicate that the level of taxonomy is order, family, genus, or species.

Table S11i. Relation of maternal nut, legume, and soy intake with infant stool microbial OTUs, in infants delivered vaginally (n = 92)

Positive Associations				Negative Associations			
**OTU**	**Taxonomy*** **	**Coefficient**	**p-value**	**OTU**	**Taxonomy*** **	**Coefficient**	**p-value**
544493	*F.Oxalobacteraceae*	2.80	3.6E-03	2415144	*G.Bacteroides*	-3.14	0.032
365484	*O.Clostridiales*	3.78	5.4E-03	312140	*G.Bacteroides*	-3.12	0.042
NCROTU835	*F.Enterobacteriaceae*	3.03	8.8E-03	364029	*G.Bacteroides*	-2.51	0.044
2035344	*G.Blautia*	2.69	0.015	1551841	*G.[Ruminococcus].S.gnavus*	-2.96	0.048
1078587	*G.Blautia*	3.23	0.016	1726426	*F.Enterobacteriaceae*	-2.42	0.048
NCROTU4968	*G.Clostridium.S.neonatale*	2.37	0.017	331575	*G.[Ruminococcus].S.gnavus*	-2.75	0.055
193466	*G.Blautia*	2.64	0.019	198788	*G.Bacteroides*	-3.54	0.061
766768	*G.Enterococcus*	4.05	0.033	4359220	*G.Veillonella.S.dispar*	-2.14	0.064
794205	*G.Lactobacillus*	2.65	0.042	757622	*G.Veillonella.S.dispar*	-3.36	0.067
572843	*G.Enterococcus*	3.70	0.044	3376513	*G.[Ruminococcus].S.gnavus*	-2.59	0.073
588471	*G.Akkermansia.S.muciniphila*	1.89	0.048	4455163	*G.Bacteroides*	-2.39	0.080
359538	*G.Bacteroides.S.caccae*	3.83	0.057	1106617	*G.Limnohabitans*	-2.53	0.081
173654	*F.Enterobacteriaceae*	3.34	0.058	NROTU36	*F.Lachnospiraceae*	-2.08	0.083
295974	*F.Clostridiaceae*	1.28	0.062	1749079	*G.Bacteroides*	-2.74	0.084
196082	*G.Blautia*	1.99	0.067	776980	*F.Enterobacteriaceae*	-3.46	0.085
189971	*G.Blautia*	1.99	0.070	587530	*G.[Eubacterium].S.dolichum*	-1.93	0.088
316675	*F.Peptostreptococcaceae*	2.75	0.074	4357712	*G.Bacteroides*	-2.67	0.090
1098340	*G.Streptococcus*	2.67	0.077				
1029949	*G.Lachnospira*	2.28	0.086				
2582263	*F.Enterobacteriaceae*	2.31	0.087				
297057	*G.Bacteroides*	1.98	0.087				
195157	*G.Bacteroides.S.ovatus*	3.46	0.088				
3663794	*G.Lactobacillus*	2.08	0.088				
183603	*G.Bacteroides.S.fragilis*	2.96	0.091				
17309	*G.Lactobacillus*	2.36	0.098				

*NROTU = New.ReferenceOTU

**NCROTU = New.CleanUp.ReferenceOTU

****O.*, *F.*, *G.*, and *S.* in taxonomy labels indicate that the level of taxonomy is order, family, genus, or species.

Table S11j. Relation of maternal PUFA intake with infant stool microbial OTUs, in infants delivered vaginally (n = 92)

Positive Associations				Negative Associations			
**OTU**	**Taxonomy*** **	**Coefficient**	**p-value**	**OTU**	**Taxonomy*** **	**Coefficient**	**p-value**
2035344	*G.Blautia*	0.87	3.2E-03	217734	*G.Streptococcus.S.anginosus*	-1.08	0.014
92535	*G.Streptococcus*	1.23	5.5E-03	606927	*F.Peptostreptococcaceae*	-0.99	0.028
794205	*G.Lactobacillus*	0.90	8.9E-03	352304	*F.Lachnospiraceae*	-0.93	0.035
196082	*G.Blautia*	0.73	0.011	364029	*G.Bacteroides*	-0.69	0.036
NCROTU1008	*G.Blautia*	0.69	0.012	NCROTU2904	*G.Streptococcus*	-0.56	0.044
295974	*F.Clostridiaceae*	0.43	0.017	712047	*F.Clostridiaceae*	-0.45	0.049
15431	*G.Streptococcus*	1.11	0.022	304641	*G.Escherichia.S.coli*	-0.83	0.055
302683	*G.Blautia*	0.64	0.033	776980	*F.Enterobacteriaceae*	-0.99	0.063
359538	*G.Bacteroides.S.caccae*	1.14	0.033	187623	*G.Bacteroides*	-0.73	0.065
198646	*G.Blautia*	0.60	0.040	1007926	*G.Streptococcus*	-0.61	0.068
364034	*F.Lachnospiraceae*	0.91	0.050	224670	*F.Enterobacteriaceae*	-0.55	0.073
1110317	*G.Lactobacillus*	1.00	0.053	3583645	*G.Bacteroides*	-0.77	0.077
316675	*F.Peptostreptococcaceae*	0.79	0.054	968675	*G.Haemophilus.S.parainfluenzae*	-0.54	0.084
193466	*G.Blautia*	0.57	0.059	196176	*G.Dorea*	-0.86	0.088
198145	*G.Blautia*	0.50	0.065	1105343	*F.Ruminococcaceae*	-0.52	0.091
572843	*G.Enterococcus*	0.90	0.068	3531225	*F.Enterobacteriaceae*	-0.87	0.095
1078587	*G.Blautia*	0.66	0.069	NROTU38	*G.Ruminococcus*	-0.54	0.097
189971	*G.Blautia*	0.53	0.072	NROTU36	*F.Lachnospiraceae*	-0.53	0.098
187035	*G.Blautia*	0.47	0.092				
342666	*F.Clostridiaceae*	0.49	0.093				
1064036	*G.Peptoniphilus*	0.78	0.096				
NROTU11	*F.Enterobacteriaceae*	0.41	0.098				

*NROTU = New.ReferenceOTU

**NCROTU = New.CleanUp.ReferenceOTU

***O., F., G., and S. in taxonomy labels indicate that the level of taxonomy is order, family, genus, or species.

Table S11k. Relation of maternal vegetable intake with infant stool microbial OTUs, in infants delivered vaginally (n = 92)

Positive Associations				Negative Associations			
**OTU**	**Taxonomy*** **	**Coefficient**	**p-value**	**OTU**	**Taxonomy*** **	**Coefficient**	**p-value**
1067519	*G.Staphylococcus*	1.88	0.011	362539	*F.Lachnospiraceae*	-1.80	4.9E-03
92535	*G.Streptococcus*	1.73	0.012	3171486	*F.Enterobacteriaceae*	-1.93	7.8E-03
997439	*G.Bifidobacterium*	1.58	0.022	696563	*G.Blautia.S.producta*	-1.75	9.8E-03
291090	*G.Parabacteroides.S.distasonis*	1.90	0.022	231787	*F.Enterobacteriaceae*	-2.01	0.011
920226	*G.Streptococcus*	1.31	0.025	181239	*G.Bacteroides.S.uniformis*	-1.25	0.016
2676430	*G.Veillonella.S.dispar*	1.56	0.029	588216	*F.Enterobacteriaceae*	-1.88	0.016
861807	*G.Corynebacterium*	1.50	0.042	114510	*F.Enterobacteriaceae*	-1.85	0.022
15431	*G.Streptococcus*	1.50	0.045	NROTU7	*G.Coprococcus*	-1.11	0.024
515869	*G.Faecalibacterium.S.prausnitzii*	1.39	0.048	2876801	*G.Bacteroides.S.uniformis*	-1.45	0.026
1063759	*G.Corynebacterium*	1.26	0.052	299267	*F.Enterobacteriaceae*	-1.67	0.027
3506872	*G.Veillonella.S.dispar*	1.22	0.053	3483793	*F.Enterobacteriaceae*	-1.72	0.030
585419	*G.Veillonella.S.dispar*	1.56	0.055	NCROTU3654	*G.Bifidobacterium*	-1.20	0.030
917641	*G.Staphylococcus*	1.38	0.057	3531225	*F.Enterobacteriaceae*	-1.71	0.033
996487	*G.Staphylococcus*	1.44	0.061	1109247	*F.Enterobacteriaceae*	-1.70	0.035
285497	*F.Caulobacteraceae*	0.91	0.065	4447072	*G.Bacteroides.S.uniformis*	-1.16	0.036
563654	*G.Lactobacillus*	0.79	0.087	211191	*F.Ruminococcaceae*	-1.02	0.038
2647328	*G.Haemophilus.S.parainfluenzae*	0.64	0.087	197072	*G.Bacteroides.S.uniformis*	-1.27	0.042
4321400	*G.Streptococcus*	1.03	0.088	345362	*F.Enterobacteriaceae*	-1.54	0.045
1082539	*G.Streptococcus*	0.65	0.093	320395	*G.Bacteroides.S.uniformis*	-1.24	0.049
888300	*G.Streptococcus*	1.03	0.095	289709	*G.Escherichia.S.coli*	-1.55	0.052
				362997	*G.Bacteroides*	-1.15	0.052
				NCROTU4975	*G.Bacteroides.S.uniformis*	-1.33	0.053
				304641	*G.Escherichia.S.coli*	-1.28	0.054
				548587	*G.[Eubacterium].S.dolichum*	-1.49	0.057
				1142029	*G.Bifidobacterium*	-1.58	0.058
				141145	*F.Enterobacteriaceae*	-1.48	0.061
				198646	*G.Blautia*	-0.84	0.061
				344154	*G.Bacteroides.S.uniformis*	-1.16	0.062
				589071	*G.Bacteroides.S.uniformis*	-1.42	0.062
				332588	*G.Bacteroides.S.uniformis*	-1.02	0.062
				782953	*F.Enterobacteriaceae*	-1.48	0.063
				646549	*G.Pseudomonas*	-0.98	0.063
				336012	*G.Bacteroides.S.uniformis*	-0.98	0.064
				2283111	*G.Bacteroides.S.uniformis*	-1.06	0.080
				328617	*G.Bacteroides.S.uniformis*	-0.92	0.083
				523589	*G.Clostridium.S.neonatale*	-1.44	0.083
				562376	*G.Dorea*	-1.11	0.084
				364179	*G.Bacteroides.S.caccae*	-1.18	0.084
				577710	*G.Blautia.S.producta*	-0.80	0.084
				554338	*G.Blautia*	-1.24	0.085
				1111294	*G.Escherichia.S.coli*	-1.44	0.085
				2689396	*F.Enterobacteriaceae*	-0.92	0.087
				348027	*G.Bacteroides.S.uniformis*	-0.96	0.090

*NROTU = New.ReferenceOTU

**NCROTU = New.CleanUp.ReferenceOTU

****O.*, *F.*, *G.*, and *S.* in taxonomy labels indicate that the level of taxonomy is order, family, genus, or species.

Table S11l. Relation of maternal whole grain intake with infant stool microbial OTUs, in infants delivered vaginally (n = 92)

Positive Associations				Negative Associations			
**OTU**	**Taxonomy*** **	**Coefficient**	**p-value**	**OTU**	**Taxonomy*** **	**Coefficient**	**p-value**
4440670	*G.Veillonella*	2.80	0.020	184729	*F.Lachnospiraceae*	-3.03	0.019
835880	*F.Enterobacteriaceae*	2.41	0.024	4426874	*G.[Ruminococcus].S.gnavus*	-3.05	0.019
1649772	*G.Escherichia.S.coli*	3.11	0.030	1839271	*G.[Ruminococcus].S.gnavus*	-3.38	0.022
4472685	*G.Streptococcus*	3.42	0.032	4385577	*F.Lachnospiraceae*	-3.42	0.027
1029949	*G.Lachnospira*	2.54	0.040	1059729	*G.Granulicatella*	-3.33	0.038
579608	*G.Streptococcus*	2.84	0.043	1551841	*G.[Ruminococcus].S.gnavus*	-2.83	0.042
2647328	*G.Haemophilus.S.parainfluenzae*	1.71	0.043	347640	*G.Blautia*	-2.13	0.044
2656868	*G.Bacteroides*	2.41	0.048	3376513	*G.[Ruminococcus].S.gnavus*	-2.69	0.046
516814	*G.Streptococcus*	2.01	0.051	331575	*G.[Ruminococcus].S.gnavus*	-2.66	0.047
963344	*G.Enhydrobacter*	2.48	0.062	327851	*G.Streptococcus*	-1.72	0.053
15431	*G.Streptococcus*	3.17	0.063	380567	*G.Corynebacterium*	-2.50	0.059
3583645	*G.Bacteroides*	2.75	0.073	369429	*G.[Ruminococcus]*	-2.92	0.061
305946	*G.Bacteroides*	2.53	0.089	1027587	*G.Streptococcus*	-2.32	0.064
				182517	*G.[Ruminococcus].S.gnavus*	-2.76	0.071
				176704	*G.[Ruminococcus].S.gnavus*	-2.42	0.071
				342380	*G.Blautia*	-1.95	0.072
				183651	*G.Blautia*	-1.81	0.080
				328617	*G.Bacteroides.S.uniformis*	-2.10	0.082
				191999	*F.Lachnospiraceae*	-2.15	0.082
				949789	*G.Enterococcus*	-1.92	0.085
				703741	*G.Lactobacillus*	-1.84	0.097
				541328	*G.Clostridium.S.neonatale*	-2.82	0.098

*NROTU = New.ReferenceOTU
**NCROTU = New.CleanUp.ReferenceOTU
***O., F., G., and S. in taxonomy labels indicate that the level of taxonomy is order, family, genus, or species.

Table S12a. Relation of maternal aMED score with infant stool microbial OTUs, in infants delivered by cesarean (n = 45)

Positive Associations				Negative Associations			
**OTU**	**Taxonomy*** **	**Coefficient**	**p-value**	**OTU**	**Taxonomy*** **	**Coefficient**	**p-value**
1696853	*G.Enterococcus*	2.61	3.3E-04	141145	*F.Enterobacteriaceae*	-4.15	2.3E-03
336632	*G.Akkermansia.S.muciniphila*	2.22	2.6E-03	369027	*F.Lachnospiraceae*	-2.28	2.3E-03
949863	*G.Lactobacillus.S.zeae*	3.34	4.0E-03	289709	*G.Escherichia.S.coli*	-4.11	2.4E-03
362767	*F.Lachnospiraceae*	3.42	5.5E-03	4472685	*G.Streptococcus*	-3.34	4.8E-03
NCROTU4061	*G.Bacteroides*	1.38	8.4E-03	114510	*F.Enterobacteriaceae*	-3.58	0.012
4473977	*F.Lachnospiraceae*	1.67	0.024	4308688	*G.Bifidobacterium*	-0.94	0.012
NROTU14	*F.Lachnospiraceae*	1.91	0.025	383714	*G.Anaerococcus*	-1.80	0.013
270094	*G.Bacteroides*	1.85	0.028	588216	*F.Enterobacteriaceae*	-3.29	0.014
377546	*F.Caulobacteraceae*	1.42	0.031	604966	*G.Lactobacillus*	-2.17	0.016
NROTU25	*F.Lachnospiraceae*	1.78	0.039	4441855	*G.Streptococcus*	-2.97	0.021
641490	*G.Enterococcus*	1.67	0.044	1109247	*F.Enterobacteriaceae*	-3.29	0.026
370183	*G.Blautia*	2.37	0.048	1085410	*G.Streptococcus*	-1.82	0.035
261241	*G.Enterococcus*	1.60	0.061	4111715	*F.Enterobacteriaceae*	-2.47	0.036
1055212	*G.Enterococcus*	1.75	0.075	4333897	*F.Enterobacteriaceae*	-2.59	0.042
10085	*F.Enterobacteriaceae*	1.85	0.077	563654	*G.Lactobacillus*	-1.91	0.044
4333020	*F.Enterobacteriaceae*	2.03	0.080	1108656	*F.Enterobacteriaceae*	-2.45	0.048
4433947	*G.Bacteroides*	0.89	0.082	538000	*F.Enterobacteriaceae*	-2.61	0.049
4349891	*G.Lactobacillus*	1.63	0.087	231787	*F.Enterobacteriaceae*	-2.70	0.055
4357712	*G.Bacteroides*	0.78	0.087	513500	*G.Streptococcus*	-2.34	0.055
187623	*G.Bacteroides*	1.38	0.090	941096	*G.Streptococcus*	-1.98	0.063
12574	*G.Actinomyces*	2.37	0.091	NCROTU3654	*G.Bifidobacterium*	-1.26	0.072
949789	*G.Enterococcus*	1.48	0.093	972033	*G.Streptococcus*	-2.23	0.074
2582263	*F.Enterobacteriaceae*	1.24	0.097	1111294	*G.Escherichia.S.coli*	-2.55	0.074
861807	*G.Corynebacterium*	2.15	0.098	916151	*G.Veillonella*	-1.54	0.076
NROTU24	*G.Enterococcus*	1.78	0.100	331253	*G.Blautia*	-1.55	0.080
				193466	*G.Blautia*	-1.64	0.083
				1064036	*G.Peptoniphilus*	-1.90	0.083
				782953	*F.Enterobacteriaceae*	-2.42	0.083
				302683	*G.Blautia*	-1.39	0.086
				797229	*F.Enterobacteriaceae*	-1.17	0.089
				347640	*G.Blautia*	-1.28	0.090
				198646	*G.Blautia*	-1.41	0.090
				180629	*G.Blautia*	-1.40	0.090
				92535	*G.Streptococcus*	-2.15	0.091
				299267	*F.Enterobacteriaceae*	-2.28	0.092
				196082	*G.Blautia*	-1.30	0.094
				2035344	*G.Blautia*	-1.34	0.094

*NROTU = New.ReferenceOTU

**NCROTU = New.CleanUp.ReferenceOTU

***O., F., G., and S. in taxonomy labels indicate that the level of taxonomy is order, family, genus, or species.

Table S12b. Relation of maternal dairy intake with infant stool microbial OTUs, in infants delivered by cesarean (n = 45)

Positive Associations				Negative Associations			
**OTU**	**Taxonomy*** **	**Coefficient**	**p-value**	**OTU**	**Taxonomy*** **	**Coefficient**	**p-value**
1107335	*G.Acinetobacter.S.rhizosphaerae*	2.11	5.0E-03	861807	*G.Corynebacterium*	-3.58	5.0E-03
4333897	*F.Enterobacteriaceae*	3.33	8.9E-03	646549	*G.Pseudomonas*	-1.65	0.015
359175	*F.Ruminococcaceae*	2.01	0.010	577710	*G.Blautia.S.producta*	-1.91	0.017
511795	*G.Streptococcus.S.anginosus*	3.08	0.010	1017249	*G.Bifidobacterium*	-2.80	0.024
1028632	*G.Escherichia.S.coli*	3.00	0.014	4473977	*F.Lachnospiraceae*	-1.69	0.025
538000	*F.Enterobacteriaceae*	3.16	0.018	1055212	*G.Enterococcus*	-2.17	0.029
588216	*F.Enterobacteriaceae*	3.17	0.021	769643	*G.Pseudomonas*	-1.89	0.029
NCROTU2292	*F.Clostridiaceae*	2.96	0.024	292521	*G.Bifidobacterium*	-1.20	0.034
173654	*F.Enterobacteriaceae*	2.47	0.024	484304	*G.Bifidobacterium*	-2.56	0.047
541328	*G.Clostridium.S.neonatale*	2.40	0.025	4433947	*G.Bacteroides*	-1.00	0.053
304641	*G.Escherichia.S.coli*	1.75	0.033	NROTU25	*F.Lachnospiraceae*	-1.69	0.056
2676432	*F.Clostridiaceae*	2.31	0.037	1075821	*G.Alloiococcus*	-2.67	0.059
529979	*F.Erysipelotrichaceae*	2.02	0.037	817734	*G.Pseudomonas*	-1.60	0.065
3531225	*F.Enterobacteriaceae*	2.81	0.044	197273	*G.Streptococcus*	-2.18	0.083
782953	*F.Enterobacteriaceae*	2.78	0.049	2656868	*G.Bacteroides*	-1.00	0.090
442743	*F.Enterobacteriaceae*	1.56	0.053	903426	*G.Rothia.S.mucilaginosa*	-2.01	0.091
4457268	*F.Enterobacteriaceae*	1.73	0.060	4316391	*G.Veillonella.S.dispar*	-2.06	0.095
295053	*F.Enterobacteriaceae*	1.94	0.061	544493	*F.Oxalobacteraceae*	-1.02	0.095
4454531	*F.Enterobacteriaceae*	1.75	0.064	997439	*G.Bifidobacterium*	-2.30	0.097
1104963	*F.Clostridiaceae*	2.12	0.067	NROTU27	*F.Enterobacteriaceae*	-1.19	0.099
1791578	*F.Enterobacteriaceae*	1.33	0.068				
289709	*G.Escherichia.S.coli*	2.62	0.068				
1147925	*F.Clostridiaceae*	1.86	0.070				
1059655	*G.Streptococcus*	1.50	0.072				
114510	*F.Enterobacteriaceae*	2.63	0.075				
356760	*F.Erysipelotrichaceae*	2.32	0.076				
345448	*F.Clostridiaceae*	2.02	0.082				
801438	*F.Enterobacteriaceae*	2.12	0.082				
1084865	*G.Staphylococcus*	2.05	0.090				
523589	*G.Clostridium.S.neonatale*	1.42	0.094				

*NROTU = New.ReferenceOTU

**NCROTU = New.CleanUp.ReferenceOTU

***O., F., G., and S. in taxonomy labels indicate that the level of taxonomy is order, family, genus, or species.

Table S12c. Relation of maternal fish and seafood intake with infant stool microbial OTUs, in infants delivered by cesarean (n = 45)

Positive Associations				Negative Associations			
OTU	Taxonomy***	Coefficient	p-value	OTU	Taxonomy***	Coefficient	p-value
302880	*G.Streptococcus*	32.89	1.9E-05	1078207	*G.Streptococcus*	-18.85	0.014
567972	*G.Streptococcus.S.agalactiae*	35.58	1.6E-03	958584	*G.Clostridium.S.neonatale*	-26.83	0.020
1076969	*G.Streptococcus*	29.87	1.8E-03	NCROTU4270	*G.Clostridium.S.neonatale*	-15.78	0.028
328283	*G.Streptococcus*	25.80	2.4E-03	NCROTU4968	*G.Clostridium.S.neonatale*	-14.00	0.030
327851	*G.Streptococcus*	25.61	2.5E-03	4111715	*F.Enterobacteriaceae*	-24.64	0.031
237444	*G.Streptococcus*	24.81	2.6E-03	4303016	*G.Streptococcus*	-23.97	0.042
332718	*G.Streptococcus*	26.98	4.3E-03	1104936	*F.Enterobacteriaceae*	-25.09	0.043
NROTU15	*G.Streptococcus*	24.10	5.4E-03	878104	*G.Veillonella.S.dispar*	-20.15	0.047
743120	*F.Enterobacteriaceae*	16.82	7.5E-03	894969	*G.Streptococcus*	-14.59	0.052
4312969	*G.Staphylococcus*	20.63	0.011	NCROTU3657	*G.Clostridium.S.butyricum*	-13.92	0.057
328617	*G.Bacteroides.S.uniformis*	7.81	0.011	541328	*G.Clostridium.S.neonatale*	-19.64	0.057
2876801	*G.Bacteroides.S.uniformis*	7.81	0.011	4376230	*F.Enterobacteriaceae*	-21.79	0.058
312140	*G.Bacteroides*	7.81	0.011	369027	*F.Lachnospiraceae*	-14.29	0.059
336012	*G.Bacteroides.S.uniformis*	7.81	0.011	1073276	*G.Streptococcus*	-17.31	0.061
194909	*G.Bacteroides*	7.81	0.011	1649772	*G.Escherichia.S.coli*	-14.70	0.071
2137001	*G.Bacteroides*	7.99	0.012	920226	*G.Streptococcus*	-18.24	0.080
3940440	*G.Bacteroides*	7.99	0.012	821080	*F.Enterobacteriaceae*	-19.12	0.099
184753	*G.Bacteroides*	7.99	0.012	236821	*F.Enterobacteriaceae*	-18.18	0.099
161423	*G.Bacteroides*	8.09	0.012				
NCROTU3323	*G.Bacteroides*	8.09	0.012				
177150	*G.Bacteroides*	8.09	0.012				
1566189	*G.Bacteroides*	8.09	0.012				
3272632	*G.Bacteroides*	8.09	0.012				
190638	*G.Bacteroides*	8.09	0.012				
199716	*G.Bacteroides*	8.17	0.012				
844375	*G.Bacteroides*	8.17	0.012				
560336	*G.Bacteroides*	8.64	0.013				
4060124	*G.Bacteroides*	8.75	0.013				
173744	*G.Megasphaera*	16.07	0.015				
513445	*G.Bacteroides*	14.69	0.018				
349024	*G.Streptococcus*	30.67	0.019				
365181	*G.Collinsella.S.aerofaciens*	17.16	0.020				
271214	*G.Bacteroides*	16.47	0.025				
548587	*G.[Eubacterium].S.dolichum*	22.20	0.040				
554338	*G.Blautia*	24.24	0.047				
587530	*G.[Eubacterium].S.dolichum*	16.83	0.053				
953855	*F.Rikenellaceae*	16.36	0.053				
4316391	*G.Veillonella.S.dispar*	21.83	0.062				
4305815	*G.Streptococcus*	9.82	0.067				
NCROTU4061	*G.Bacteroides*	9.53	0.069				
589277	*G.Bacteroides*	12.75	0.081				
NCROTU835	*F.Enterobacteriaceae*	14.38	0.086				
2283111	*G.Bacteroides.S.uniformis*	6.38	0.087				
701221	*G.Roseburia*	18.00	0.087				
1017249	*G.Bifidobacterium*	20.46	0.088				
176775	*G.Phascolarctobacterium*	14.03	0.089				
1075821	*G.Alloiococcus*	22.71	0.095				
4440670	*G.Veillonella*	12.91	0.098				

*NROTU = New.ReferenceOTU

**NCROTU = New.CleanUp.ReferenceOTU

***O., F., G., and S. in taxonomy labels indicate that the level of taxonomy is order, family, genus, or species.

Table S12d. Relation of maternal fruit intake with infant stool microbial OTUs, in infants delivered by cesarean (n = 45)

Positive Associations				Negative Associations			
**OTU**	**Taxonomy*** **	**Coefficient**	**p-value**	**OTU**	**Taxonomy*** **	**Coefficient**	**p-value**
554338	*G.Blautia*	6.18	3.9E-04	513500	*G.Streptococcus*	-5.36	1.9E-03
NROTU25	*F.Lachnospiraceae*	2.97	0.019	289709	*G.Escherichia.S.coli*	-5.53	6.4E-03
102049	*G.Bifidobacterium*	1.48	0.024	538000	*F.Enterobacteriaceae*	-4.72	0.015
949863	*G.Lactobacillus.S.zeae*	3.88	0.028	588216	*F.Enterobacteriaceae*	-4.50	0.025
342397	*G.[Ruminococcus].S.gnavus*	3.69	0.031	1064036	*G.Peptoniphilus*	-3.57	0.026
1055212	*G.Enterococcus*	3.05	0.035	4457268	*F.Enterobacteriaceae*	-2.82	0.034
NROTU14	*F.Lachnospiraceae*	2.64	0.036	231787	*F.Enterobacteriaceae*	-4.30	0.038
1027587	*G.Streptococcus*	1.74	0.049	1047077	*G.Actinomyces*	-3.52	0.040
526583	*F.Clostridiaceae*	2.67	0.049	141145	*F.Enterobacteriaceae*	-4.25	0.042
737912	*F.Enterobacteriaceae*	1.86	0.049	4454531	*F.Enterobacteriaceae*	-2.76	0.043
364926	*G.Bacteroides*	3.95	0.050	187035	*G.Blautia*	-2.61	0.046
316675	*F.Peptostreptococcaceae*	2.99	0.072	383714	*G.Anaerococcus*	-2.17	0.047
577170	*G.Bacteroides*	1.23	0.088	941096	*G.Streptococcus*	-3.10	0.048
228556	*F.Enterobacteriaceae*	2.95	0.089	2689396	*F.Enterobacteriaceae*	-2.32	0.050
641490	*G.Enterococcus*	2.09	0.090	3483793	*F.Enterobacteriaceae*	-3.71	0.053
15257	*G.Enterococcus*	3.08	0.094	295974	*F.Clostridiaceae*	-3.07	0.056
232696	*F.Enterobacteriaceae*	2.83	0.096	302683	*G.Blautia*	-2.24	0.060
NCROTU4061	*G.Bacteroides*	1.33	0.098	1147925	*F.Clostridiaceae*	-2.79	0.060
				359175	*F.Ruminococcaceae*	-2.17	0.062
				198646	*G.Blautia*	-2.28	0.063
				180629	*G.Blautia*	-2.26	0.063
				NCROTU1008	*G.Blautia*	-2.41	0.064
				15431	*G.Streptococcus*	-3.27	0.064
				1625448	*F.Clostridiaceae*	-3.15	0.066
				1109247	*F.Enterobacteriaceae*	-4.05	0.067
				2035344	*G.Blautia*	-2.15	0.067
				1108656	*F.Enterobacteriaceae*	-3.35	0.069
				196082	*G.Blautia*	-2.06	0.071
				3171486	*F.Enterobacteriaceae*	-2.78	0.073
				293342	*G.Blautia*	-2.30	0.074
				4472685	*G.Streptococcus*	-3.21	0.077
				925707	*G.Streptococcus*	-2.41	0.079
				114510	*F.Enterobacteriaceae*	-3.74	0.081
				4326406	*G.Streptococcus*	-2.38	0.084

*NROTU = New.ReferenceOTU

**NCROTU = New.CleanUp.ReferenceOTU

***O., F., G., and S. in taxonomy labels indicate that the level of taxonomy is order, family, genus, or species.

Table S12e. Relation of maternal red and processed meat intake with infant stool microbial OTUs, in infants delivered by cesarean (n = 45)

Positive Associations				Negative Associations			
**OTU**	**Taxonomy*** **	**Coefficient**	**p-value**	**OTU**	**Taxonomy*** **	**Coefficient**	**p-value**
231787	*F.Enterobacteriaceae*	16.42	1.5E-03	526682	*G.Actinomyces*	-9.61	3.7E-03
851865	*G.Faecalibacterium.S.prausnitzii*	13.84	2.5E-03	703635	*F.Enterobacteriaceae*	-8.67	4.7E-03
334656	*G.Enterococcus*	9.00	2.9E-03	835771	*F.Enterobacteriaceae*	-7.36	0.013
1028632	*G.Escherichia.S.coli*	13.26	3.8E-03	592160	*G.Lactobacillus*	-10.06	0.020
NROTU36	*F.Lachnospiraceae*	8.94	7.7E-03	4357712	*G.Bacteroides*	-3.97	0.021
1109247	*F.Enterobacteriaceae*	14.40	0.011	4328189	*F.Enterobacteriaceae*	-7.03	0.022
686789	*F.Enterococcaceae*	9.05	0.011	192342	*F.Enterobacteriaceae*	-8.16	0.027
254662	*F.Enterobacteriaceae*	12.60	0.011	315429	*G.Bacteroides*	-6.48	0.037
4433947	*G.Bacteroides*	4.64	0.016	166896	*F.Clostridiaceae*	-9.81	0.038
1111294	*G.Escherichia.S.coli*	12.73	0.019	211706	*G.Bacteroides*	-4.90	0.041
4111715	*F.Enterobacteriaceae*	10.30	0.023	3887769	*G.Bacteroides*	-4.81	0.042
299267	*F.Enterobacteriaceae*	11.59	0.024	878104	*G.Veillonella.S.dispar*	-8.08	0.045
1108656	*F.Enterobacteriaceae*	10.63	0.025	1064036	*G.Peptoniphilus*	-8.23	0.051
196176	*G.Dorea*	12.08	0.026	969149	*F.Enterobacteriaceae*	-7.37	0.052
141145	*F.Enterobacteriaceae*	12.01	0.027	NROTU20	*F.Lachnospiraceae*	-7.01	0.052
289709	*G.Escherichia.S.coli*	11.89	0.028	2875735	*G.Bacteroides*	-4.81	0.053
304641	*G.Escherichia.S.coli*	6.73	0.030	320395	*G.Bacteroides.S.uniformis*	-4.63	0.053
996487	*G.Staphylococcus*	9.61	0.032	2624257	*G.Bacteroides*	-4.75	0.055
782953	*F.Enterobacteriaceae*	11.41	0.032	712047	*F.Clostridiaceae*	-8.09	0.063
15257	*G.Enterococcus*	9.81	0.039	813457	*F.Enterobacteriaceae*	-6.58	0.066
701221	*G.Roseburia*	8.55	0.039	819999	*F.Enterobacteriaceae*	-8.37	0.068
512239	*G.Enterococcus*	10.00	0.041	656517	*F.Enterobacteriaceae*	-6.97	0.070
114510	*F.Enterobacteriaceae*	11.36	0.041	1147925	*F.Clostridiaceae*	-7.05	0.070
577294	*G.Parabacteroides.S.distasonis*	4.33	0.043	103166	*F.Enterobacteriaceae*	-4.76	0.074
797229	*F.Enterobacteriaceae*	5.32	0.044	152859	*F.Enterobacteriaceae*	-5.79	0.074
28109	*G.Bifidobacterium*	4.11	0.045	1123414	*F.Enterobacteriaceae*	-7.90	0.078
NROTU35	*G.Blautia.S.producta*	8.20	0.047	1047077	*G.Actinomyces*	-7.87	0.082
178478	*F.Rikenellaceae*	3.79	0.051	524318	*G.Bacteroides*	-8.92	0.087
949789	*G.Enterococcus*	6.54	0.053	274754	*F.Enterobacteriaceae*	-8.01	0.090
696563	*G.Blautia.S.producta*	8.17	0.055	4359220	*G.Veillonella.S.dispar*	-6.22	0.091
538000	*F.Enterobacteriaceae*	9.57	0.063	4473176	*F.Enterobacteriaceae*	-5.61	0.094
369429	*G.[Ruminococcus]*	8.87	0.064	688934	*F.Enterobacteriaceae*	-7.88	0.096
339532	*G.Bifidobacterium*	6.65	0.064				
228894	*G.Enterococcus*	7.27	0.065				
941487	*F.Oxalobacteraceae*	5.07	0.070				
572843	*G.Enterococcus*	8.73	0.075				
17976	*G.Enterococcus*	5.97	0.076				
958584	*G.Clostridium.S.neonatale*	8.24	0.077				
892845	*G.Enterococcus*	6.78	0.083				
132661	*G.Enterococcus*	8.04	0.084				
3483793	*F.Enterobacteriaceae*	8.69	0.085				
1033018	*G.Janthinobacterium.S.lividum*	4.73	0.090				
345362	*F.Enterobacteriaceae*	7.06	0.092				
442743	*F.Enterobacteriaceae*	5.17	0.093				
113773	*G.Enterococcus*	8.26	0.095				
606927	*F.Peptostreptococcaceae*	8.87	0.095				
4303016	*G.Streptococcus*	7.87	0.096				
NROTU24	*G.Enterococcus*	6.89	0.099				

*NROTU = New.ReferenceOTU

**NCROTU = New.CleanUp.ReferenceOTU

****O.*, *F.*, *G.*, and *S.* in taxonomy labels indicate that the level of taxonomy is order, family, genus, or species.

Table S12f. Relation of maternal MUFA:SFA ratio with infant stool microbial OTUs, in infants delivered by cesarean (n = 45)

Positive Associations				Negative Associations			
**OTU**	**Taxonomy*** **	**Coefficient**	**p-value**	**OTU**	**Taxonomy*** **	**Coefficient**	**p-value**
322798	*F.Clostridiaceae*	19.88	3.3E-04	NROTU36	*F.Lachnospiraceae*	-11.50	7.4E-03
337909	*G.Clostridium*	18.57	4.9E-04	356760	*F.Erysipelotrichaceae*	-16.34	8.3E-03
315982	*F.Clostridiaceae*	18.37	6.1E-04	132041	*G.Bifidobacterium*	-10.80	9.3E-03
187623	*G.Bacteroides*	12.68	8.3E-04	339532	*G.Bifidobacterium*	-11.33	0.012
582691	*F.Clostridiaceae*	19.04	1.1E-03	958584	*G.Clostridium.S.neonatale*	-14.43	0.014
148620	*F.Enterobacteriaceae*	9.50	1.3E-03	369027	*F.Lachnospiraceae*	-8.39	0.028
712047	*F.Clostridiaceae*	15.39	4.3E-03	NROTU2	*F.Erysipelotrichaceae*	-13.51	0.029
304779	*F.Clostridiaceae*	14.96	4.9E-03	604966	*G.Lactobacillus*	-9.56	0.033
355471	*F.Clostridiaceae*	15.63	5.0E-03	NCROTU4270	*G.Clostridium.S.neonatale*	-7.53	0.041
828483	*O.Clostridiales*	16.72	6.4E-03	588216	*F.Enterobacteriaceae*	-13.68	0.043
345448	*F.Clostridiaceae*	14.72	7.3E-03	682726	*G.Eggerthella.S.lenta*	-12.01	0.045
532521	*G.Peptostreptococcus.S.anaerobius*	7.26	8.6E-03	114510	*F.Enterobacteriaceae*	-14.25	0.046
1106324	*F.Comamonadaceae*	7.18	0.010	523589	*G.Clostridium.S.neonatale*	-8.06	0.049
4328189	*F.Enterobacteriaceae*	9.10	0.021	523140	*G.Ruminococcus*	-9.21	0.050
2647328	*G.Haemophilus.S.parainfluenzae*	9.03	0.023	1649772	*G.Escherichia.S.coli*	-7.79	0.060
810399	*G.Enterococcus*	11.98	0.025	NCROTU3657	*G.Clostridium.S.butyricum*	-6.94	0.062
1095073	*G.Propionibacterium.S.acnes*	9.37	0.028	851865	*G.Faecalibacterium.S.prausnitzii*	-11.25	0.066
183480	*G.Bacteroides*	7.83	0.031	1142029	*G.Bifidobacterium*	-8.91	0.070
369555	*G.Ruminococcus*	6.02	0.033	173654	*F.Enterobacteriaceae*	-9.71	0.072
198423	*G.[Ruminococcus].S.gnavus*	12.49	0.033	3908638	*F.Enterobacteriaceae*	-9.15	0.073
4475758	*G.Veillonella.S.dispar*	12.28	0.034	299267	*F.Enterobacteriaceae*	-11.73	0.079
1105343	*F.Ruminococcaceae*	5.49	0.043	819999	*F.Enterobacteriaceae*	-10.31	0.080
315429	*G.Bacteroides*	7.95	0.047	359098	*G.Bifidobacterium.S.adolescentis*	-8.99	0.081
1068499	*G.Streptococcus*	11.44	0.049	917641	*G.Staphylococcus*	-9.35	0.081
1147925	*F.Clostridiaceae*	9.76	0.049	716006	*G.Lactococcus*	-8.26	0.085
10085	*F.Enterobacteriaceae*	10.06	0.051	4413347	*G.Bifidobacterium*	-6.77	0.086
316378	*F.Clostridiaceae*	10.20	0.053	2250983	*G.Clostridium.S.neonatale*	-8.78	0.087
524725	*G.Atopobium*	11.61	0.058	1078587	*G.Blautia*	-8.33	0.088
4359220	*G.Veillonella.S.dispar*	8.81	0.061	295053	*F.Enterobacteriaceae*	-8.62	0.088
577710	*G.Blautia.S.producta*	7.22	0.069	1111294	*G.Escherichia.S.coli*	-12.04	0.089
NCROTU4696	*G.Bacteroides*	4.30	0.071	NCROTU4968	*G.Clostridium.S.neonatale*	-5.57	0.094
593672	*G.Enterococcus*	7.89	0.072	4111715	*F.Enterobacteriaceae*	-9.86	0.094
3583645	*G.Bacteroides*	4.38	0.076				
835771	*F.Enterobacteriaceae*	6.83	0.078				
350832	*F.Clostridiaceae*	8.06	0.080				
295974	*F.Clostridiaceae*	9.41	0.081				
511795	*G.Streptococcus.S.anginosus*	10.45	0.081				
292364	*G.Enterococcus*	7.51	0.091				
1111582	*G.Enterococcus*	3.92	0.091				
4357932	*G.Bacteroides.S.fragilis*	2.97	0.093				
2800178	*G.Bacteroides.S.fragilis*	2.97	0.093				
4372578	*G.Bacteroides.S.fragilis*	3.03	0.094				
2430693	*G.Bacteroides.S.fragilis*	3.11	0.095				
183603	*G.Bacteroides.S.fragilis*	3.25	0.097				
285497	*F.Caulobacteraceae*	7.21	0.098				

*NROTU = New.ReferenceOTU

**NCROTU = New.CleanUp.ReferenceOTU

****O.*, *F.*, *G.*, and *S.* in taxonomy labels indicate that the level of taxonomy is order, family, genus, or species.

Table S12g. Relation of maternal DHA intake with infant stool microbial OTUs, in infants delivered by cesarean (n = 45)

Positive Associations				Negative Associations			
**OTU**	**Taxonomy*** **	**Coefficient**	**p-value**	**OTU**	**Taxonomy*** **	**Coefficient**	**p-value**
NROTU15	*G.Streptococcus*	39.36	3.8E-04	1649772	*G.Escherichia.S.coli*	-22.88	0.032
588471	*G.Akkermansia.S.muciniphila*	18.28	7.5E-03	588216	*F.Enterobacteriaceae*	-36.94	0.035
362767	*F.Lachnospiraceae*	41.29	9.7E-03	4111715	*F.Enterobacteriaceae*	-30.97	0.041
567972	*G.Streptococcus.S.agalactiae*	38.45	0.012	141145	*F.Enterobacteriaceae*	-36.32	0.046
743120	*F.Enterobacteriaceae*	20.01	0.017	169182	*F.Enterobacteriaceae*	-20.18	0.047
302880	*G.Streptococcus*	25.63	0.022	1083508	*F.Xanthomonadaceae*	-21.87	0.047
1052663	*G.Staphylococcus*	24.44	0.022	299267	*F.Enterobacteriaceae*	-34.01	0.049
377546	*F.Caulobacteraceae*	19.15	0.024	1111294	*G.Escherichia.S.coli*	-34.91	0.056
4312969	*G.Staphylococcus*	23.81	0.029	289709	*G.Escherichia.S.coli*	-34.21	0.060
861807	*G.Corynebacterium*	35.54	0.032	369027	*F.Lachnospiraceae*	-18.73	0.062
10085	*F.Enterobacteriaceae*	28.47	0.032	231787	*F.Enterobacteriaceae*	-33.69	0.063
328617	*G.Bacteroides.S.uniformis*	8.78	0.034	114510	*F.Enterobacteriaceae*	-33.88	0.070
2876801	*G.Bacteroides.S.uniformis*	8.78	0.034	682726	*G.Eggerthella.S.lenta*	-28.25	0.071
312140	*G.Bacteroides*	8.78	0.034	1078587	*G.Blautia*	-22.34	0.078
336012	*G.Bacteroides.S.uniformis*	8.78	0.034	1104936	*F.Enterobacteriaceae*	-29.12	0.079
194909	*G.Bacteroides*	8.78	0.034	780650	*F.Clostridiaceae*	-32.70	0.079
2137001	*G.Bacteroides*	8.98	0.035	1073276	*G.Streptococcus*	-21.52	0.079
3940440	*G.Bacteroides*	8.98	0.035	4303016	*G.Streptococcus*	-27.51	0.080
184753	*G.Bacteroides*	8.98	0.035	NROTU23	*F.Lachnospiraceae*	-30.91	0.081
161423	*G.Bacteroides*	9.11	0.035	1108656	*F.Enterobacteriaceae*	-27.95	0.082
NCROTU3323	*G.Bacteroides*	9.11	0.035				
177150	*G.Bacteroides*	9.11	0.035				
1566189	*G.Bacteroides*	9.11	0.035				
3272632	*G.Bacteroides*	9.11	0.035				
190638	*G.Bacteroides*	9.11	0.035				
199716	*G.Bacteroides*	9.19	0.035				
844375	*G.Bacteroides*	9.19	0.035				
560336	*G.Bacteroides*	9.73	0.037				
4060124	*G.Bacteroides*	9.86	0.037				
852030	*G.Staphylococcus*	25.58	0.041				
320395	*G.Bacteroides.S.uniformis*	16.28	0.041				
2875735	*G.Bacteroides*	16.80	0.042				
1995363	*G.Staphylococcus.S.aureus*	25.95	0.043				
2624257	*G.Bacteroides*	16.41	0.046				
2582263	*F.Enterobacteriaceae*	18.87	0.047				
271214	*G.Bacteroides*	18.85	0.055				
2283111	*G.Bacteroides.S.uniformis*	9.39	0.056				
1696853	*G.Enterococcus*	18.87	0.059				
4328189	*F.Enterobacteriaceae*	19.23	0.064				
2724175	*G.[Ruminococcus].S.gnavus*	12.33	0.066				
NROTU20	*F.Lachnospiraceae*	22.04	0.068				
164413	*G.Enterococcus*	22.25	0.074				
1108275	*G.Comamonas*	21.37	0.080				

*NROTU = New.ReferenceOTU

**NCROTU = New.CleanUp.ReferenceOTU

***O., F., G., and S. in taxonomy labels indicate that the level of taxonomy is order, family, genus, or species.

Table S12h. Relation of maternal EPA intake with infant stool microbial OTUs, in infants delivered by cesarean (n = 45)

Positive Associations				Negative Associations			
**OTU**	**Taxonomy*** **	**Coefficient**	**p-value**	**OTU**	**Taxonomy*** **	**Coefficient**	**p-value**
NROTU15	*G.Streptococcus*	54.04	2.8E-06	889025	*G.Acinetobacter*	-33.32	4.2E-03
377546	*F.Caulobacteraceae*	26.53	3.8E-03	4416562	*F.Enterobacteriaceae*	-33.33	0.035
1095073	*G.Propionibacterium.S.acnes*	33.70	5.0E-03	1083508	*F.Xanthomonadaceae*	-23.57	0.055
320395	*G.Bacteroides.S.uniformis*	23.65	6.1E-03	4303016	*G.Streptococcus*	-33.35	0.055
2875735	*G.Bacteroides*	24.28	6.8E-03	958584	*G.Clostridium.S.neonatale*	-32.40	0.059
2624257	*G.Bacteroides*	23.80	7.6E-03	1078587	*G.Blautia*	-26.43	0.059
328617	*G.Bacteroides.S.uniformis*	11.72	9.6E-03	3908638	*F.Enterobacteriaceae*	-27.16	0.065
2876801	*G.Bacteroides.S.uniformis*	11.72	9.6E-03	801438	*F.Enterobacteriaceae*	-30.53	0.074
312140	*G.Bacteroides*	11.72	9.6E-03	92535	*G.Streptococcus*	-31.14	0.086
336012	*G.Bacteroides.S.uniformis*	11.72	9.6E-03				
194909	*G.Bacteroides*	11.72	9.6E-03				
2137001	*G.Bacteroides*	11.97	1.0E-02				
3940440	*G.Bacteroides*	11.97	1.0E-02				
184753	*G.Bacteroides*	11.97	1.0E-02				
567972	*G.Streptococcus.S.agalactiae*	43.79	1.0E-02				
161423	*G.Bacteroides*	12.12	0.010				
NCROTU3323	*G.Bacteroides*	12.12	0.010				
177150	*G.Bacteroides*	12.12	0.010				
1566189	*G.Bacteroides*	12.12	0.010				
3272632	*G.Bacteroides*	12.12	0.010				
190638	*G.Bacteroides*	12.12	0.010				
199716	*G.Bacteroides*	12.23	0.010				
844375	*G.Bacteroides*	12.23	0.010				
271214	*G.Bacteroides*	27.24	0.011				
560336	*G.Bacteroides*	12.89	0.011				
4060124	*G.Bacteroides*	13.05	0.012				
511378	*G.Veillonella*	33.66	0.013				
NCROTU1208	*G.Streptococcus*	20.43	0.014				
532521	*G.Peptostreptococcus.S.anaerobius*	19.49	0.015				
2283111	*G.Bacteroides.S.uniformis*	12.98	0.016				
362767	*F.Lachnospiraceae*	41.13	0.022				
470382	*G.Coprococcus*	27.28	0.030				
315429	*G.Bacteroides*	24.86	0.030				
3127555	*G.Bacteroides*	21.05	0.035				
1906483	*G.Bacteroides*	23.92	0.037				
1809696	*G.Bacteroides*	21.44	0.043				
302880	*G.Streptococcus*	24.92	0.046				
NROTU11	*F.Enterobacteriaceae*	16.63	0.047				
270094	*G.Bacteroides*	23.57	0.053				
861807	*G.Corynebacterium*	34.16	0.065				
NCROTU835	*F.Enterobacteriaceae*	22.62	0.065				
187623	*G.Bacteroides*	21.30	0.066				
554338	*G.Blautia*	33.08	0.066				
2582263	*F.Enterobacteriaceae*	18.60	0.080				
3141094	*G.Bacteroides.S.ovatus*	3.26	0.082				
4381553	*G.Bacteroides*	3.26	0.082				
3304236	*G.Bacteroides*	3.26	0.082				
4455163	*G.Bacteroides*	3.26	0.082				
1129060	*G.Bacteroides*	3.26	0.082				
4447072	*G.Bacteroides.S.uniformis*	3.26	0.082				
2740953	*G.Bacteroides*	3.26	0.082				
NROTU20	*F.Lachnospiraceae*	22.95	0.087				
1108275	*G.Comamonas*	22.78	0.093				
4357712	*G.Bacteroides*	10.81	0.096				

*NROTU = New.ReferenceOTU

**NCROTU = New.CleanUp.ReferenceOTU

****O.*, *F.*, *G.*, and *S.* in taxonomy labels indicate that the level of taxonomy is order, family, genus, or species.

Table S12i. Relation of maternal nut, legume, and soy intake with infant stool microbial OTUs, in infants delivered by cesarean (n = 45)

Positive Associations				Negative Associations			
**OTU**	**Taxonomy*** **	**Coefficient**	**p-value**	**OTU**	**Taxonomy*** **	**Coefficient**	**p-value**
86428	*G.Veillonella.S.dispar*	6.76	1.6E-03	851865	*G.Faecalibacterium.S.prausnitzii*	-5.58	0.010
878104	*G.Veillonella.S.dispar*	5.47	2.5E-03	1109247	*F.Enterobacteriaceae*	-6.39	0.015
962249	*G.Veillonella.S.dispar*	4.72	3.8E-03	231787	*F.Enterobacteriaceae*	-5.98	0.016
148620	*F.Enterobacteriaceae*	3.02	5.9E-03	289709	*G.Escherichia.S.coli*	-5.89	0.019
369555	*G.Ruminococcus*	2.75	6.1E-03	299267	*F.Enterobacteriaceae*	-5.49	0.022
1106324	*F.Comamonadaceae*	2.77	6.3E-03	132041	*G.Bifidobacterium*	-3.49	0.022
4318671	*G.Veillonella.S.dispar*	4.94	8.1E-03	701221	*G.Roseburia*	-4.38	0.022
4359220	*G.Veillonella.S.dispar*	4.28	0.010	1111294	*G.Escherichia.S.coli*	-5.63	0.026
712047	*F.Clostridiaceae*	5.01	0.012	173654	*F.Enterobacteriaceae*	-4.29	0.027
524725	*G.Atopobium*	5.40	0.014	1067519	*G.Staphylococcus*	-3.54	0.031
526583	*F.Clostridiaceae*	3.59	0.029	1649772	*G.Escherichia.S.coli*	-3.17	0.034
315429	*G.Bacteroides*	3.15	0.029	369027	*F.Lachnospiraceae*	-2.94	0.035
703635	*F.Enterobacteriaceae*	3.10	0.034	4457268	*F.Enterobacteriaceae*	-3.40	0.036
4478358	*G.Veillonella.S.dispar*	4.58	0.035	917641	*G.Staphylococcus*	-4.03	0.037
233220	*F.Enterobacteriaceae*	4.30	0.035	523140	*G.Ruminococcus*	-3.55	0.037
1083508	*F.Xanthomonadaceae*	3.16	0.040	3171486	*F.Enterobacteriaceae*	-3.90	0.037
315982	*F.Clostridiaceae*	4.22	0.040	1108656	*F.Enterobacteriaceae*	-4.62	0.038
337909	*G.Clostridium*	4.17	0.042	NROTU36	*F.Lachnospiraceae*	-3.28	0.040
4475758	*G.Veillonella.S.dispar*	4.09	0.053	588216	*F.Enterobacteriaceae*	-5.03	0.041
4371880	*G.Veillonella.S.dispar*	3.73	0.058	141145	*F.Enterobacteriaceae*	-5.20	0.041
304779	*F.Clostridiaceae*	3.77	0.058	NROTU35	*G.Blautia.S.producta*	-3.90	0.042
152859	*F.Enterobacteriaceae*	2.76	0.067	114510	*F.Enterobacteriaceae*	-5.22	0.044
968675	*G.Haemophilus.S.parainfluenzae*	2.78	0.068	339532	*G.Bifidobacterium*	-3.29	0.048
355471	*F.Clostridiaceae*	3.81	0.069	3483793	*F.Enterobacteriaceae*	-4.55	0.051
4334770	*G.Veillonella.S.dispar*	4.32	0.070	1028632	*G.Escherichia.S.coli*	-4.26	0.054
4374753	*G.Veillonella.S.dispar*	3.50	0.076	128382	*G.Dialister*	-4.23	0.060
187623	*G.Bacteroides*	2.56	0.079	1085410	*G.Streptococcus*	-2.94	0.060
211191	*F.Ruminococcaceae*	2.29	0.080	614083	*G.Staphylococcus*	-3.90	0.063
345448	*F.Clostridiaceae*	3.54	0.085	523589	*G.Clostridium.S.neonatale*	-2.77	0.064
2647328	*G.Haemophilus.S.parainfluenzae*	2.52	0.086	4333897	*F.Enterobacteriaceae*	-4.21	0.068
4321400	*G.Streptococcus*	2.65	0.088	15431	*G.Streptococcus*	-3.92	0.068
3506872	*G.Veillonella.S.dispar*	3.42	0.092	538000	*F.Enterobacteriaceae*	-4.30	0.073
183480	*G.Bacteroides*	2.21	0.098	356760	*F.Erysipelotrichaceae*	-4.11	0.076
285497	*F.Caulobacteraceae*	2.62	0.098	996487	*G.Staphylococcus*	-3.74	0.076
198423	*G.[Ruminococcus].S.gnavus*	3.56	0.099	667570	*F.Enterobacteriaceae*	-3.92	0.077
				782953	*F.Enterobacteriaceae*	-4.42	0.078
				4376828	*G.Bifidobacterium*	-2.38	0.081
				553611	*G.Bifidobacterium*	-4.42	0.085
				NCROTU835	*F.Enterobacteriaceae*	-2.64	0.089
				1063759	*G.Corynebacterium*	-2.89	0.091

*NROTU = New.ReferenceOTU

**NCROTU = New.CleanUp.ReferenceOTU

****O.*, *F.*, *G.*, and *S.* in taxonomy labels indicate that the level of taxonomy is order, family, genus, or species.

Table S12j. Relation of maternal PUFA intake with infant stool microbial OTUs, in infants delivered by cesarean (n = 45)

Positive Associations				Negative Associations			
**OTU**	**Taxonomy*** **	**Coefficient**	**p-value**	**OTU**	**Taxonomy*** **	**Coefficient**	**p-value**
4359220	*G.Veillonella.S.dispar*	1.42	1.6E-04	NROTU2	*F.Erysipelotrichaceae*	-1.39	8.6E-03
4328189	*F.Enterobacteriaceae*	1.12	5.9E-04	682726	*G.Eggerthella.S.lenta*	-1.28	0.012
1696853	*G.Enterococcus*	0.90	5.4E-03	523140	*G.Ruminococcus*	-0.99	0.013
NCROTU2904	*G.Streptococcus*	0.90	8.9E-03	356760	*F.Erysipelotrichaceae*	-1.30	0.016
298427	*G.Enterococcus*	1.02	9.2E-03	173654	*F.Enterobacteriaceae*	-1.10	0.017
291508	*G.Enterococcus*	1.00	0.012	716006	*G.Lactococcus*	-0.90	0.029
577710	*G.Blautia.S.producta*	0.82	0.016	554338	*G.Blautia*	-1.16	0.030
593672	*G.Enterococcus*	0.90	0.016	NCROTU1450	*F.Clostridiaceae*	-1.02	0.037
949789	*G.Enterococcus*	0.88	0.017	975306	*G.Roseburia.S.faecis*	-0.90	0.037
878104	*G.Veillonella.S.dispar*	1.03	0.020	169182	*F.Enterobacteriaceae*	-0.69	0.039
4318671	*G.Veillonella.S.dispar*	1.03	0.022	299267	*F.Enterobacteriaceae*	-1.14	0.048
743120	*F.Enterobacteriaceae*	0.63	0.025	132041	*G.Bifidobacterium*	-0.72	0.050
4371880	*G.Veillonella.S.dispar*	1.01	0.029	72820	*G.Bifidobacterium*	-0.64	0.059
3506872	*G.Veillonella.S.dispar*	1.03	0.031	604966	*G.Lactobacillus*	-0.73	0.062
524292	*G.Staphylococcus*	1.02	0.032	369027	*F.Lachnospiraceae*	-0.62	0.064
322798	*F.Clostridiaceae*	1.09	0.033	3663794	*G.Lactobacillus*	-0.58	0.072
292364	*G.Enterococcus*	0.80	0.034	4294457	*G.Rothia.S.mucilaginosa*	-0.95	0.074
888300	*G.Streptococcus*	0.97	0.037	1078587	*G.Blautia*	-0.74	0.077
103166	*F.Enterobacteriaceae*	0.59	0.044	198788	*G.Bacteroides*	-0.48	0.081
4388645	*G.Enterococcus*	0.98	0.044	780650	*F.Clostridiaceae*	-1.08	0.081
4192048	*G.Veillonella.S.dispar*	0.79	0.047	336710	*G.Bacteroides*	-0.48	0.082
641490	*G.Enterococcus*	0.70	0.050	1000592	*G.Anaerococcus*	-0.93	0.085
4433947	*G.Bacteroides*	0.43	0.050	523589	*G.Clostridium.S.neonatale*	-0.61	0.087
10085	*F.Enterobacteriaceae*	0.87	0.052	588216	*F.Enterobacteriaceae*	-1.00	0.090
701864	*G.Enterococcus*	0.87	0.054	1063759	*G.Corynebacterium*	-0.68	0.092
100039	*G.Enterococcus*	0.79	0.061	520369	*F.Clostridiaceae*	-1.02	0.096
892845	*G.Enterococcus*	0.81	0.062	1075821	*G.Alloiococcus*	-0.99	0.097
365181	*G.Collinsella.S.aerofaciens*	0.61	0.062				
2656868	*G.Bacteroides*	0.46	0.064				
192342	*F.Enterobacteriaceae*	0.76	0.065				
17976	*G.Enterococcus*	0.68	0.068				
189403	*G.[Ruminococcus].S.gnavus*	0.38	0.070				
113773	*G.Enterococcus*	0.98	0.072				
198449	*G.Bacteroides.S.caccae*	0.90	0.073				
339013	*G.Bacteroides.S.ovatus*	0.90	0.078				
4453060	*G.Enterococcus*	0.90	0.083				
4475758	*G.Veillonella.S.dispar*	0.88	0.083				
810399	*G.Enterococcus*	0.81	0.085				
1095073	*G.Propionibacterium.S.acnes*	0.64	0.087				
NCROTU3436	*G.Staphylococcus*	0.82	0.088				
234488	*G.Bacteroides*	0.27	0.089				
4334711	*G.Bacteroides*	0.26	0.090				
3745352	*G.Bacteroides*	0.25	0.090				
712047	*F.Clostridiaceae*	0.81	0.095				
261241	*G.Enterococcus*	0.61	0.097				
925707	*G.Streptococcus*	0.66	0.098				
703635	*F.Enterobacteriaceae*	0.58	0.099				

*NROTU = New.ReferenceOTU

**NCROTU = New.CleanUp.ReferenceOTU

***O., F., G., and S. in taxonomy labels indicate that the level of taxonomy is order, family, genus, or species.

Table S12k. Relation of maternal vegetable intake with infant stool microbial OTUs, in infants delivered by cesarean (n = 45)

Positive Associations				Negative Associations			
**OTU**	**Taxonomy*** **	**Coefficient**	**p-value**	**OTU**	**Taxonomy*** **	**Coefficient**	**p-value**
470382	*G.Coprococcus*	2.29	5.4E-03	4472685	*G.Streptococcus*	-3.92	3.3E-04
554338	*G.Blautia*	3.12	7.7E-03	513500	*G.Streptococcus*	-2.94	0.010
555945	*F.Peptostreptococcaceae*	2.31	0.013	1047077	*G.Actinomyces*	-2.50	0.024
1097359	*G.Acinetobacter*	1.52	0.021	4326406	*G.Streptococcus*	-1.98	0.024
NROTU24	*G.Enterococcus*	2.32	0.022	1064036	*G.Peptoniphilus*	-2.31	0.026
1055212	*G.Enterococcus*	2.09	0.025	925707	*G.Streptococcus*	-1.84	0.036
511378	*G.Veillonella*	2.05	0.025	1085410	*G.Streptococcus*	-1.70	0.040
15257	*G.Enterococcus*	2.61	0.026	1078207	*G.Streptococcus*	-1.53	0.045
226338	*G.Enterococcus*	2.18	0.033	871442	*G.Streptococcus*	-1.54	0.053
1696853	*G.Enterococcus*	1.55	0.036	3472078	*G.Bacteroides.S.fragilis*	-1.08	0.055
686789	*F.Enterococcaceae*	1.84	0.039	898871	*G.Staphylococcus*	-1.65	0.055
17976	*G.Enterococcus*	1.64	0.048	3507744	*G.Bacteroides.S.fragilis*	-1.07	0.055
1065974	*G.Enterococcus*	2.19	0.050	4377091	*G.Bacteroides.S.fragilis*	-1.05	0.055
949863	*G.Lactobacillus.S.zeae*	2.22	0.052	4441855	*G.Streptococcus*	-2.39	0.056
696563	*G.Blautia.S.producta*	2.05	0.053	4345821	*G.Bacteroides.S.fragilis*	-1.03	0.056
996487	*G.Staphylococcus*	2.15	0.054	4329112	*G.Bacteroides.S.fragilis*	-1.00	0.056
975306	*G.Roseburia.S.faecis*	1.85	0.056	4479397	*G.Bacteroides.S.fragilis*	-1.00	0.056
NCROTU3436	*G.Staphylococcus*	2.03	0.057	4356331	*G.Bacteroides.S.fragilis*	-1.02	0.056
737912	*F.Enterobacteriaceae*	1.16	0.060	4456852	*G.Bacteroides.S.fragilis*	-1.02	0.056
334656	*G.Enterococcus*	1.46	0.060	184567	*G.Bacteroides.S.fragilis*	-0.99	0.057
701221	*G.Roseburia*	1.92	0.062	2636449	*G.Bacteroides.S.fragilis*	-0.96	0.058
132661	*G.Enterococcus*	2.09	0.069	2944933	*G.Bacteroides.S.fragilis*	-1.00	0.058
701864	*G.Enterococcus*	1.81	0.075	339599	*G.Bacteroides.S.fragilis*	-1.18	0.064
356733	*G.Staphylococcus*	1.60	0.077	183651	*G.Blautia*	-1.39	0.072
641490	*G.Enterococcus*	1.40	0.078	86428	*G.Veillonella.S.dispar*	-2.01	0.094
17309	*G.Lactobacillus*	1.69	0.081	293342	*G.Blautia*	-1.39	0.097
365484	*O.Clostridiales*	1.59	0.084				
572843	*G.Enterococcus*	2.08	0.087				
593781	*G.Enterococcus*	1.62	0.089				
4333020	*F.Enterobacteriaceae*	1.85	0.096				

*NROTU = New.ReferenceOTU

**NCROTU = New.CleanUp.ReferenceOTU

***O., F., G., and S. in taxonomy labels indicate that the level of taxonomy is order, family, genus, or species.

Table S12l. Relation of maternal whole grain intake with infant stool microbial OTUs, in infants delivered by cesarean (n = 45)

Positive Associations				Negative Associations			
**OTU**	**Taxonomy*** **	**Coefficient**	**p-value**	**OTU**	**Taxonomy*** **	**Coefficient**	**p-value**
814442	*F.Enterobacteriaceae*	4.97	8.3E-04	1109247	*F.Enterobacteriaceae*	-10.02	3.6E-03
4318990	*F.Enterobacteriaceae*	6.00	4.8E-03	516814	*G.Streptococcus*	-2.85	8.6E-03
176704	*G.[Ruminococcus].S.gnavus*	3.71	6.9E-03	141145	*F.Enterobacteriaceae*	-8.21	0.014
4476604	*O.Clostridiales*	3.54	7.6E-03	114510	*F.Enterobacteriaceae*	-8.31	0.014
523589	*G.Clostridium.S.neonatale*	4.90	0.012	289709	*G.Escherichia.S.coli*	-7.65	0.022
703635	*F.Enterobacteriaceae*	4.72	0.014	128382	*G.Dialister*	-6.66	0.024
258785	*F.Enterobacteriaceae*	5.59	0.015	1111294	*G.Escherichia.S.coli*	-7.41	0.028
963779	*G.Agrobacterium*	2.99	0.015	3483793	*F.Enterobacteriaceae*	-6.50	0.035
2575651	*G.[Ruminococcus].S.gnavus*	4.05	0.015	4333897	*F.Enterobacteriaceae*	-6.23	0.041
1052663	*G.Staphylococcus*	4.78	0.015	4434268	*G.Pseudoramibacter_Eubacterium*	-3.43	0.041
331575	*G.[Ruminococcus].S.gnavus*	3.66	0.020	1108656	*F.Enterobacteriaceae*	-6.05	0.041
362767	*F.Lachnospiraceae*	6.76	0.024	548587	*G.[Eubacterium].S.dolichum*	-5.31	0.046
3044876	*G.[Ruminococcus].S.gnavus*	4.40	0.025	193466	*G.Blautia*	-4.37	0.052
2683271	*G.[Ruminococcus].S.gnavus*	4.22	0.026	360238	*F.Erysipelotrichaceae*	-3.25	0.066
380567	*G.Corynebacterium*	4.52	0.029	3583645	*G.Bacteroides*	-2.16	0.069
1551841	*G.[Ruminococcus].S.gnavus*	3.98	0.029	928249	*G.Staphylococcus*	-2.59	0.069
NROTU17	*G.[Ruminococcus].S.gnavus*	5.63	0.035	326662	*G.Bacteroides.S.uniformis*	-2.71	0.070
238205	*G.Clostridium.S.neonatale*	2.74	0.037	941487	*F.Oxalobacteraceae*	-3.12	0.071
189403	*G.[Ruminococcus].S.gnavus*	2.41	0.038	347640	*G.Blautia*	-3.23	0.072
2582263	*F.Enterobacteriaceae*	3.64	0.039	NCROTU4696	*G.Bacteroides*	-2.06	0.074
NCROTU3436	*G.Staphylococcus*	5.22	0.050	293342	*G.Blautia*	-3.69	0.077
NCROTU3131	*G.Streptococcus*	3.37	0.051	305946	*G.Bacteroides*	-2.87	0.077
290849	*F.Enterobacteriaceae*	3.70	0.052	301149	*F.Enterobacteriaceae*	-2.67	0.080
149034	*F.Enterobacteriaceae*	4.10	0.052	538000	*F.Enterobacteriaceae*	-5.58	0.081
835771	*F.Enterobacteriaceae*	3.61	0.053	231787	*F.Enterobacteriaceae*	-5.87	0.082
984924	*G.Staphylococcus*	4.60	0.056	3745352	*G.Bacteroides*	-1.44	0.082
164789	*F.Enterobacteriaceae*	4.24	0.060	4334711	*G.Bacteroides*	-1.47	0.083
813457	*F.Enterobacteriaceae*	4.15	0.061	234488	*G.Bacteroides*	-1.53	0.083
3946926	*G.Lactobacillus*	3.79	0.062	4111715	*F.Enterobacteriaceae*	-4.90	0.085
917641	*G.Staphylococcus*	4.81	0.063	696563	*G.Blautia.S.producta*	-4.55	0.087
4385577	*F.Lachnospiraceae*	4.30	0.064	782953	*F.Enterobacteriaceae*	-5.69	0.089
2724175	*G.[Ruminococcus].S.gnavus*	2.28	0.067	196176	*G.Dorea*	-5.73	0.092
852030	*G.Staphylococcus*	4.24	0.070	3171486	*F.Enterobacteriaceae*	-4.23	0.093
656517	*F.Enterobacteriaceae*	4.31	0.070	173744	*G.Megasphaera*	-2.81	0.093
3376513	*G.[Ruminococcus].S.gnavus*	3.68	0.070	577170	*G.Bacteroides*	-1.95	0.095
316132	*G.Bacteroides*	2.86	0.075	320120	*G.Bacteroides*	-2.99	0.096
614083	*G.Staphylococcus*	4.93	0.079	1068499	*G.Streptococcus*	-4.70	0.097
184729	*F.Lachnospiraceae*	2.54	0.084	1033018	*G.Janthinobacterium.S.lividum*	-2.85	0.099
288442	*G.[Ruminococcus].S.gnavus*	3.47	0.084				
1839271	*G.[Ruminococcus].S.gnavus*	3.01	0.087				
191999	*F.Lachnospiraceae*	2.36	0.092				
191251	*G.Parabacteroides.S.distasonis*	4.58	0.093				
4345397	*F.Enterobacteriaceae*	2.64	0.094				
1654474	*G.[Ruminococcus].S.gnavus*	2.47	0.097				
336632	*G.Akkermansia.S.muciniphila*	3.05	0.098				
4426874	*G.[Ruminococcus].S.gnavus*	2.82	0.098				
NCROTU2526	*F.Enterobacteriaceae*	2.08	0.099				

*NROTU = New.ReferenceOTU

**NCROTU = New.CleanUp.ReferenceOTU

****O.*, *F.*, *G.*, and *S.* in taxonomy labels indicate that the level of taxonomy is order, family, genus, or species.

Table S13. Relation of microbial community composition in six week old infants stratified by delivery mode)

Dietary Factor	*p*-value[1,2] Vaginal delivery (n = 97)	Cesarean section (n = 48)
aMED Score	0.14	0.39
Dairy	0.76	0.044
Fruit	0.035	0.66
Vegetables	0.58	0.87
Whole Grains	0.73	0.62
Fish and Seafood	0.30	0.65
Nuts, Legumes, and Soy	0.47	0.40
Red and Processed Meat	0.91	0.81
Polyunsaturated Fat	0.83	0.36
EPA	0.22	0.74
DHA	0.32	0.77
MUFA:SFA Ratio	0.28	0.30

[1]All *p*-values are determined by PERMANOVA
[2]Crude associations

Table S14. Maternal diet is related to infant gut microbiome cluster membership according to delivery mode

Dietary Factor	OR (95% CI)[1,2] Vaginal (n = 97)		Cesarean (n = 48)	
	Cluster 2	Cluster 3	Cluster 2	Cluster 3
aMED score	1.36 (0.97,1.92)	1.03 (0.80,1.33)	0.94 (0.61,1.46)	0.85 (0.57,1.26)
Dairy	0.94 (0.64,1.39)	0.93 (0.68,1.27)	1.60 (0.96,2.68)	1.52 (0.95,2.43)
Fruit	2.18 (1.21,3.93)	1.59 (0.98,2.60)	0.53 (0.24,1.16)	0.65 (0.34,1.27)
Vegetables	0.90 (0.63,1.29)	0.87 (0.65,1.16)	0.79 (0.49,1.30)	0.89 (0.59,1.35)
Whole Grains	0.84 (0.36,1.92)	1.03 (0.56,1.91)	0.46 (0.10,2.03)	1.30 (0.49,3.45)
Fish and Seafood	2.82 (0.10,82.89)	0.89 (0.05,16.88)	16.38 (0.15,1793.62)	0.02 (0.00,2.82)
Nuts, Legumes, and Soy	0.60 (0.26,1.38)	0.55 (0.29,1.05)	1.04 (0.45,2.42)	0.82 (0.36,1.88)
Red and Processed Meat	1.76 (0.44,7.02)	1.25 (0.40,3.89)	1.10 (0.22,5.44)	0.96 (0.23,4.09)
Polyunsaturated fat	0.90 (0.71,1.13)	0.89 (0.74,1.07)	0.90 (0.69,1.17)	1.10 (0.90,1.33)
EPA	0.92 (0.01,151.39)	6.60 (0.15,287.07)	0.83 (0.00,173.9)	0.00 (0.00,19.2)
DHA	0.80 (0.00,140.33)	4.05 (0.08,200.98)	3.23 (0.02,581.55)	0.05 (0.00,20.96)
MUFA:SFA ratio	2.97 (0.35,25.08)	1.94 (0.33,11.55)	1.00 (0.10,9.62)	0.87 (0.11,6.76)

[1]Cluster 1 is the reference in all models

[2]Crude OR estimates

Effect of infant age at sample collection

The age at sample collection ranges from 2.4 – 17.9 weeks, with a median of 6.1 weeks. Age at sample collection within this range is not related to microbial community structure in the overall group (GUniFrac PERMANOVA p-value = 0.4846), nor is it related in the vaginal (GUniFrac PERMANOVA p-value = 0.165) and Cesarean section (GUniFrac PERMANOVA p-value = 0.334) delivery mode groups. Adjusting for age at sample collection does not change the

associations between maternal diet and the infant gut microbiome in PERMANOVA or multinomial logistic regression analyses.

Table S15. Relation of microbial community composition in six week old infants stratified by delivery mode

	p-value[1, 2]	
**Dietary Factor**	**Vaginal delivery (n = 97)**	**Cesarean section (n = 48)**
aMED Score	0.18	0.68
Dairy	0.77	0.037
Fruit	0.032	0.77
Vegetables	0.63	0.93
Whole Grains	0.43	0.84
Fish and Seafood	0.34	0.82
Nuts, Legumes, and Soy	0.63	0.40
Red and Processed Meat	0.87	0.91
Polyunsaturated Fat	0.77	0.36
EPA	0.29	0.72
DHA	0.38	0.78
MUFA:SFA Ratio	0.71	0.46

[1]All *p*-values are determined by PERMANOVA

[2]*p*-values are adjusted for infant feeding method, maternal BMI, parity, sample collection age, and batch

Table S16. Maternal diet is related to infant gut microbiome cluster membership according to delivery mode

	OR (95% CI)[1,2]			
**Dietary Factor**	**Vaginal (n = 97)**		**Cesarean (n = 48)**	
	**Cluster 2**	**Cluster 3**	**Cluster 2**	**Cluster 3**
aMED score	1.33 (0.91,1.95)	0.99 (0.74,1.32)	0.99 (0.54,1.83)	1.03 (0.63,1.7)
Dairy	0.92 (0.57,1.46)	0.89 (0.62,1.27)	2.45 (1.03,5.81)	1.90 (0.96,3.76)
Fruit	2.79 (1.37,5.69)	1.66 (0.97,2.82)	0.57 (0.21,1.57)	0.72 (0.34,1.54)
Vegetables	0.97 (0.64,1.47)	0.89 (0.63,1.24)	0.66 (0.27,1.66)	0.95 (0.6,1.51)
Whole Grains	0.97 (0.37,2.53)	1.15 (0.59,2.27)	0.25 (0.03,1.93)	1.90 (0.51,7.07)
Fish and Seafood	0.89 (0.02,53.03)	0.75 (0.02,23.9)	33.73 (0.08,14871.35)	0.01 (0,4.42)
Nuts, Legumes, and Soy	0.53 (0.22,1.33)	0.53 (0.26,1.09)	1.03 (0.39,2.76)	0.87 (0.34,2.24)
Red and Processed Meat	4.28 (0.76,24.19)	2.22 (0.55,8.95)	1.07 (0.12,9.47)	0.85 (0.15,4.88)
Polyunsaturated fat	0.84 (0.64,1.08)	0.87 (0.72,1.06)	0.91 (0.67,1.24)	1.12 (0.90,1.4)
EPA	0.21 (0.00,69.66)	3.73 (0.07,213.32)	1.80 (0.00,7093.41)	0.00 (0.00,6.25)
DHA	0.15 (0.00,68.87)	2.70 (0.04,185.88)	4.77 (0.00,9248.6)	0.04 (0,47.67)
MUFA:SFA ratio	1.50 (0.13,16.76)	1.65 (0.23,12.07)	1.18 (0.06,23.24)	1.01 (0.08,13.02)

[1]Cluster 1 is the reference in all models

[2]All estimates are adjusted for infant feeding method, maternal BMI, parity, sample collection age, and batch
